# Comorbidity Burden in Frontal Fibrosing Alopecia and Lichen Planopilaris: A Systematic Review and Prevalence Meta-Analysis

**DOI:** 10.3390/jcm15197728

**Published:** 2026-10-07

**Authors:** Juan Luis Sanz-Cabanillas, Francisco José Gómez-García, Elena Beatriz Sanz-Cabanillas, Jesús Gay-Mimbrera, Antonio José Vélez García-Nieto, Beatriz Isla-Tejera, Juan Ruano

**Affiliations:** 1 Inflammatory Immune-Mediated Skin Diseases Laboratory (GC29), Maimonides Biomedical Research Institute of Córdoba (IMIBIC), University of Córdoba, 14004 Córdoba, Spain; fjgg79@gmail.com (F.J.G.-G.); gaymimbrera@gmail.com (J.G.-M.); avelez108@gmail.com (A.J.V.G.-N.); beatrizislatj@gmail.com (B.I.-T.); juanruanoruiz@mac.com (J.R.); 2 Department of Dermatology, Reina Sofía University Hospital, 14004 Córdoba, Spain; ebsanzc@gmail.com; 3 Department of Medical and Surgical Sciences, Faculty of Medicine and Nursing, University of Córdoba, 14004 Córdoba, Spain; 4 Department of Pharmacy, Reina Sofía University Hospital, 14004 Córdoba, Spain

**Keywords:** frontal fibrosing alopecia, lichen planopilaris, cicatricial alopecia, comorbidities, prevalence, systematic review, meta-analysis

## Abstract

**Background/Objectives:** Frontal fibrosing alopecia (FFA) and lichen planopilaris (LPP) are primary lymphocytic cicatricial alopecias with frequently reported systemic and dermatological comorbidities, although their prevalence and consistency across studies remain uncertain. **Methods:** We conducted a systematic review and random-effects prevalence meta-analysis following PRISMA 2020 and Joanna Briggs Institute guidance, prospectively registered in PROSPERO (CRD420250656067). MEDLINE, Embase, ClinicalTrials.gov, preprint sources, and reference lists were searched from inception to 18 June 2025 for observational studies reporting comorbidity prevalence in FFA and/or LPP. **Results:** Sixty studies comprising 4499 patients with FFA and 1370 with LPP were included. In FFA, the highest pooled prevalences among commonly reported conditions included androgenetic alopecia (31.9%), hypertension (23.6%), thyroid disorders (22.2%), hypothyroidism (20.6%), lichen planus pigmentosus (19.6%), and dyslipidemia (17.9%). In LPP, androgenetic alopecia (27.2%), hypertension (21.2%), hypothyroidism (18.7%), vitamin D deficiency (18.3%), dyslipidemia (16.0%), and thyroid disorders (14.5%) were prominent. Several estimates showed substantial between-study heterogeneity. Exploratory subgroup analyses identified study-level sources of heterogeneity for selected outcomes, whereas no meta-regression association remained significant after false-discovery-rate correction. **Conclusions:** These findings provide a quantitative map of comorbidity burden in FFA and LPP and support targeted, individualized clinical assessment. Because this analysis estimates prevalence within affected populations rather than comparative risk against controls, the findings should not be interpreted as evidence of excess risk or causal relationships.

## 1. Introduction

Frontal fibrosing alopecia (FFA) and lichen planopilaris (LPP) are among the most prevalent primary lymphocytic cicatricial alopecias and are characterized by chronic follicular inflammation leading to irreversible hair loss [1]. Although their clinical manifestations predominantly involve the scalp and hair follicles, accumulating observational evidence indicates a broader comorbidity burden encompassing autoimmune and thyroid diseases, cardiometabolic conditions, inflammatory dermatoses, and other hair disorders [2,3]. Defining the frequency of these comorbidities is clinically relevant for contextualizing the overall disease burden and informing individualized patient assessment.

FFA and LPP have historically been grouped within the spectrum of primary lymphocytic cicatricial alopecias [4]. Following its initial description in 1994 [5], FFA was considered a clinical variant of LPP within the spectrum of lichenoid scarring alopecias [6]. More recent clinicopathological evidence, however, supports their recognition as related but non-identical entities, with overlapping yet distinct clinical, histopathological, and biological features [3,7]. Both disorders show prominent type 1 immune activation, although FFA has been associated with a pronounced JAK3/IFN-γ signature and fibrotic remodeling [8], whereas activation of the IL-23/IL-17 axis has also been described in LPP [9]. These mechanistic observations provide biological context for studying their comorbidity profiles but do not establish shared causal pathways between cicatricial alopecia and systemic disease.

Despite increasing recognition of comorbid conditions in FFA and LPP, their epidemiological burden remains incompletely characterized. Recent consensus-based clinical recommendations have emphasized consideration of comorbidities, particularly in FFA, as part of individualized management [10]. However, the underlying evidence consists predominantly of retrospective and cross-sectional studies with variable sample sizes, population characteristics, diagnostic criteria, and methods of comorbidity ascertainment. Consequently, reported prevalence estimates vary substantially across studies [11,12], limiting the ability to determine how frequently individual conditions occur in patients with FFA or LPP and how robust these estimates are across different study settings.

Previous narrative reviews, expert statements, and systematic reviews have examined comorbidities in FFA, LPP, or broader primary cicatricial alopecia populations [11,12,13,14,15,16,17,18]. However, prevalence and association represent distinct epidemiological questions. Association-focused analyses evaluate whether a comorbidity occurs disproportionately relative to a comparator population, whereas prevalence synthesis estimates its absolute frequency within the affected population. Previous evidence syntheses have predominantly addressed associations, individual disease entities or comorbidity domains, or heterogeneous cicatricial alopecia populations [11,12,13,14,15,16,17,18]. A comprehensive prevalence-focused synthesis that analyzes FFA and LPP separately across systemic and dermatological comorbidity domains, while formally evaluating methodological quality, between-study heterogeneity, and the robustness of pooled estimates, is therefore needed.

We therefore conducted a systematic review and meta-analysis to quantify the prevalence of systemic and dermatological comorbidities in FFA and LPP. Using a prevalence-specific framework based on the Condition–Context–Population (CoCoPop) approach and JBI methodological guidance, we additionally evaluated study quality, sources of between-study heterogeneity, and the influence of individual studies on pooled estimates. Our objective was to provide a clinically interpretable quantitative map of comorbidity burden in FFA and LPP without conflating prevalence with comparative risk or causal association.

## 2. Methods

### 2.1. Study Design and Protocol Registration

This systematic review and meta-analysis was conducted in accordance with Joanna Briggs Institute (JBI) methodological guidance for systematic reviews of prevalence and incidence and reported according to the PRISMA 2020 statement [19,20,21]. The protocol was prospectively registered in the International Prospective Register of Systematic Reviews (PROSPERO; CRD420250656067). The completed PRISMA 2020 checklist is provided in Supplementary Table S11.

The literature search formed part of a broader systematic review of comorbidities across primary cicatricial alopecias, as reflected in the registered search strategy. For the present analysis, eligibility, data extraction, and quantitative synthesis were restricted to studies providing separately extractable data for FFA and/or LPP.

### 2.2. Eligibility Criteria

Eligibility criteria were defined according to the Condition–Context–Population (CoCoPop) framework recommended for prevalence reviews. The condition of interest comprised systemic and dermatological comorbidities, the population comprised patients with FFA and/or LPP, and the context included all clinical settings.

We included observational studies reporting extractable comorbidity prevalence data in patients with FFA and/or LPP, provided that their sampling framework allowed estimation of prevalence within the affected population. Diagnosis of FFA or LPP was required to be based on clinical, trichoscopic, and/or histopathological criteria. No restrictions were applied regarding language, age, sex, ethnicity, or geographical location. Full-text articles, research letters, brief reports, and conference abstracts were eligible provided that they reported original data, clearly identified FFA or LPP cases, and provided sufficient numerical information to derive comorbidity prevalence.

The primary synthesis focused on descriptive comorbidity data within FFA and LPP populations. To delimit the scope of this prevalence-focused review, studies primarily designed to estimate associations between these alopecias and comorbidities using external comparator groups were excluded. This restriction was applied according to each study’s primary objective and sampling framework, irrespective of its nominal study-design label.

We additionally excluded case reports; case series including fewer than five patients; studies of other alopecias without separately extractable FFA or LPP data; studies without extractable comorbidity data; narrative and systematic reviews; editorials and commentaries without original data; and reports with overlapping study populations. When overlapping populations were identified, the most comprehensive or methodologically informative dataset was retained.

### 2.3. Information Sources and Search Strategy

MEDLINE (via PubMed), Embase, and ClinicalTrials.gov were searched from database inception to 18 June 2025. Reference lists of included studies and relevant reviews were additionally screened to identify potentially eligible reports not retrieved through the electronic searches. No language or study-design restrictions were applied at the search stage. Complete database-specific search strategies are provided in Supplementary Table S1.

### 2.4. Study Selection and Data Extraction

Two reviewers (JLSC and FGG) independently screened titles and abstracts and subsequently assessed the full texts of potentially eligible reports. Disagreements were resolved by consensus or, when necessary, through consultation with a third reviewer.

Data extracted from eligible studies included publication characteristics, study design, country and clinical setting, diagnostic criteria, publication type, sample size, demographic characteristics, FFA or LPP subtype, comorbidity definitions, methods of comorbidity ascertainment, and the numerator and denominator required to estimate prevalence. Mean or median age and sex distribution were recorded when available. Methods of comorbidity ascertainment were categorized, when reported, as clinician diagnosis, medical-record review, administrative or ICD-based coding, laboratory-confirmed diagnosis, or patient self-report.

### 2.5. Assessment of Methodological Quality

Methodological quality was assessed using the nine-item JBI Critical Appraisal Checklist for Studies Reporting Prevalence Data. The checklist evaluates the appropriateness of the sampling frame and participant selection, sample-size adequacy, description of participants and setting, coverage of the identified sample, validity and reliability of condition ascertainment, appropriateness of statistical analysis, and response rate. Study-level assessments are reported in Supplementary Table S7.

For descriptive purposes, studies were categorized as higher quality (score ≥ 8), moderate quality (score 6–7), or lower quality (score ≤ 5), according to the number of checklist items rated as “Yes”. No study was excluded solely on the basis of methodological quality.

### 2.6. Meta-Analysis of Comorbidity Prevalence

Random-effects meta-analyses were performed separately for FFA and LPP to estimate the pooled prevalence of individual systemic and dermatological comorbidities. A pooled estimate was calculated when a comorbidity was reported in at least two independent studies. Study-specific proportions were logit-transformed and combined using the inverse-variance method. Between-study variance (τ^2^) was estimated using the DerSimonian–Laird estimator, and heterogeneity was quantified using the I^2^ statistic [22]. Confidence intervals for pooled estimates were calculated using the conventional normal approximation on the logit scale, without Hartung–Knapp adjustment. Studies reporting zero events or events in all participants were retained by applying a continuity correction of 0.5 to the event and non-event counts in those studies only [23]. Pooled estimates and their 95% confidence intervals were back-transformed using the inverse-logit function and expressed as percentages. The number of contributing studies (k) was reported for each pooled estimate.

### 2.7. Assessment of Small-Study Effects

Funnel-plot asymmetry was explored using Egger’s regression test for outcomes with at least 10 contributing studies. Given the limitations of conventional publication-bias methods in meta-analyses of proportions, these analyses were interpreted as assessments of small-study effects rather than definitive evidence of publication bias.

### 2.8. Subgroup Analyses, Meta-Regression, and Sensitivity Analyses

Exploratory subgroup analyses were conducted, when sufficient data were available, to investigate potential sources of between-study heterogeneity. Prespecified study-level characteristics included study design, data source, geographical region, sample-size category, mean-age category, histological confirmation, sex composition, and method of comorbidity ascertainment. Differences between subgroups were evaluated using tests for subgroup differences (Q-between). Multiplicity was addressed post hoc using the Benjamini–Hochberg false discovery rate (FDR) procedure, applied jointly to all computable subgroup-difference tests across FFA and LPP, including non-significant tests; analyses repeated across comorbidity categories were counted once. FDR-adjusted *p* values are reported as q values, with q < 0.05 considered statistically significant.

Leave-one-out sensitivity analyses were performed by sequentially omitting each contributing study to assess the influence of individual studies on pooled prevalence estimates and heterogeneity.

Univariable random-effects meta-regression was additionally performed for eligible outcomes using study-level moderators. Models were fitted using study-level proportions on the logit scale with Hartung–Knapp-type inference. Multivariable meta-regression was not undertaken because of the limited number of studies per outcome and the resulting risk of overfitting. Because multiple exploratory moderator analyses were performed, *p* values were adjusted using the Benjamini–Hochberg false discovery rate (FDR) procedure. Detailed results of subgroup analyses, meta-regression, and leave-one-out analyses are provided in Supplementary Tables S8–S10.

### 2.9. Exploratory Comparison of Pooled Prevalence Between FFA and LPP

For comorbidities with pooled estimates based on at least three independent studies in each disease group, we performed an exploratory indirect comparison of the separately pooled prevalence estimates for FFA and LPP. Descriptive prevalence ratios were calculated by comparing the pooled prevalence in FFA with the corresponding pooled prevalence in LPP and were displayed on a logarithmic scale. Values greater than 1 indicated a higher pooled prevalence in FFA, whereas values below 1 indicated a higher pooled prevalence in LPP.

Because these ratios were derived from independently pooled study populations rather than within-study head-to-head comparisons, they were considered hypothesis-generating and should not be interpreted as formal comparative effect estimates, evidence of excess risk, or causal associations. Results are presented in Supplementary Figure S4.

### 2.10. Sex–Stratified Prevalence Analyses

Post hoc sex-stratified prevalence analyses were conducted separately for FFA and classic LPP (Supplementary Table S6). Eligible data comprised single-sex samples and mixed-sex studies with explicitly reported or unambiguously attributable sex-specific event counts and denominators. Each independent cohort contributed once to a given diagnosis–sex–comorbidity analysis. Pooled estimates required at least two independent studies within the relevant stratum and used the same model as the primary analysis. Sex-specific estimates were interpreted descriptively; no formal between-sex or sex-by-diagnosis interaction tests were performed.

### 2.11. Statistical Analysis

All statistical analyses were performed using R version 4.4.3 (R Foundation for Statistical Computing, Vienna, Austria). Meta-analyses, heterogeneity analyses, subgroup analyses, meta-regression, and sensitivity analyses were conducted primarily using the meta package, and graphical outputs were generated using ggplot2 and customized R workflows. Statistical tests were two-sided. Multiplicity in exploratory subgroup and meta-regression analyses was addressed using the Benjamini–Hochberg FDR procedure.

### 2.12. Use of Artificial Intelligence

ChatGPT (GPT-5.2; OpenAI) was used to support language editing, troubleshooting of R code, and data-visualization workflows.

## 3. Results

### 3.1. Study Selection and Characteristics of Included Studies

The systematic search identified 5295 records, of which 139 reports underwent full-text assessment. Thirty-nine studies met the eligibility criteria through database screening, and 21 additional studies were identified through citation searching, yielding 60 included studies published between 2009 and 2025. Of these, 46 reported FFA-only data, 10 LPP-only data, and four reported separately extractable data for both conditions. Overall, the prevalence synthesis included data from 4499 patients with FFA and 1370 patients with LPP. Individual study sample sizes ranged from 7 to 711 participants for FFA and from 10 to 246 for LPP.

**Figure 1:**
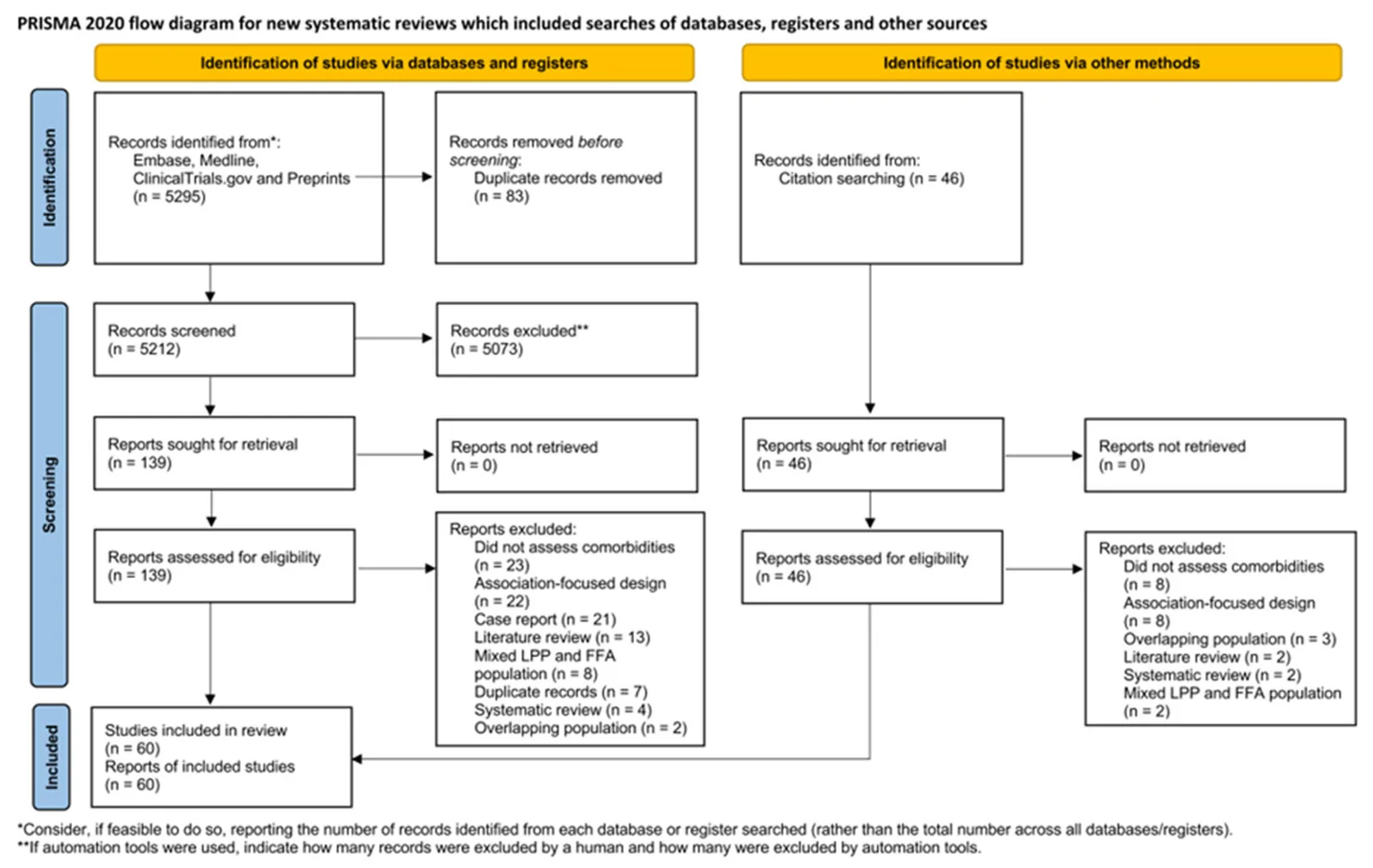
PRISMA 2020 flow diagram of study selection [21]. Flow diagram showing the identification, screening, eligibility assessment, and inclusion of studies in the systematic review and meta-analysis, including records identified through database searching and citation tracking and reasons for full-text exclusion.

The study selection process is shown in the PRISMA flow diagram (Figure 1). Included and excluded studies are listed in Supplementary Tables S2 and S3, respectively, detailed study characteristics and comorbidities reported are provided in Supplementary Tables S4 and S5. The temporal and geographical distributions of the evidence base and the frequency of reported comorbidity domains are summarized in Supplementary Figures S1–S3.

### 3.2. Methodological Quality

Methodological quality was predominantly moderate. JBI scores ranged from 5 to 8 across the 60 included studies, with most studies scoring 6 or 7 and a smaller proportion reaching a score of 8. Recurrent limitations involved uncertainty regarding the representativeness of the sampling frame, sample-size adequacy, and response-rate reporting. Detailed study-level assessments are provided in Supplementary Table S7.

### 3.3. Pooled Prevalence of Comorbidities

#### Lichen Planopilaris

Among patients with LPP, several cardiometabolic conditions were frequently reported. Pooled prevalence was 21.2% (95% CI, 13.5–31.6) for hypertension, 16.0% (95% CI, 11.3–22.3) for dyslipidemia, 12.7% (95% CI, 7.2–21.5) for obesity, and 6.8% (95% CI, 4.5–10.3) for diabetes. Coronary artery disease had a pooled prevalence of 6.8% (95% CI, 4.3–10.5).

Thyroid disorders were also frequent, with pooled prevalences of 14.5% (95% CI, 7.4–26.3) for overall thyroid disease and 18.7% (95% CI, 12.2–27.5) for hypothyroidism. Hashimoto disease was estimated at 8.0% (95% CI, 3.3–18.1).

**Figure 2:**
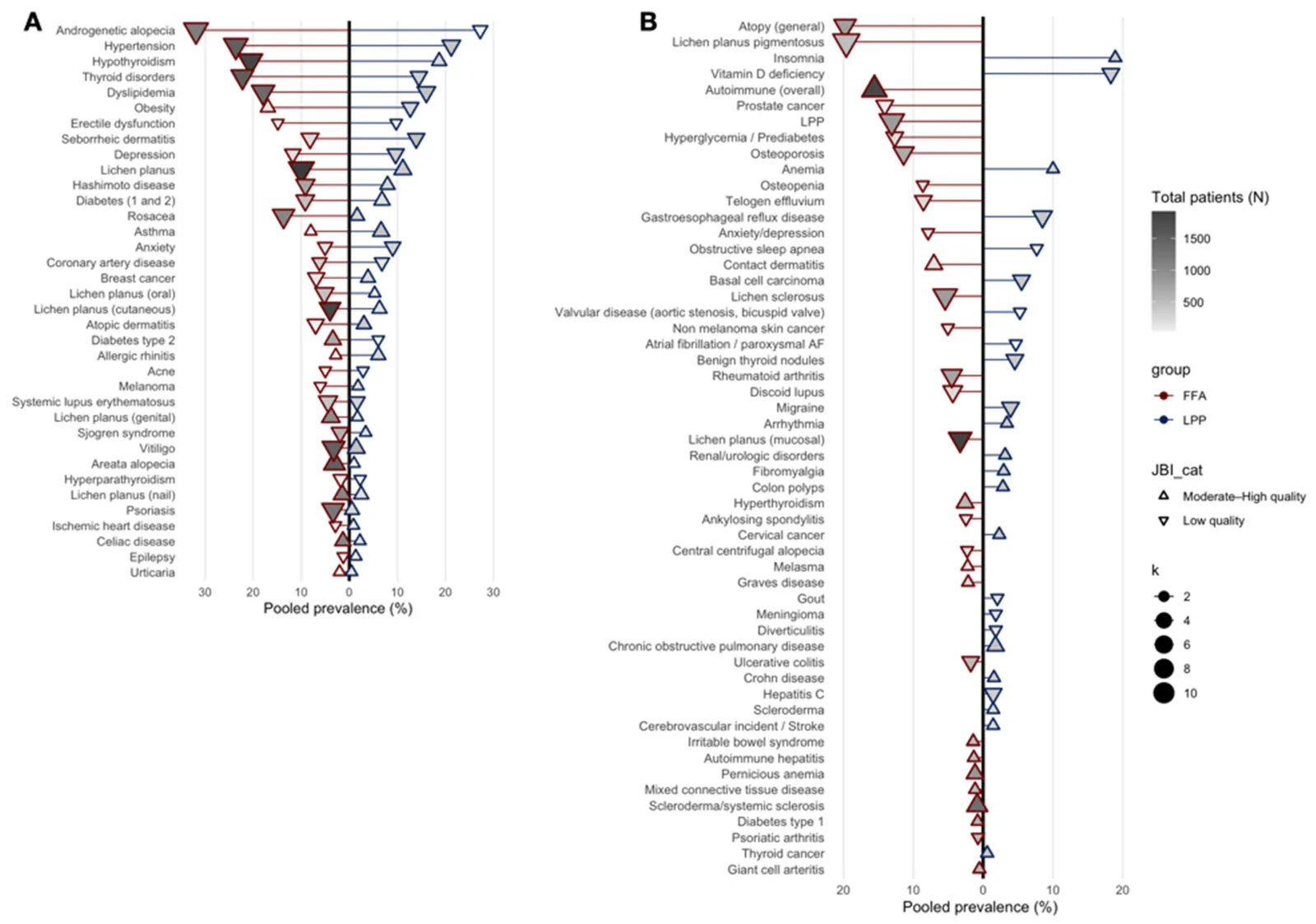
Comparative visualization of pooled comorbidity prevalence in frontal fibrosing alopecia and lichen planopilaris. (**A**) Mirror plot showing pooled prevalence estimates for core comorbidities in frontal fibrosing alopecia (FFA) and lichen planopilaris (LPP). FFA estimates are displayed to the left and LPP estimates to the right of the central axis. (**B**) Extended mirror plot incorporating additional comorbidities with available pooled estimates. Distance from the central axis represents pooled prevalence. Triangle size represents the number of contributing studies (*k*), and grayscale fill intensity represents the total number of participants contributing to each estimate (N). Upward-pointing triangles indicate moderate-to-high methodological quality, whereas downward-pointing triangles indicate low quality according to the Joanna Briggs Institute assessment. The circles form the size legend for the number of contributing studies (*k*). Comorbidities are ordered to facilitate comparison of prevalence patterns across the two conditions. Estimates based on few studies and/or heterogeneous evidence should be interpreted cautiously. FFA, frontal fibrosing alopecia; LPP, lichen planopilaris; JBI_cat, methodological quality category according to the Joanna Briggs Institute checklist.

Among dermatological and hair disorders, androgenetic alopecia showed the highest pooled prevalence at 27.2% (95% CI, 10.3–55.0). Lichen planus was present in 11.1% (95% CI, 8.0–15.3), seborrheic dermatitis in 13.9% (95% CI, 5.4–31.3), and vitamin D deficiency in 18.3% (95% CI, 8.4–35.2). Depression and anxiety had pooled prevalences of 9.6% (95% CI, 2.3–32.2) and 9.0% (95% CI, 1.9–33.9), respectively, although both estimates showed substantial heterogeneity. Complete pooled estimates for LPP are presented in Table 1 and Figure 2.

### 3.4. Frontal Fibrosing Alopecia

In FFA, androgenetic alopecia was among the most frequently reported comorbid conditions, with a pooled prevalence of 31.9% (95% CI, 23.5–41.7). Other hair disorders included LPP at 13.1% (95% CI, 9.2–18.2), telogen effluvium at 8.5% (95% CI, 1.4–38.4), and alopecia areata at 3.1% (95% CI, 1.8–5.2).

Thyroid and cardiometabolic comorbidities were also prominent. Overall thyroid disorders had a pooled prevalence of 22.2% (95% CI, 15.5–30.9), hypothyroidism 20.6% (95% CI, 16.3–25.7), and Hashimoto disease 9.1% (95% CI, 5.7–14.2). Hypertension occurred in 23.6% (95% CI, 17.8–30.6), dyslipidemia in 17.9% (95% CI, 13.4–23.5), obesity in 16.9% (95% CI, 12.0–23.3), and diabetes in 9.2% (95% CI, 3.9–19.9).

Within the lichen spectrum, pooled prevalence was 19.6% (95% CI, 11.9–30.5) for lichen planus pigmentosus and 10.0% (95% CI, 7.1–13.7) for overall lichen planus. Among inflammatory dermatoses, rosacea was present in 13.7% (95% CI, 8.2–22.0), atopic dermatitis in 7.0% (95% CI, 4.2–11.5), psoriasis in 3.4% (95% CI, 2.1–5.4), and vitiligo in 3.3% (95% CI, 2.1–5.0).

**Figure 3:**
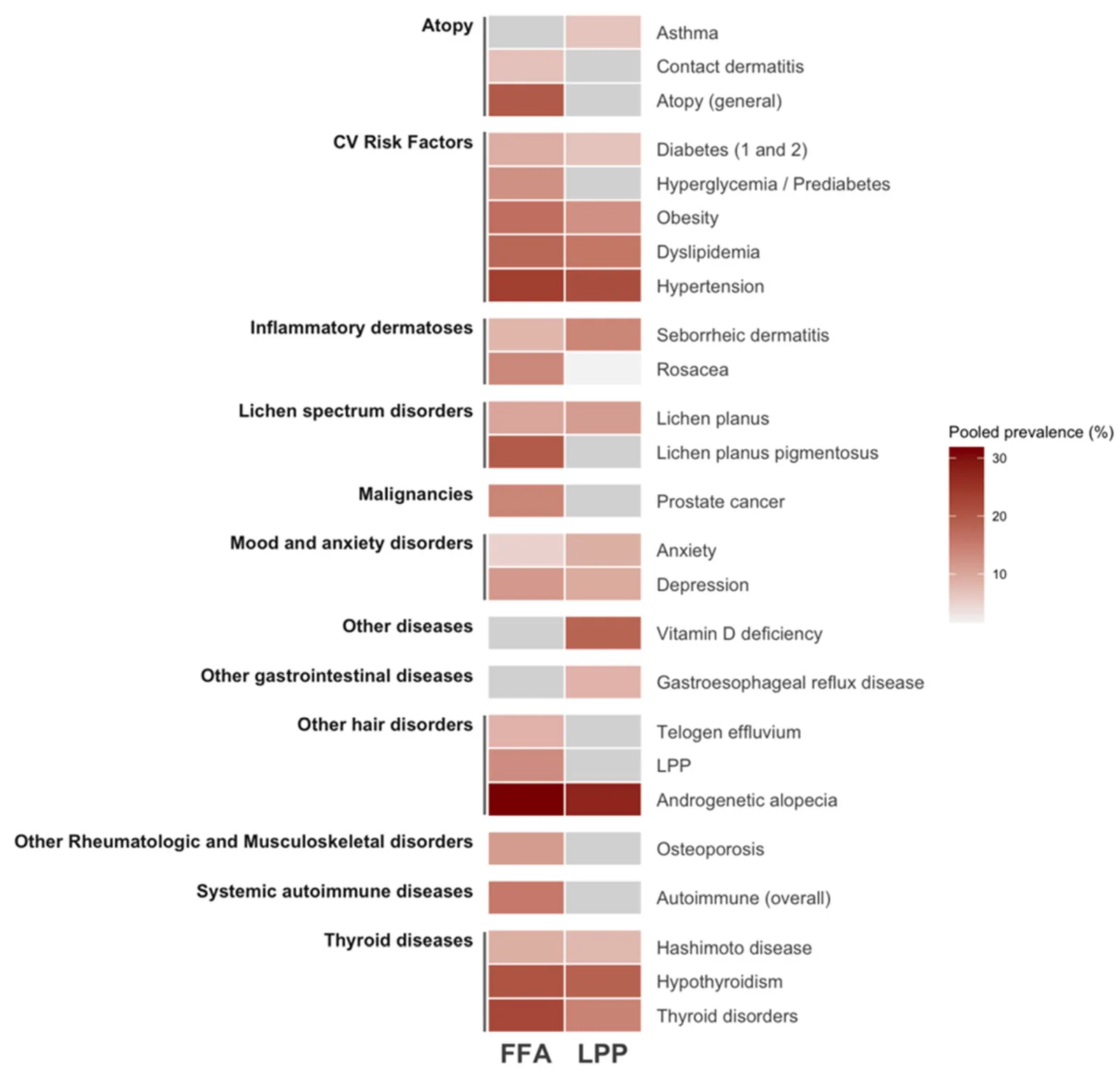
Global patterns of pooled comorbidity prevalence in frontal fibrosing alopecia and lichen planopilaris. Heatmap showing pooled prevalence estimates for individual comorbidities in frontal fibrosing alopecia (FFA) and lichen planopilaris (LPP), grouped by major clinical domain. Color intensity represents pooled prevalence, with darker shades indicating higher estimates; gray cells indicate that no pooled estimate was available. The heatmap provides a descriptive overview of shared and differing reported comorbidity patterns and should not be interpreted as a formal comparison between FFA and LPP.

Systemic autoimmune disease overall had a pooled prevalence of 15.6% (95% CI, 9.1–25.4), whereas estimates for individual autoimmune diseases were generally lower. Depression and anxiety were estimated at 11.8% (95% CI, 3.6–32.4) and 5.0% (95% CI, 1.6–14.5), respectively. Complete pooled estimates for FFA are presented in Table 2 and Figure 3.

### 3.5. Subgroup Analysis, Meta-Regression and Sensitivity Analysis

Substantial between-study heterogeneity was observed for several pooled estimates, particularly those involving broadly defined comorbidities or outcomes reported across heterogeneous clinical settings. Following Benjamini–Hochberg FDR correction, 10 of the 18 subgroup comparisons presented in Supplementary Table S8 remained statistically significant, all in FFA. These included dyslipidemia according to mean-age category (q = 0.003), rosacea and vitiligo according to study sample-size category (q = 0.045 and q = 0.010, respectively), and lichen planus pigmentosus according to geographical region (q = 0.003). Differences in hypothyroidism according to sex composition (q = 0.003) and Hashimoto disease according to histological confirmation (q < 0.001) also remained significant. Differences in hypertension and psoriasis according to variable collection method in FFA (q = 0.130 and q = 0.096, respectively), and in hypertension and gastroesophageal reflux disease according to study sample-size category in LPP (q = 0.076 and q = 0.145, respectively), were not significant after correction. These findings remain exploratory and represent study-level associations.

Univariable meta-regression identified nominal moderator effects for several FFA outcomes. The strongest nominal signals included vitiligo (*p* = 0.002), hypothyroidism (*p* = 0.002), lichen planus (cutaneous) (*p* = 0.010), rosacea (*p* = 0.015), alopecia areata (*p* = 0.015), hypertension (*p* = 0.016), and psoriasis (*p* = 0.019). However, none remained statistically significant after Benjamini–Hochberg FDR correction (all q ≥ 0.220). Reductions in residual heterogeneity varied substantially across models, with larger reductions for selected outcomes such as vitiligo and psoriasis, although these findings remained exploratory. Complete meta-regression results are provided in Supplementary Table S9.

Leave-one-out analyses showed that several estimates based on small numbers of studies or displaying substantial heterogeneity were sensitive to individual influential studies. In FFA, notable changes after study omission were observed for atopy, diabetes, depression, telogen effluvium, and selected low-frequency autoimmune and gastrointestinal outcomes. In LPP, sensitivity was observed for dyslipidemia, depression, anxiety, thyroid disorders, hypothyroidism, vitamin D deficiency, and androgenetic alopecia. Nevertheless, omission of individual studies generally did not alter the overall pattern of the most consistently reported comorbidities. Detailed leave-one-out results are provided in Supplementary Table S10.

### 3.6. Exploratory Comparison Between FFA and LPP

Exploratory indirect comparisons of separately pooled prevalence estimates suggested differences in the reported comorbidity profiles of FFA and LPP for selected conditions (Supplementary Figure S4). These comparisons were based on prevalence ratios derived from independently pooled estimates and were therefore considered descriptive and hypothesis-generating rather than formal between-disease effect estimates. Given the heterogeneity of the underlying studies and the absence of within-study head-to-head comparisons for most outcomes, no inferential conclusions regarding differential disease risk were drawn.

### 3.7. Sex-Stratified Analyses

Sex-stratified analyses yielded 78 pooled estimates: 48 among women with FFA, 23 among men with FFA, and seven among women with classic LPP. Seventeen comorbidities had pooled estimates for both sexes within FFA. The pooled prevalence of androgenetic alopecia was 27.2% (95% CI, 14.6–45.0; seven studies, 672 participants) in women and 58.0% (95% CI, 39.9–74.2; nine studies, 137 participants) in men. Hypothyroidism prevalence was 26.0% (95% CI, 17.1–37.6; nine studies, 1217 participants) in women and 6.9% (95% CI, 3.5–12.9; six studies, 127 participants) in men. Rosacea prevalence was 10.5% (95% CI, 3.7–26.2; six studies, 826 participants) in women and 28.1% (95% CI, 20.4–37.3; five studies, 115 participants) in men. In classic LPP, all seven pooled estimates in women were based on two studies, whereas no comorbidity had sufficient independent data for a primary pooled estimate in men. Detailed sex-stratified results are provided in Supplementary Table S6.

## 4. Discussion

In this systematic review and prevalence meta-analysis, FFA and LPP showed broad and heterogeneous systemic and dermatological comorbidity profiles, encompassing thyroid and cardiometabolic conditions, autoimmune and inflammatory diseases, lichen-spectrum disorders, other hair disorders, and neuropsychiatric conditions. Importantly, these estimates quantify comorbidity burden within affected populations rather than comparative risk against unaffected controls. They should therefore be interpreted as measures of absolute prevalence and not as evidence of excess risk or causal relationships.

Although FFA and LPP share a lymphocytic cicatricial alopecia background and overlapping lichenoid histopathological features, their reported comorbidity profiles were not identical. In FFA, prominent findings included androgenetic alopecia, thyroid disease and hypothyroidism, hypertension and dyslipidemia, lichen planus pigmentosus, rosacea, and systemic autoimmune disease. In LPP, androgenetic alopecia, hypertension, hypothyroidism and other thyroid disorders, dyslipidemia, vitamin D deficiency, lichen planus variants, and mood or anxiety disorders were frequently reported. Exploratory indirect comparisons also suggested differences in selected prevalence patterns between the two conditions. However, these comparisons were derived from independently pooled study populations rather than within-study head-to-head comparisons and should therefore be regarded as descriptive and hypothesis-generating.

The cardiometabolic findings illustrate the importance of distinguishing prevalence from association. Hypertension, dyslipidemia, obesity, diabetes, and coronary artery disease were recurrently reported in both FFA and LPP, with hypertension affecting approximately one fifth of patients in both groups. However, a high prevalence within an affected population does not necessarily imply an increased risk attributable to the disease. Previous association-focused meta-analyses have yielded heterogeneous results. Phan and Smith found no significant association between LPP and dyslipidemia [24], while Chuang et al. reported no significant associations between LPP and hypertension, diabetes, hyperlipidemia, obesity, or heart failure [18]. These studies address a fundamentally different epidemiological question from the present analysis. Our findings establish the clinical frequency of cardiometabolic conditions within FFA and LPP populations but cannot determine whether their occurrence exceeds that expected on the basis of age, sex, or other population characteristics.

Thyroid disease was among the most consistently reported comorbidity domains, particularly in FFA, in which approximately one fifth of patients had hypothyroidism or an overall thyroid disorder. Thyroid abnormalities were also frequent in LPP. These findings are consistent with their recurrent reporting in previous clinical series and evidence syntheses [11,12], although association-focused analyses have not uniformly demonstrated an increased risk; for example, Phan and Smith did not identify a significant association between LPP and thyroid disease [17]. Differences between prevalence and association estimates may reflect not only methodological differences but also the demographic structure of the studied populations, particularly age and sex, as well as variation in diagnostic definitions and ascertainment. Selected subgroup differences remained significant after FDR correction, whereas no meta-regression association survived adjustment; these study-level findings remain exploratory.

Dermatological comorbidities further illustrate the heterogeneous clinical context of these disorders. Lichen planus and its variants were frequently reported in both conditions, consistent with their shared lichenoid background. In FFA, lichen planus pigmentosus, rosacea, atopic disorders, contact dermatitis, and vitiligo were also recurrently reported. Previous studies have described rosacea and contact dermatitis in FFA/LPP populations [12,25,26], prompting hypotheses involving barrier dysfunction, environmental exposures, cosmetic products, and cutaneous immune activation. Nevertheless, the present prevalence-based analysis cannot distinguish shared biological susceptibility from demographic effects, referral patterns, increased dermatological surveillance, or other sources of ascertainment bias.

Some included FFA cohorts contained patients with concomitant LPP, and other comorbidity estimates reflected the full FFA cohort as reported. The patients with concomitant LPP were not additionally entered as an independent LPP cohort. Their inclusion preserves the clinical composition of the original cohorts and reflects the coexistence encountered in clinical practice. However, aggregate data did not provide comorbidity counts separately for patients with and without concomitant LPP, preventing stratified estimation or adjustment. Differences in the inclusion and classification of these patients may have influenced the observed comorbidity profiles and should be considered when interpreting descriptive comparisons between FFA and LPP.

Biological observations provide context for these epidemiological patterns but should not be used to infer shared pathogenesis. Damage to the epithelial hair follicle stem-cell niche within the bulge is a central feature of primary cicatricial alopecia and contributes to irreversible follicular loss [27]. In FFA, transcriptomic studies have demonstrated prominent cutaneous Th1/JAK/STAT and fibrosis-related signatures, with limited evidence of corresponding systemic inflammatory activation [28], whereas IL-17-positive mast-cell infiltration and IL-23/IL-17 pathway activation have been described in LPP [9]. Activation of these pathways in affected skin does not, by itself, demonstrate systemic immune dysregulation. This meta-analysis synthesized comorbidity prevalence and did not evaluate molecular pathways or systemic immune activity. It therefore cannot establish shared pathogenic mechanisms between FFA/LPP and their comorbidities, and any proposed mechanistic links remain hypotheses requiring direct investigation.

The clinical implications should likewise be proportionate to the nature of the evidence. Our findings do not support universal laboratory screening for every reported comorbidity. Rather, the prevalence estimates provide a quantitative framework for targeted clinical assessment, particularly for frequently reported conditions such as thyroid and cardiometabolic disorders and for relevant dermatological and autoimmune comorbidities. Clinical evaluation and additional investigations should remain guided by symptoms, age, sex, medical and family history, disease phenotype, and planned treatment. The wide confidence intervals and substantial heterogeneity observed for several outcomes further argue against applying individual pooled estimates as universal screening thresholds.

This study has several strengths. It was prospectively registered and conducted using prevalence-specific methodological guidance, with separate quantitative syntheses for FFA and LPP, JBI critical appraisal, random-effects modeling, subgroup analyses, meta-regression with correction for multiple testing, and leave-one-out sensitivity analyses. The inclusion of 60 studies allowed a broad assessment across multiple comorbidity domains while maintaining a prevalence-focused sampling framework.

Several limitations should be considered. First, much of the evidence originated from retrospective, cross-sectional, or case-series data, and study populations differed substantially in demographic composition, referral setting, diagnostic criteria, and methods of comorbidity ascertainment. Second, several pooled estimates showed substantial between-study heterogeneity, and estimates based on small numbers of studies were sometimes sensitive to individual-study omission. Although exploratory subgroup analyses and meta-regression identified potential sources of heterogeneity, no meta-regression association remained significant after FDR correction, limiting causal or explanatory interpretation. Third, comorbidities were not systematically assessed in every study; selective reporting may therefore have influenced the apparent distribution of comorbidity domains. Fourth, the eligibility strategy may have increased the relative contribution of specialist or referral-based populations. Such settings may preferentially receive patients with diagnostically complex, persistent, or atypical disease, whose comorbidity profiles may differ from those of patients managed elsewhere. More frequent clinical assessments and targeted investigations may also increase the detection of concomitant dermatological or autoimmune conditions. These mechanisms could increase the observed frequency of some comorbidities, whereas conditions not routinely assessed may remain under-recorded; the direction of the resulting bias therefore need not be uniform across outcomes. Some excluded case–control studies could also have contributed informative prevalence data when their FFA/LPP groups were selected independently of the comorbidity under investigation and an appropriate denominator was available. Accordingly, the pooled estimates should be interpreted in relation to the clinical settings and ascertainment practices represented in the included studies, rather than assumed to apply equally across all FFA/LPP populations. The exclusion of case reports and case series with fewer than five patients has a different implication: it may reduce the breadth of rare comorbidities captured. Reports of unusual coexistence can identify conditions that are not routinely sought or documented in larger series. Their exclusion may therefore leave gaps in the description of the comorbidity spectrum, particularly for conditions reported only sporadically. However, reports selected because an unusual combination of diseases is present cannot establish how frequently that combination occurs in the wider FFA/LPP population. The minimum sample-size criterion applied to the study population, not to the number of comorbidity events; rare conditions could therefore still contribute when reported in eligible studies. This distinction is important because incomplete capture of rare conditions does not necessarily imply systematic underestimation of the pooled prevalence of the conditions that were analyzed. Absence from this synthesis should consequently be interpreted as a gap in the eligible evidence, rather than evidence that a particular comorbidity does not occur.

Fifth, our ability to assess small-study effects was limited because many comorbidities were reported in only two to nine studies. Egger’s test was therefore restricted to outcomes with at least ten contributing studies, and reporting bias could not be adequately assessed for the remaining outcomes. Even above this threshold, funnel plots and Egger’s test have limitations in prevalence meta-analyses, particularly for uncommon conditions. Differences in patient populations and in how comorbidities were assessed may cause smaller and larger studies to report different prevalences, without this necessarily reflecting publication bias. A significant test therefore does not establish publication bias, while a non-significant result cannot rule it out. These analyses should be interpreted cautiously, and uncertainty remains about the influence of unpublished studies and comorbidities that were assessed but not reported in the included publications.

In conclusion, FFA and LPP are accompanied by broad but heterogeneous patterns of reported systemic and dermatological comorbidity. Thyroid and cardiometabolic conditions, lichen-spectrum disorders, other hair disorders, and selected inflammatory or autoimmune diseases were recurrently identified, although their prevalence and consistency varied substantially across studies. These estimates provide a quantitative framework for clinical awareness and individualized assessment but should not be interpreted as evidence of excess risk or shared causality. Future prospective, population-based studies using standardized disease and comorbidity definitions, systematic ascertainment, and appropriate comparator populations are needed to distinguish disease-specific associations from demographic structure, referral patterns, surveillance effects, and shared biological susceptibility.

## Figures and Tables

**Table 1:** Pooled prevalence of comorbidities in lichen planopilaris. Random-effects meta-analysis of comorbidity prevalence in patients with lichen planopilaris (LPP). Results are presented as pooled prevalence with 95% confidence intervals (CIs). *k* denotes the number of contributing studies and I^2^ quantifies between-study heterogeneity.

Category	Comorbidity	*k* (Studies)	Events/Patients	Pooled Prevalence [95% CI]	I^2^ (%)
Atopy	Asthma	5	44/657	6.6% [3–13.8]	79
	Allergic rhinitis	3	29/444	6% [2.8–12.6]	65
	Atopic dermatitis	3	20/573	3.1% [1–8.6]	76
Cardiometabolic	Hypertension	7	124/635	21.2% [13.5–31.6]	83
	Dyslipidemia	7	104/674	16% [11.3–22.3]	65
	Obesity	5	47/461	12.7% [7.2–21.5]	72
	Diabetes (types 1 and 2)	4	23/356	6.8% [4.5–10.3]	9
	Coronary artery disease	3	21/322	6.8% [4.3–10.5]	3
Inflammatory dermatoses	Seborrheic dermatitis	5	159/694	13.9% [5.4–31.3]	95
	Rosacea	3	6/461	1.6% [0.8–3.5]	0
	Psoriasis	3	2/461	0.6% [0.2–1.9]	0
	Vitiligo	6	7/691	1.5% [0.6–3.4]	28
Lichen spectrum disorders	Lichen planus	6	68/622	11.1% [8–15.3]	40
	Lichen planus (cutaneous)	3	21/350	6.3% [4.1–9.5]	0
	Lichen planus (nail)	3	8/350	2.5% [1.2–4.9]	0
Mood and anxiety disorders	Depression	5	125/551	9.6% [2.3–32.2]	95
	Anxiety	4	111/480	9% [1.9–33.9]	94
Malignancies	Breast cancer	3	16/429	3.9% [2.4–6.3]	0
	Basal cell carcinoma	3	17/377	5.5% [2.2–13.2]	66
Systemic autoimmune diseases	Systemic lupus erythematosus	4	7/485	1.6% [0.8–3.4]	0
Thyroid diseases	Thyroid disorders	5	107/547	14.5% [7.4–26.3]	87
	Hypothyroidism	3	82/418	18.7% [12.2–27.5]	70
	Hashimoto disease	3	40/461	8% [3.3–18.1]	84
Other reported comorbidities	Vitamin D deficiency	3	52/350	18.3% [8.4–35.2]	88
	Gastroesophageal reflux disease	4	28/401	8.5% [4.3–16.1]	61
	Androgenetic alopecia	3	31/146	27.2% [10.3–55]	86
	Hepatitis C	3	4/350	1.4% [0.5–4.2]	15
	Migraine	3	14/382	3.9% [2.2–6.8]	5
	Chronic obstructive pulmonary disease	3	7/429	1.8% [0.8–3.7]	0

CI, confidence interval; LPP, lichen planopilaris. *k*, number of contributing studies. Pooled estimates for related comorbidities may derive from different subsets of studies and therefore should not be interpreted as nested proportions. Estimates based on few studies and/or showing substantial heterogeneity should be interpreted cautiously.

**Table 2:** Pooled prevalence of selected comorbidities in frontal fibrosing alopecia. Random-effects meta-analysis of comorbidity prevalence in frontal fibrosing alopecia (FFA). Results are presented as pooled prevalence (%) with 95% confidence intervals. Between-study heterogeneity is expressed as I^2^, and the number of contributing studies is indicated (*k*). Several pooled estimates showed substantial between-study heterogeneity (I^2^ > 75%) and should be interpreted cautiously. NA, not assessed because fewer than 10 studies contributed to the estimate.

Category	Comorbidity	*k* (Studies)	Events/Patients	Pooled Prevalence [95% CI]	I^2^ (%)	Egger *p* Value
Atopy	Atopy (general)	6	120/792	19.8% [7.1–44.3]	96	NA
	Atopic dermatitis	5	14/203	7% [4.2–11.5]	0	NA
	Contact dermatitis	3	9/153	7.1% [3.8–12.8]	0	NA
Cardiometabolic	Hypertension	22	368/1922	23.6% [17.8–30.6]	86	0.473
	Dyslipidemia	18	372/1785	17.9% [13.4–23.5]	82	0.09
	Diabetes (1 and 2)	7	42/697	9.2% [3.9–19.9]	84	NA
	Obesity	3	48/285	16.9% [12–23.3]	27	NA
	Hyperglycemia/Prediabetes	3	25/203	12.7% [8.7–18.1]	0	NA
	Coronary artery disease	3	34/560	6.2% [4.5–8.5]	0	NA
Inflammatory dermatoses	Psoriasis	16	41/1608	3.4% [2.1–5.4]	48	0.621
	Rosacea	15	163/1399	13.7% [8.2–22]	89	0.134
	Seborrheic dermatitis	6	40/423	8.2% [3.4–18.2]	76	NA
	Vitiligo	17	46/1925	3.3% [2.1–5]	44	0.697
Lichen spectrum disorders	Lichen planus	26	253/2508	10% [7.1–13.7]	78	0.246
	Lichen planus (cutaneous)	13	81/2340	4% [1.7–9.3]	91	0.508
	Lichen planus pigmentosus	11	100/499	19.6% [11.9–30.5]	81	0.151
	Lichen sclerosus	8	33/811	5.4% [2.6–11.2]	70	NA
	Lichen planus (oral)	8	35/696	5.2% [3–8.8]	45	NA
Malignancies	Breast cancer	5	18/276	6.9% [3–15.1]	60	NA
	Prostate cancer	3	8/58	14.1% [7.2–25.7]	0	NA
Mood and anxiety disorders	Depression	4	56/356	11.8% [3.6–32.4]	86	NA
	Anxiety	3	14/294	5% [1.6–14.5]	66	NA
Other hair disorders	Androgenetic alopecia	22	494/1524	31.9% [23.5–41.7]	90	0.426
	Alopecia areata	14	48/1941	3.1% [1.8–5.2]	66	0.085
	Coexisting classic LPP	9	107/830	13.1% [9.2–18.2]	62	NA
	Telogen effluvium	3	25/234	8.5% [1.4–38.4]	92	NA
Systemic autoimmune diseases	Autoimmune (overall)	9	344/1824	15.6% [9.1–25.4]	94	NA
	Systemic lupus erythematosus	8	24/636	4.4% [2.1–9]	67	NA
	Sjogren syndrome	6	12/895	1.9% [0.8–4.4]	45	NA
	Rheumatoid arthritis	5	15/734	4.5% [1.2–15.3]	84	NA
	Discoid lupus	4	9/237	4.3% [0.7–23.3]	84	NA
Thyroid diseases	Hypothyroidism	22	446/2357	20.6% [16.3–25.7]	83	0.994
	Thyroid disorders	17	454/1959	22.2% [15.5–30.9]	90	0.378
	Hashimoto disease	8	82/1013	9.1% [5.7–14.2]	72	NA
	Hyperthyroidism	3	14/562	2.6% [1.5–4.3]	0	NA
Other reported comorbidities	Ulcerative colitis	3	3/532	1.8% [0.2–14.1]	73	NA
	Pernicious anemia	3	10/874	1.2% [0.6–2.1]	0	NA
	Osteoporosis	5	69/678	11.4% [6.5–19.1]	76	NA
	Hyperparathyroidism	3	2/226	1.8% [0.4–7.9]	32	NA
	Celiac disease	4	15/1387	1.3% [0.6–2.8]	34	NA

CI, confidence interval; FFA, frontal fibrosing alopecia; LPP, lichen planopilaris. *k*, number of contributing studies. Egger’s regression test for funnel-plot asymmetry was performed only for outcomes with ≥10 contributing studies; NA indicates that the test was not performed. Pooled estimates for related or overlapping comorbidities may derive from different subsets of studies and should not be interpreted as nested proportions. Estimates based on few studies and/or showing substantial between-study heterogeneity should be interpreted cautiously.

## Data Availability

The extracted dataset is available in the Supplementary Materials. Analysis code is available from the corresponding author upon request.

## Supplementary Materials

The following supporting information can be downloaded at: https://www.mdpi.com/article/10.3390/jcm15197728/s1. Table S1: Search strategies used in electronic databases; Table S2: List of included studies in the systematic review; Table S3: List of excluded studies with reasons for exclusion; Table S4: Characteristics and comorbidity reporting of studies included in the systematic review of frontal fibrosing alopecia and lichen planopilaris; Table S5: Comorbidities reported in prevalence studies of patients with lichen planopilaris and frontal fibrosing alopecia; Table S6: Sex-stratified pooled prevalence of comorbidities in FFA and classic LPP; Table S7: Methodological quality assessment of included studies using the Joanna Briggs Institute checklist; Table S8: Summary of subgroup analyses exploring heterogeneity in comorbidities associated with FFA and LPP; Table S9: Univariable meta-regression analyses exploring sources of between-study heterogeneity; Table S10: Leave-one-out sensitivity analyses for pooled prevalence estimates in FFA and LPP; Table S11: PRISMA 2020 Checklist; Figure S1: Temporal evolution of included studies; Figure S2: Geographic distribution of included studies; Figure S3: Frequency of reported comorbidity domains; Figure S4: Exploratory indirect comparison of pooled prevalence estimates in frontal fibrosing alopecia and lichen planopilaris.
