# Supplementary material for: Comorbidity Burden in Frontal Fibrosing Alopecia and Lichen Planopilaris: A Systematic Review and Prevalence Meta-Analysis
Source: J Clin Med. 2026 Oct 7;15(19):7728. doi: 10.3390/jcm15197728 (PMC13666100; doi:10.3390/jcm15197728)
Supplement: Supplementary file 1 [file jcm-15-07728-s001.zip › jcm-4592439-supplementary.pdf]

SUPPLEMENTARY MATERIAL

Systematic Review

Title: Comorbidity burden in frontal fibrosing alopecia and lichen planopilaris: a systematic review and prevalence meta-analysis

Running title: Comorbidity burden in FFA and LPP

Keywords: Frontal fibrosing alopecia; Lichen planopilaris; Cicatricial alopecia; Comorbidities; Prevalence; Systematic review; Meta-analysis

Authors: Juan Luis Sanz-Cabanillas [1,2,*,†], Francisco José Gómez-García [1,2,†], Elena Beatriz Sanz-Cabanillas [2], Jesús Gay-Mimbrera [1], Antonio José Vélez García-Nieto [1,2,3], Beatriz Isla-Tejera [1,4,‡] and Juan Ruano [1,2,3,‡]

[1] Inflammatory Immune-Mediated Skin Diseases Laboratory (GC29), Maimonides Biomedical Research Institute of Córdoba (IMIBIC), University of Córdoba, 14004 Córdoba, Spain

[2] Department of Dermatology, Reina Sofía University Hospital, 14004 Córdoba, Spain

[3] Department of Medical and Surgical Sciences, Faculty of Medicine and Nursing, University of Córdoba, 14004 Córdoba, Spain

[4] Department of Pharmacy, Reina Sofía University Hospital, 14004 Córdoba, Spain

[†] These authors contributed equally and share first authorship.

[‡] These authors contributed equally and share senior authorship.

* **Corresponding author**:

Juan Luis Sanz-Cabanillas

Email: jsanzcab@gmail.com

SUPPLEMENTARY TABLES

Supplementary Table S1. Search strategies used in electronic databases

Databases: MEDLINE (via PubMed), EMBASE, ClinicalTrials.gov and preprints sources

Search period: From database inception to 18 June 2025

Last search update: 18 June 2025

Search strategy:

Concept 1 – Scarring alopecias and related disorders

(‘lichen planopilaris’ OR ‘frontal fibrosing alopecia’ OR ‘graham little syndrome’ OR ‘keratosis follicularis spinulosa decalvans’ OR ‘central centrifugal cicatricial alopecia’ OR ‘chronic cutaneous lupus erythematosus’ OR ‘discoid lupus erythematosus’ OR ‘folliculitis decalvans’ OR ‘dissecting folliculitis’ OR ‘cellulitis of the scalp’ OR ‘acne keloidalis’ OR ‘acne keloid’ OR ‘acne necrotica’ OR ‘erosive pustular dermatosis of the scalp’ OR ‘classical pseudopelade’ OR ‘brocq pseudopelade’ OR ‘follicular degeneration syndrome’ OR ‘alopecia mucinosa’ OR ‘follicular mucinosis’)

AND

Concept 2 – Metabolic, autoimmune, dermatological and systemic comorbidities

(‘metabolic syndrome’ OR ‘hyperlipidemia’ OR ‘hypertriglyceridemia’ OR ‘high-density lipoprotein cholesterol’ OR ‘vitamin d insufficiency’ OR ‘vitamin d deficiency’ OR ‘obesity’ OR ‘body mass index’ OR ‘diabetes mellitus type 2’ OR ‘diabetes mellitus type 1’ OR ‘diabetes overall’ OR ‘fasting blood glucose’ OR ‘insulin resistance’ OR ‘thyroid disease’ OR ‘coronary disease’ OR ‘cerebrovascular disease’ OR ‘hypertension’ OR ‘rosacea’ OR ‘seborrheic dermatitis’ OR ‘androgenetic alopecia’ OR ‘lichen planus pigmentosus’ OR ‘vitiligo’ OR ‘psoriasis’ OR ‘acne’ OR ‘atopy’ OR ‘asthma’ OR ‘allergic rhinitis’ OR ‘atopic dermatitis’ OR ‘lichen planus’ OR ‘hirsutism’ OR ‘osteopenia’ OR ‘osteoporosis’ OR ‘depression’ OR ‘systemic lupus erythematosus’ OR ‘inflammatory bowel disease’ OR ‘reproductive health’ OR ‘uterine fibroids’ OR ‘celiac disease’ OR ‘epilepsy’ OR ‘multiple sclerosis’ OR ‘crohn disease’ OR ‘ulcerative colitis’ OR ‘rheumatoid arthritis’ OR ‘psoriatic arthritis’ OR ‘sjogren’ OR ‘anemia’ OR ‘endometriosis’ OR ‘obstructive sleep apnea’ OR ‘lichen sclerosus’ OR ‘scleroderma’ OR ‘polycystic ovary syndrome’)

Limits: None

Language restrictions: None

Study design restrictions: None

Supplementary Table S2. List of included studies in the systematic review

ID	Article
**ID 1**	Oulad Ali S, Belcadi J, El Hilali S, Senouci K, Meziane M. Frontal fibrosing alopecia and comorbidities in a Moroccan population. JAAD Int. 2023;12:37-38. doi:10.1016/j.jdin.2023.04.003.
**ID 2**	Xavier de Brito FO, Cortez de Almeida RF, Machado CJ, Lemes LR, Donda ALV, Blanco A, et al. Frontal fibrosing alopecia associated with lichen planus pigmentosus: a multicentre retrospective descriptive analytical study of 104 patients. J Eur Acad Dermatol Venereol. 2023;37(8):e1033-e1037. doi:10.1111/jdv.19093.
**ID 3**	García A, Navarro MR, Ramirez A, Pino A, Navarro A, Moles I, et al. Plasma rich in growth factors as an adjuvant treatment for the management of frontal fibrosing alopecia: a retrospective observational clinical study. J Cutan Med Surg. 2023;27(4):340-349. doi:10.1177/12034754231177599.
**ID 4**	Moussa A, Bennett M, Bhoyrul B, Kazmi A, Asfour L, Sinclair RD. Clinical features and treatment outcomes of frontal fibrosing alopecia in men. Int J Dermatol. 2022;61:e372-e373.
**ID 5**	Salas-Callo CI, Tosti A, Stohmann D, Contarini P, Pirmez R. Eyelash involvement in frontal fibrosing alopecia: a prospective study. J Am Acad Dermatol. 2022;87(1):232-233. doi:10.1016/j.jaad.2021.07.062.
**ID 6**	Doche I, Nico MMS, Gerlero P, Rebeis M, Melo DF, Tortelly V, et al. Clinical features and sex hormone profile in male patients with frontal fibrosing alopecia: a multicenter retrospective study with 33 patients. J Am Acad Dermatol. 2022;86(5):1175-1177. doi:10.1016/j.jaad.2021.04.075.
**ID 7**	Lobato-Berezo A, Iglesias-Sancho M, Rodríguez-Lomba E, Mir-Bonafé JF, Velasco-Tamariz V, Porriño-Bustamante ML, et al. Frontal fibrosing alopecia in men: A multicenter study of 39 patients. J Am Acad Dermatol. 2022;86(2):481-484. doi:10.1016/j.jaad.2021.09.033.
**ID 8**	Rossi A, Tadiotto Cicogna G, Caro G, Fortuna MC, Magri F, Grassi S. Frontal fibrosing alopecia: a new association with lichen sclerosus in men. J Clin Aesthet Dermatol. 2021;14(9):54-58.
**ID 9**	Uzuncakmak TK, Ozkoca D, Askin O, Serdaroglu S. Evaluation of the demographic characteristics and the factors related to eyebrow involvement in frontal fibrosing alopecia: a retrospective, cross-sectional, single center study. Turkiye Klinikleri J Dermatol. 2021;31(2):93-99. doi:10.5336/dermato.2021-81322.
**ID 10**	Melo DF, Barreto TM, Saceda-Corralo D, Machado CJ, Brito FX, Tebet M, Tortelly VD, Moreno-Arrones OM, Vano-Galvan S. Epidemiologic and clinical features of pattern III frontal fibrosing alopecia (pseudo-fringe type): a multicenter series of 38 patients. J Am Acad Dermatol. 2021;84(3):797-799. doi:10.1016/j.jaad.2020.05.124.
**ID 11**	Grassi S, Tadiotto Cicogna G, Magri F, Fortuna MC, Caro G, Pernazza A, Soda G, Miraglia E, Giustini S, Carlesimo M, Rossi A. Frontal fibrosing alopecia and genital lichen sclerosus: single-center experience. J Cosmet Dermatol. 2021;20:615-620. doi:10.1111/jocd.13573.
**ID 12**	McSweeney SM, Christou EAA, Dand N, Boalch A, Holmes S, Harries M, et al. Frontal fibrosing alopecia: a descriptive cross-sectional study of 711 cases in female patients from the UK. Br J Dermatol. 2020;183:1136-1138. doi:10.1111/bjd.19399.

**ID 13**	Sari Aslani F, Saki N, Sasannia M. Frontal fibrosing alopecia: a clinicopathological study of 22 cases from Shiraz, Southern Iran. Iran J Dermatol. 2020;23:112-119.
**ID 14**	Maldonado Cid P, Leis Dosil VM, Garrido Gutierrez C, Salinas Moreno S, Thuissard Vasallo IJ, Andreu Vazquez C, Diaz Diaz RM. Frontal fibrosing alopecia: a retrospective study of 75 patients. Actas Dermosifiliogr. 2020;111(6):487-495. doi:10.1016/j.ad.2020.03.003.
**ID 15**	Kanti V, Constantinou A, Reygagne P, Vogt A, Kottner J, Blume-Peytavi U. Frontal fibrosing alopecia: demographic and clinical characteristics of 490 cases. J Eur Acad Dermatol Venereol. 2019;33(10):1976-1983.
**ID 16**	Kusano LDC, Mulinari-Brenner FA. Frontal fibrosing alopecia: follow-up of a Brazilian group. An Bras Dermatol. 2019;94(3):365-367. doi:10.1590/abd1806-4841.20197941.
**ID 17**	Valesky EM, Maier MD, Kaufmann R, Zoller N, Meissner M. Single-center analysis of patients with frontal fibrosing alopecia: evidence for hypothyroidism and a good quality of life. J Int Med Res. 2019;47(2):653-661. doi:10.1177/0300060518807335.
**ID 18**	Secchin P, Quintella DC, Paula NAO, Andrade LCS, Sodré CT. Clinical-histopathological profile of the frontal fibrosing alopecia: a retrospective study of 16 cases of a university hospital. An Bras Dermatol. 2019;94(4):416-421.
**ID 19**	Imhof RL, Chaudhry HM, Larkin SC, Torgerson RR, Tolkachjov SN. Frontal fibrosing alopecia in women: The Mayo Clinic experience with 148 patients, 1992-2016. Mayo Clin Proc. 2018;93(11):1581-1588. doi:10.1016/j.mayocp.2018.05.036.
**ID 20**	Pindado-Ortega C, Saceda-Corralo D, Buendía-Castaño D, Fernández-González P, Moreno-Arrones ÓM, Fonda-Pascual P, et al. Frontal fibrosing alopecia and cutaneous comorbidities: a potential relationship with rosacea. J Am Acad Dermatol. 2018;78(3):596-597.e1. doi:10.1016/j.jaad.2017.09.004.
**ID 21**	Zhang M, Zhang L, Rosman IS, Mann CM. Frontal fibrosing alopecia demographics: a survey of 29 patients. Cutis. 2019;103(2):E16-E22.
**ID 22**	Tolkachjov SN, Chaudhry HM, Camilleri MJ, Torgerson RR. Frontal fibrosing alopecia among men: a clinicopathologic study of 7 cases. J Am Acad Dermatol. 2017;77(4):683-690.e2.
**ID 23**	Banka N, Mubki T, Bunagan MJK, McElwee K, Shapiro J. Frontal fibrosing alopecia: a retrospective clinical review of 62 patients with treatment outcome and long-term follow-up. Int J Dermatol. 2014;53(11):1324-1330.
**ID 24**	Dlova NC. Frontal fibrosing alopecia and lichen planus pigmentosus: is there a link? Br J Dermatol. 2013;168(2):439-442. doi:10.1111/j.1365-2133.2012.11146.x.
**ID 25**	Conde Fernandes I, Selores M, Machado S. Frontal fibrosing alopecia: a review of eleven patients. Eur J Dermatol. 2011;21(5):750-752. doi:10.1684/ejd.2011.1419.
**ID 26**	Tan KT, Messenger AG. Frontal fibrosing alopecia: clinical presentations and prognosis. Br J Dermatol. 2009;160:75-79. doi:10.1111/j.1365-2133.2008.08861.x
**ID 27**	Vanó-Galván S, Molina-Ruiz AM, Serrano-Falcón C, et al. Frontal fibrosing alopecia: a multicenter review of 355 patients. J Am Acad Dermatol. 2014;70:670-678. doi:10.1016/j.jaad.2013.12.003

**ID 28**	Samrao A, Chew AL, Price V. Frontal fibrosing alopecia: a clinical review of 36 patients. Br J Dermatol. 2010;163:1296-1300. doi:10.1111/j.1365-2133.2010.09965.x
**ID 29**	Starace M, Brandi N, Alessandrini A, Bruni F, Piraccini BM. Frontal fibrosing alopecia: a case series of 65 patients seen in a single Italian centre. J Eur Acad Dermatol Venereol. 2019;33:433-438. doi:10.1111/jdv.15372
**ID 30**	Suchonwanit P, Pakornphadungsit K, Leerunyakul K, Khunkhet S, Sriphojanart T, Rojhirunsakool S. Frontal fibrosing alopecia in Asians: a retrospective clinical study. Int J Dermatol. 2020;59:184-190. doi:10.1111/ijd.14672
**ID 31**	Rocha VB, Machado CJ, Contin LA. Uncommon subtypes of frontal fibrosing alopecia: retrospective analysis of clinical characteristics and prognosis. An Bras Dermatol. 2022;97(2):260-262. doi:10.1016/j.abd.2021.02.009
**ID 32**	MacDonald A, Clark C, Holmes S. Frontal fibrosing alopecia: a review of 60 cases. J Am Acad Dermatol. 2012;67(5):955-961. doi:10.1016/j.jaad.2011.12.038
**ID 33**	Rayinda T, McSweeney SM, Dand N, Fenton DA, McGrath JA, Tziotzios C. Clinical characteristics of male frontal fibrosing alopecia: a single-centre case series from London, UK. Br J Dermatol. 2022;186:195-196. doi:10.1111/bjd.20742
**ID 34**	Alegre-Sánchez A, Saceda-Corralo D, Bernárdez C, Molina-Ruiz AM, Arias-Santiago S, Vañó-Galván S. Frontal fibrosing alopecia in male patients: a report of 12 cases. J Eur Acad Dermatol Venereol. 2017;31:e112-e114. doi:10.1111/jdv.13855
**ID 35**	Ladizinski B, Bazakas A, Selim MA, Olsen EA. Frontal fibrosing alopecia: a retrospective review of 19 patients seen at Duke University. J Am Acad Dermatol. 2013;68(5):749-755. doi:10.1016/j.jaad.2012.09.043
**ID 36**	Strazzulla LC, Avila L, Li X, Lo Sicco K, Shapiro J. Prognosis, treatment, and disease outcomes in frontal fibrosing alopecia: a retrospective review of 92 cases. J Am Acad Dermatol. 2018;78(1):203-204. doi:10.1016/j.jaad.2017.07.052
**ID 37**	Ormaechea-Pérez N, López-Pestana A, Zubizarreta-Salvador J, Jaka-Moreno A, Panés-Rodríguez A, Tuneu-Valls A. Alopecia frontal fibrosante en el varón: presentación de 12 casos y revisión de la literatura. Actas Dermosifiliogr. 2016;107(10):836-844.
**ID 38**	Pindado-Ortega C, Saceda-Corralo D, Moreno-Arrones OM, Rodrigues-Barata AR, Hermosa-Gelbard A, Jaén-Olasolo P, Vañó-Galván S. Effectiveness of dutasteride in a large series of patients with frontal fibrosing alopecia in real clinical practice. J Am Acad Dermatol. 2021;84(5):1285-1294. doi:10.1016/j.jaad.2020.09.093
**ID 39**	Heppt MV, Letulé V, Laniauskaite I, Reinholz M, Tietze JK, Wolff H, Ruzicka T, Sattler EC. Frontal fibrosing alopecia: a retrospective analysis of 72 patients from a German academic center. Facial Plast Surg. 2018;34(1):88-94. doi:10.1055/s-0037-1615281
**ID 40**	Carmona-Rodríguez M, Moro-Bolado F, Romero-Aguilera G, Ruiz-Villaverde R, Carriel V. Frontal fibrosing alopecia: an observational single-center study of 306 cases. Life (Basel). 2023;13:1344. doi:10.3390/life13061344.
**ID 41**	Starace M, Orlando G, Iorizzo M, Alessandrini A, Bruni F, Mandel VD, et al. Clinical and dermoscopic approaches to diagnosis of frontal fibrosing alopecia: results from a multicenter study of the International Dermoscopy Society. Dermatol Pract Concept. 2022;12(1):e2022080. doi:10.5826/dpc.1201a80.
**ID 42**	Adotama P, Callender V, Kolla A, Young C, Jones P, Svigos K, et al. Comparing the clinical differences in white and black women with frontal

	fibrosing alopecia. Br J Dermatol. 2021;185(5):1074-1075. doi:10.1111/bjd.20605.
**ID 43**	Saceda-Corralo D, Ortega-Quijano D, Muñoz-Martín G, Moreno-Arrones OM, Pindado-Ortega C, Rayinda T, et al. Genotyping of the rs1800440 polymorphism in CYP1B1 gene and the rs9258883 polymorphism in HLA-B gene in a Spanish cohort of 223 patients with frontal fibrosing alopecia. Acta Derm Venereol. 2023;103:adv9604. doi:10.2340/actadv.v103.9604.
**ID 44**	Dorgham NA, Hegazy R, Farag A, Dorgham DA. Frontal fibrosing alopecia: a retrospective clinical review of 58 Egyptian patients with treatment outcome and long-term follow-up. J Eur Acad Dermatol Venereol. 2022;36(3):e212-e213. doi:10.1111/jdv.17747.
**ID 45**	Verma S, Marak A, Paul D, Dey B. A retrospective study of frontal fibrosing alopecia from North-East India. Indian J Dermatol. 2023;68:598-602. doi:10.4103/ijd.IJD_290_23.
**ID 46**	Gkini MA, Riaz R, Jolliffe V. A retrospective analysis of efficacy and safety of intralesional triamcinolone injections in the treatment of frontal fibrosing alopecia either as monotherapy or adjuvant therapy. Int J Trichology. 2018;10:162-168. doi:10.4103/ijt.ijt_46_18.
**ID 47**	Bala L, Golahmadi AK, Elshimy N. Central centrifugal cicatricial alopecia and fibroproliferative disorders: exploring the association with uterine leiomyomas. Clin Exp Dermatol. 2025;50:2498-2500. doi:10.1093/ced/llaf339.
**ID 48**	Conic RRZ, Piliang M, Bergfeld W, Atanaskova-Mesinkovska N. Vitamin D status in scarring and nonscarring alopecia. J Am Acad Dermatol. 2021;85(2):478-480. doi:10.1016/j.jaad.2018.04.032.
**ID 49**	Lyakhovitsky A, Zilbermintz T, Segal Z, Galili E, Shemer A, Jaworowski B, Baum S, Hermush V, Kaplan B, Barzilai A. Exploring remission dynamics and prognostic factors in lichen planopilaris: a retrospective cohort study. Dermatology. 2024;240:531-542. doi:10.1159/000538355.
**ID 50**	Oak ASW, Yang K, Reddy SB, Elston CA, Mayo TT. Lichen planopilaris in 24 African American women. Int J Womens Dermatol. 2024;10:e141. doi:10.1097/JW9.0000000000000141.
**ID 51**	Gharaei Nejad K, Ghadarjani R, Eftekhari H, Sheykholeslami S. Most frequent comorbidities in patients with lichen planopilaris: a cross-sectional study. Int J Dermatol Venereol. 2023;6(4):229-232. doi:10.1097/JD9.0000000000000306.
**ID 52**	Cantwell HM, Wieland CN, Proffer SL, Imhof RL, Torgerson RR, Tolkachjov SN. Lichen planopilaris in men: a retrospective clinicopathologic study of 19 patients. Int J Dermatol. 2021;60:482-488. doi:10.1111/ijd.15285.
**ID 53**	Larkin SC, Cantwell HM, Imhof RL, Torgerson RR, Tolkachjov SN. Lichen planopilaris in women: a retrospective review of 232 women seen at Mayo Clinic from 1992 to 2016. Mayo Clin Proc. 2020;95(8):1684-1695. doi:10.1016/j.mayocp.2020.02.028.
**ID 54**	Chaudhry H, Tolkachjov S, Camilleri M, Torgerson R. A clinicopathologic study of lichen planopilaris among men. J Am Acad Dermatol. 2017;76(6 Suppl 1):AB11.
**ID 55**	Ratnaparkhi R, Tellez A, Piliang M, Bergfeld W. Association of lichen planopilaris with seborrheic dermatitis: a retrospective case-control study. J Am Acad Dermatol. 2016;74(5 Suppl 1):AB133.
**ID 56**	Kerkemeyer KLS, Green J. Lichen planopilaris: a retrospective study of 32 cases in an Australian tertiary referral hair clinic. Australas J Dermatol. 2018;59(4):297-301. doi:10.1111/ajd.12772.

**ID 57**	Carrascoza GG, Macedo T, Romiti R, Doche I, Rivitti-Machado MCM. Lichen planopilaris and frontal fibrosing alopecia-associated conditions: a retrospective study with 241 patients. J Eur Acad Dermatol Venereol. 2024;38:e827-e829. doi:10.1111/jdv.19891.
**ID 58**	Pathoulas JT, Flanagan KE, Walker CJ, Collins MS, Ali S, Pupo Wiss IM, et al. A multicenter descriptive analysis of 270 men with frontal fibrosing alopecia and lichen planopilaris in the United States. J Am Acad Dermatol. 2023;88(4):937-939. doi:10.1016/j.jaad.2022.10.060.
**ID 59**	Mardones F, Shapiro J. Lichen planopilaris in a Latin American (Chilean) population: demographics, clinical profile and treatment experience. Clin Exp Dermatol. 2017;42(7):755-759. doi:10.1111/ced.13203.
**ID 60**	Meinhard J, Stroux A, Lünnemann L, Vogt A, Blume-Peytavi U. Lichen planopilaris: epidemiology and prevalence of subtypes – a retrospective analysis in 104 patients. J Dtsch Dermatol Ges. 2014;12(3):229-235. doi:10.1111/ddg.12264.

Study IDs retain their original numbering.

Supplementary Table S3. List of excluded studies with reasons for exclusion

ID	Article	Reason for exclusion
1	Starace MVR, Pampaloni F, Iorizzo M, Apalla Z, Asfour L, Freites-Martinez A, et al. Delphi Consensus on the Distinct Clinical and Histopathological Features of Lichen Planopilaris and Frontal Fibrosing Alopecia: Insights From the Hair Diseases EADV Task Force. Int J Dermatol. 2025;64:1246-1254. doi:10.1111/ijd.17780.	Literature review
2	Willaert M, van Dongen TH, Dikrama P, Nijsten TEC, Hijnen DJ, Waalboer-Spuij R. Systemic Treatments for Lichen Planopilaris and Frontal Fibrosing Alopecia: A Retrospective Study of 315 Patients. Acta Derm Venereol. 2025;105:adv42465. doi:10.2340/actadv.v105.42465.	Did not assess comorbidities
3	Verma S, Marak A, Paul D. Frontal fibrosing alopecia: a comprehensive review with recent updates. Indian J Dermatol. 2025;70:115. doi:10.4103/ijd.ijd_419_24.	Literature review
4	Folliat A, Brenaut E, Misery L, Abasq-Thomas C. Characteristics of pruritus in lichen planopilaris and frontal fibrosing alopecia: A cohort study in a French hospital. J Am Acad Dermatol. 2024;91(5):1015-1016. doi:10.1016/j.jaad.2024.07.1476.	Did not assess comorbidities
5	Lam W, Tosti A. Pediatric lichen planopilaris: a review [Abstract 53519]. J Am Acad Dermatol. 2024;91(3 Suppl):AB70.	Literature review
6	Whiting C, Abdel Azim S, Zahn J, Friedman A. Highlighting the link between lichen planus pigmentosus and frontal fibrosing alopecia: a case series of 3 patients [Abstract 52986]. J Am Acad Dermatol. 2024;91(3 Suppl):AB226.	Did not assess comorbidities.
7	Fenner B, Verma K, Pham M, Tarbox M. Common contact allergens implicated in frontal fibrosing alopecia found in over-the-counter hair growth serums and solutions [Abstract 53607]. J Am Acad Dermatol. 2024;91(3 Suppl):AB163. doi:10.1016/j.jaad.2024.07.648.	Did not assess comorbidities
8	Pelet Del Toro N, Strunk A, Garg A, Han G. Prevalence and Treatment Patterns in Patients With Lichen Planopilaris. JAMA Dermatol. 2024;160(8):864-868. doi:10.1001/jamadermatol.2024.1445.	Did not assess comorbidities
9	Anzai A, Abdo Abujamra B, Aoki V, Jozic I. Upregulation of caveolae-associated structural proteins in the hair follicle bulge of lichen planopilaris and frontal fibrosing alopecia. Arch Dermatol Res. 2024;316:330. doi:10.1007/s00403-024-03153-3.	Did not assess comorbidities
10	Joshi TP, Zhu H, Naqvi Z, Holla S, Duruewuru A, Ren V. Prevalence of lichen planopilaris and frontal fibrosing alopecia in the United States: an All of Us database analysis. Int J Dermatol. 2024;63:1099-1101. doi:10.1111/ijd.17274.	Did not assess comorbidities
11	Maazi M, Savinova I, McMullen E, Donovan J. Exploring the relationship between lichen planopilaris and skin cancer. Int J Dermatol. 2025;64:1293-1294. doi:10.1111/ijd.17578.	Literature review
12	Otlewska-Szpotowicz A, Baran W, Woźniak Z, Szepietowski J, Batycka-Baran A. Increased expression of psoriasin (S100A7) and interleukin 17 (IL-17) in lesional skin in lichen planopilaris. Adv Dermatol Allergol. 2024;41(4):364-371. doi:10.5114/ada.2024.142179.	Did not assess comorbidities

ID	Article	Reason for exclusion
**13**	Desai S, Nussbaum D, Friedman A. 44511 Double trouble: Graham-Little-Piccardi-Lasseur syndrome and hidradenitis suppurativa, is there a link? J Am Acad Dermatol. 2023;89(3 Suppl):AB39. doi:10.1016/j.jaad.2023.07.159.	Case report
**14**	Christensen KN, Lehman JS, Tollefson MM. Pediatric lichen planopilaris: clinicopathologic study of four new cases and a review of the literature. Pediatr Dermatol. 2015;32(5):621-627. doi:10.1111/pde.12624.	Literature review
**15**	Tam M, Nixon R. Patch testing and frontal fibrosing alopecia: 20 year data from tertiary patch testing clinic in Melbourne. Australas J Dermatol. 2023;64(Suppl 1):38. doi:10.1111/ajd.14039.	Did not assess comorbidities
**16**	Lobato-Berezo A, Gonzalez-Farré M, Marcantonio O, Pujol RM. Dystrophic xanthomization secondary to lichen planus pigmentosus associated with frontal fibrosing alopecia. J Cosmet Dermatol. 2022;21:7251-7252. doi:10.1111/jocd.15444.	Case report
**17**	Varghaei A, Rostami A, Yarmohamadi M, Mahmoudi H, Balighi K, Daneshpazhooh M. Assessment of health-related quality of life in patients with frontal fibrosing alopecia. J Cosmet Dermatol. 2022;21:6169-6173. doi:10.1111/jocd.15183.	Did not assess comorbidities
**18**	Jacob MA, Jain D, Shaw R. BH09: Frontal fibrosing alopecia in men: is it any different? Br J Dermatol. 2022;187(S1):100. doi:10.1111/bjd.21337.	Case report
**19**	Gatica-Ortega ME, Pastor-Nieto MA, Torres-Aranda R, Alonso-Naranjo L, Pérez-Hortet C. Contact sensitization to Magnolia officinalis bark extract and other allergens in a patient with frontal fibrosing alopecia and lichen planus pigmentosus. Contact Dermatitis. 2022;86(5):434-436. doi:10.1111/cod.14053.	Case report
**20**	Griggs J, Trüeb RM, Reis Gavazzoni Dias MF, Hordinsky M, Tosti A. Fibrosing alopecia in a pattern distribution. J Am Acad Dermatol. 2021;85:1557-1564. doi:10.1016/j.jaad.2019.12.056.	Literature review
**21**	Izadi Firouzabadi L, Stonard C, Perkins W. Red eyebrows as the only manifestation of frontal fibrosing alopecia. Br J Dermatol. 2021;184:e161.	Case report
**22**	Ocampo-Garza SS, Orizaga-y-Quiroga TL, Olvera-Rodríguez V, Herz-Ruelas ME, Chavez-Alvarez S, Vañó-Galván S, Ocampo-Candiani J. Frontal fibrosing alopecia: is there a link in relatives? Skin Appendage Disord. 2021;7:206-211. doi:10.1159/000512039.	Case report
**23**	Swamy MR, Kam S, Kopelman H, Goldberg LJ. Frontal fibrosing alopecia in premenopausal women: a case report. Int J Womens Dermatol. 2020;6:229. doi:10.1016/j.ijwd.2020.03.018.	Case report
**24**	Zinoune S, Baybay H, Kadiri S, Elloudi S, Douhi Z, Mernissi FZ. Frontal fibrosing alopecia and concomitant lichen planus pigmentosus: a series of 17 cases. In Proceedings of the 24th World Congress of Dermatology, Milan, Italy, 10-15 June 2019 [Conference abstract]. Available online: https://www.wcd2019milan-dl.org/abstract-book/documents/abstracts/18-hair-disorders/frontal-fibrosing-alopecia-and-concomitant-6383.pdf (accessed on 6 October 2026).	Did not assess comorbidities
**25**	Chhabra G, Verma P. A singular association of vitiligo, psoriasis and frontal fibrosing alopecia. Australas J Dermatol. 2019;60:e127-e128. doi:10.1111/ajd.13164.	Case report

ID	Article	Reason for exclusion
**26**	Micheletti L, Haefner H, Zalewski K, MacLean A, Gomez Cherey F, Pereira C, Sluga C, Solé-Sedeno JM, Vargas-Hernandez VM, Preti M. Surgical terminology for vulvar cancer treatment. J Low Genit Tract Dis. 2019;23(4 Suppl 1):S37-S52.	Did not assess comorbidities
**27**	Porriño-Bustamante ML, López-Nevot MÁ, Aneiros-Fernández J, García-Lora E, Fernández-Pugnaire MA, Arias-Santiago S. Familial frontal fibrosing alopecia: a cross-sectional study of 20 cases from nine families. Australas J Dermatol. 2019;60:e113-e118. doi:10.1111/ajd.12951.	Did not assess comorbidities
**28**	Kłosowicz A, Englert K, Pełka K, Pastuszczak M, Pastuszczak A. Autoimmunity in lichen planopilaris patients. Pol Merkuriusz Lek. 2019;46(271):32-35. PMID:30810113.	Did not assess comorbidities
**29**	Brankov N, Conic RZ, Atanaskova-Mesinkovska N, Piliang M, Bergfeld WF. Comorbid conditions in lichen planopilaris: a retrospective data analysis of 334 patients. Int J Womens Dermatol. 2018;4:180-184. doi:10.1016/j.ijwd.2018.04.001.	Duplicated
**30**	Correction: Frontal fibrosing alopecia and cutaneous comorbidities: a potential relationship with rosacea. J Am Acad Dermatol. 2018;79(2):406. doi:10.1016/j.jaad.2018.02.029.	Did not assess comorbidities
**31**	Saceda-Corralo D, Pindado-Ortega C, Moreno-Arrones OM, Fernández-González P, Rodrigues-Barata AR, Jaén-Olasolo P, et al. Health-related quality of life in patients with frontal fibrosing alopecia. JAMA Dermatol. 2018;154(4):479-480. doi:10.1001/jamadermatol.2017.6330.	Did not assess comorbidities
**32**	Petrof G, Cuell A, Rajkomar VV, Harries MJ, Takwale A, Holmes S, et al. Retrospective review of 18 British South Asian women with frontal fibrosing alopecia. Int J Dermatol. 2018;57:490-491.	Did not assess comorbidities
**33**	Romiti R, Gavioli CFB, Anzai A, Munck A, Fechine COC, Valente NYS. Clinical and histopathological findings of frontal fibrosing alopecia-associated lichen planus pigmentosus. Skin Appendage Disord. 2017;3(2):59-63. doi:10.1159/000456038.	Did not assess comorbidities
**34**	Kelati A, Jafferany M. Quality-of-life assessment and the impact of early diagnosis and treatment in lichen planopilaris: a systematic review. Clin Exp Dermatol. 2025;50:146-149. doi:10.1093/ced/llae339.	Systematic review
**35**	Donati A, Hue Wu II. Extra-follicular cutaneous manifestations of frontal fibrosing alopecia. An Bras Dermatol. 2024;99(6):875-886. doi:10.1016/j.abd.2024.01.003.	Literature review
**36**	George SE, Rodriguez I, Adler BL, Yu J. Tangled truths: unraveling the link between frontal fibrosing alopecia and allergic contact dermatitis. Cutis. 2024;113(3):119-122. doi:10.12788/cutis.0974.	Literature review
**37**	Roman AM, Petca RC, Dumitrascu MC, Petca A, Ionescu AI, Sandru F. Frontal fibrosing alopecia and reproductive health: assessing the role of sex hormones in disease development. J Pers Med. 2024;14(1):72. doi:10.3390/jpm14010072.	Literature review
**38**	Damke JP, Schoenardie BO, Maciel RF, Peruzzo J. Frontal fibrosing alopecia coexisting with vitiligo: is there a true association? An Bras Dermatol. 2023;98(6):853-855. doi:10.1016/j.abd.2021.10.018.	Case report
**39**	Kothari R, Vashisht D, Venugopal R, Paliwal G, Pathania V, Radhakrishnan S. Co-existing alopecia areata, lichen planus, and lichen	Case report

ID	Article	Reason for exclusion
	planopilaris: a rare occurrence. Indian Dermatol Online J. 2023;14:726-728. doi:10.4103/idoj.idoj_623_22.	
**40**	Gavazzoni Dias MFR, Bunagan MJK, Tosti A. Case Series of Frontal Fibrosing Alopecia and Fibrosing Alopecia in a Pattern Distribution: Is There a Familial Correlation? Skin Appendage Disord. 2023;9:230-234. doi:10.1159/000529923.	Did not assess comorbidities
**41**	Boudreaux C, Boulinguez S, Beylot-Barry M, Chosidow O, Vignon-Pennamen MD, Raux G, et al. Basal cell carcinoma arising within an alopecic patch of lichen planopilaris. JAAD Case Rep. 2023;32:103-105. doi:10.1016/j.jdcr.2022.12.017.	Case report
**42**	Trujillo-Gaviria L, Gómez-Osorio D, Ruíz-Restrepo JD, Valencia-Ocampo OJ. Coexistence of vitiligo and frontal fibrosing alopecia: a case report and analysis of its physiopathology. Actas Dermosifiliogr. 2022;113:T991-T994. doi:10.1016/j.ad.2022.09.015.	Case report
**43**	Gatica-Ortega ME, Vergara-de-la-Campa L, Alonso-Naranjo L, Pastor-Nieto MA. Relevant sensitization to diethylamino hydroxybenzoyl hexyl benzoate and fragrances in a patient with frontal fibrosing alopecia and acquired dermal macular hyperpigmentation. Contact Dermatitis. 2022;87(3):287-289. doi:10.1111/cod.14139.	Case report
**44**	Liu J, Ko CJ, Azar A, Glicksberg BS, Mesinkovska NA, Kahlenberg JM. Association between frontal fibrosing alopecia and rosacea: results from clinical observational studies and gene expression profiles. Front Immunol. 2022;13:985081. doi:10.3389/fimmu.2022.985081.	Systematic review
**45**	Bui K, Huynh W, Jialal I. Multiple clinical manifestations of lichenoid spectrum: a patient with frontal fibrosing alopecia, lichen planus pigmentosus, and nail lichen planus. Skin Appendage Disord. 2022;8:225-228. doi:10.1159/000523590.	Case report
**46**	Bodnariuk N, Bastard DP, Enz PA. Frontal fibrosing alopecia and lichen planus pigmentosus: variants of the same disease? Actas Dermosifiliogr. 2022;113:617-618. doi:10.1016/j.ad.2020.08.028.	Case report
**47**	Gambichler T, Skrygan M, Tomi NS, et al. Examining the autoimmune aspects of frontal fibrosing alopecia. J Eur Acad Dermatol Venereol. 2020;34(9):e477-e479. doi:10.1111/jdv.16386.	Literature review
**48**	Lis-Święty A, Brzezińska-Wcisło L. Potential etiological factors in frontal fibrosing alopecia. Postepy Dermatol Alergol. 2020;37(4):482-489. doi:10.5114/ada.2020.98252.	Literature review
**49**	Kerkemeyer KL, Eisman S, Bhoyrul B, Pinczewski J, Sinclair R. Frontal fibrosing alopecia. Clin Dermatol. 2021;39:183-193. doi:10.1016/j.clindermatol.2020.10.007.	Literature review
**50**	Verzì AE, Lacarrubba F, Dall'Oglio F, Micali G. Association of frontal fibrosing alopecia with facial papules and lichen planus pigmentosus in a Caucasian woman. Skin Appendage Disord. 2020;6:379-383. doi:10.1159/000509407.	Case report
**51**	Martinez-Rico JC, Ancer-Arellano J, Martinez-Moreno A, Ocampo-Garza SS, Ocampo-Candiani J, Chavez-Alvarez S. Frontal fibrosing alopecia and autoimmune disorders in a Hispanic female. Skin Appendage Disord. 2020;6:362-365. doi:10.1159/000507557.	Case report
**52**	Phan K, Smith SD. Lichen planopilaris and dyslipidaemia: systematic review and meta-analysis. Clin Exp Dermatol. 2020;45:611-613. doi:10.1111/ced.14190.	Systematic review

ID	Article	Reason for exclusion
**53**	Beyzaee AM, Babaei M, Ghoreishi B, Goldust M, Rahmatpour Rokni G. Association between allergic contact dermatitis and frontal fibrosing alopecia: a narrative review. Iran J Dermatol. 2025;28(1):42-45. doi:10.22034/ijd.2024.493229.1942.	Literature review
**54**	Wu CY, Liu CY, Liu YCS. Frontal fibrosing alopecia developing on vitiligo. Indian J Dermatol Venereol Leprol. 2019;85(5):514-517.	Case report
**55**	Genovese G, Muratori S, Fanoni D, Venegoni L, Zussino M, Berti E. Lichen planopilaris and dermatitis herpetiformis: a fortuitous association? Eur J Dermatol. 2019;29(4):426-428. doi:10.1684/ejd.2019.3570.	Case report
**56**	Phan K, Smith SD. Lichen planopilaris and thyroid disease: systematic review and meta-analysis. Ann Thyroid. 2020;5:4. doi:10.21037/aot.2020.02.02.	Systematic review
**57**	Abdalla BMZ, Matsunaga N, Landman G, Machado Filho CD, Criado PR. Association between frontal fibrosing alopecia and linear scleroderma “coup de sabre”. Australas J Dermatol. 2019;60:e256-e257. doi:10.1111/ajd.12979.	Case report
**58**	Rosina P, Chieregato C, Magnanini M, Barba A. Lichen planopilaris and autoimmune thyroiditis. J Eur Acad Dermatol Venereol. 2002;16:648-649.	Case report
**59**	Larkin SC, Cantwell HM, Imhof RL, Torgerson RR, Tolkachjov SN. Lichen planopilaris in women: A retrospective review of 232 women seen at Mayo Clinic from 1992 to 2016. Mayo Clin Proc. 2020;95(8):1684-1695. doi:10.1016/j.mayocp.2020.02.028.	Duplicated
**60**	Alirezaei P, Ahmadpanah M, Rezanejad A, Soltanian A, Sadeghi Bahmani D, Brand S. Compared to controls, individuals with lichen planopilaris have more depression, a lower self-esteem, and a lower quality of life. Neuropsychobiology. 2019;78:95-103. doi:10.1159/000499135.	Did not assess comorbidities
**61**	Uwakwe LN, Cardwell LA, Dothard EH, Baroudi BI, McMichael AJ. Frontal fibrosing alopecia and concomitant lichen planus pigmentosus: A case series of seven African American women. J Drugs Dermatol. 2018;17(4):397-400.	Did not assess comorbidities
**62**	Porrino-Bustamante ML, Aneiros-Fernández J, Arias-Santiago S. Frontal fibrosing alopecia and rosacea: Is there any link? J Am Acad Dermatol. 2017;76(6 Suppl 1):AB142.	Duplicated
**63**	Ranasinghe GC, Piliang MP, Bergfeld WF. Prevalence of hormonal and endocrine dysfunction in patients with lichen planopilaris (LPP): A retrospective data analysis of 168 patients. J Am Acad Dermatol. 2017;76:314-320.	Duplicated
**64**	del Rei M, Pirmez R, Sodré CT, Tosti A. Coexistence of frontal fibrosing alopecia and discoid lupus erythematosus of the scalp in 7 patients: just a coincidence? J Eur Acad Dermatol Venereol. 2016 Jan;30(1):151-153. doi:10.1111/jdv.12642.	Duplicated
**65**	Shah V, Reisz C. Frontal fibrosing alopecia: Iatrogenic endocrine disruption. J Am Acad Dermatol. 2015 May;72(5 Suppl 1):AB119. doi:10.1016/j.jaad.2015.02.492.	Did not assess comorbidities
**66**	Miteva M, Aber C, Torres F, Tosti A. Frontal fibrosing alopecia occurring on scalp vitiligo: report of four cases. Br J Dermatol. 2011;165:445-447.	Did not assess comorbidities

ID	Article	Reason for exclusion
**67**	Nasimi M, Lajevardi V, Mahmoudi H, Arbab F, Shakoei S. Metabolic syndrome in patients with oral lichen planus and lichen planopilaris: a cross-sectional study. Iran J Dermatol. 2021;24:186-192. doi:10.22034/ijd.2020.229042.1082.	Duplicated
**68**	Cevasco NC, Bergfeld WF, Remzi BK, Ramirez de Knott H. A case series of 29 patients with lichen planopilaris: the Cleveland Clinic Foundation experience on evaluation, diagnosis, and treatment. J Am Acad Dermatol. 2007;57(1):47-53. doi:10.1016/j.jaad.2007.01.011.	Duplicated
**69**	Moreno-Ramírez D, Camacho Martínez F. Frontal fibrosing alopecia: a survey in 16 patients. J Eur Acad Dermatol Venereol. 2005;19:700-705. doi:10.1111/j.1468-3083.2005.01291.x.	Did not assess comorbidities
**70**	Chuang KW, Huang SW, Chang HC. Association of lichen planopilaris with cardiovascular and metabolic disorders: a systematic review and meta-analysis. Clin Exp Dermatol. 2025;50:537-543. doi:10.1093/ced/llae446.	Systematic review
**71**	del Rei M, Pirmez R, Sodré CT, Tosti A. Coexistence of frontal fibrosing alopecia and discoid lupus erythematosus of the scalp in 7 patients: just a coincidence? J Eur Acad Dermatol Venereol. 2016;30:151-153. doi:10.1111/jdv.12642.	Did not assess comorbidities
**72**	Balazic E, Konisky H, Axler E, Kobets K. Concurrent lichen planopilaris and female androgenic alopecia in skin of color: a case series. J Drugs Dermatol. 2025;24(3):320-322. doi:10.36849/JDD.7318.	Did not assess comorbidities
**73**	Alenezi S, Ezzat R, Miteva M. Frontal fibrosing alopecia (FFA) Part I - diagnosis and clinical presentation. J Am Acad Dermatol. 2025. doi:10.1016/j.jaad.2024.10.126.	Literature review
**74**	Ezzat RZ, Alenezi S, Miteva M. Frontal fibrosing alopecia Part II: etiopathogenesis and management. J Am Acad Dermatol. 2026;94:1075-1085. doi:10.1016/j.jaad.2024.08.086.	Literature review
**75**	Mehregan DA, Van Hale HM, Muller SA. Lichen planopilaris: clinical and pathologic study of forty-five patients. J Am Acad Dermatol. 1992;27:935-942.	Did not assess comorbidities
**76**	Papierzewska M, Waśkiel-Burnat A, Rudnicka L. Lichen planopilaris in children: a systematic review. Pediatr Dermatol. 2025;42:22-30. doi:10.1111/pde.15835.	Systematic review
**77**	Boga S, Acosta A, Alvarez M. Liquen plano pilaris en edad pediátrica: presentación de 4 casos clínicos y revisión de la literatura. Piel (Barc). 2025;40(5):264-269.	Did not assess comorbidities
**78**	Nutz MC, Deussing M, Hartmann D, Lange S, Senner S, Eyssele T, et al. Line-field confocal optical coherence tomography in lichen planopilaris and frontal fibrosing alopecia: a pilot study. J Dtsch Dermatol Ges. 2025;23(2):173-181. doi:10.1111/ddg.15591.	Did not assess comorbidities
**79**	Donovan J. The frontal fibrosing alopecia syndrome: how a single word name change might change so much. Int J Dermatol. 2025;64:1157-1160. doi:10.1111/ijd.17767.	Did not assess comorbidities
**80**	Werner B, Soares VC, Mulinari-Brenner F, Tolkachjov SN. Understanding fibrosing alopecia in a pattern distribution: a retrospective study with histological challenges in the diagnosis of diffuse central alopecia. Arch Dermatol Res. 2025;317:506.	Did not assess comorbidities

ID	Article	Reason for exclusion
	doi:10.1007/s00403-025-04014-3.	
**81**	Leecharoen W, Thanomkitti K, Thuangtong R, Varothai S, Triwongwaranat D, Jiamton S, Kulthanan K. Use of facial care products and frontal fibrosing alopecia: coincidence or true association? J Dermatol. 2021;48:1557-1563. doi:10.1111/1346-8138.16063.	Association-focused design
**82**	Ramos PM, Anzai A, Duque-Estrada B, et al. Risk factors for frontal fibrosing alopecia: a case-control study in a multiracial population. J Am Acad Dermatol. 2021;84:712-718. doi:10.1016/j.jaad.2020.08.076.	Association-focused design
**83**	Porriño-Bustamante ML, Fernández-Pugnaire MA, Arias-Santiago S. A cross-sectional study of rosacea and risk factors in women with frontal fibrosing alopecia. Acta Derm Venereol. 2019;99:1099-1104. doi:10.2340/00015555-3286.	Association-focused design
**84**	Moreno-Arrones OM, Saceda-Corralo D, Rodrigues-Barata AR, et al. Risk factors associated with frontal fibrosing alopecia: a multicentre case–control study. Clin Exp Dermatol. 2019;44:404-410. doi:10.1111/ced.13785.	Association-focused design
**85**	Aldoori N, Dobson K, Holden CR, McDonagh AJ, Harries M, Messenger AG. Frontal fibrosing alopecia: possible association with leave-on facial skin care products and sunscreens; a questionnaire study. Br J Dermatol. 2016;175:762-767. doi:10.1111/bjd.14535.	Association-focused design
**86**	Rudnicka L, Rokni GR, Lotti T, et al. Allergic contact dermatitis in patients with frontal fibrosing alopecia: an international multicenter study. Dermatol Ther. 2020;33:e13560. doi:10.1111/dth.13560.	Association-focused design
**87**	Buendía-Castaño D, Saceda-Corralo D, Moreno-Arrones OM, et al. Hormonal and gynecological risk factors in frontal fibrosing alopecia: a case-control study. Skin Appendage Disord. 2018;4:274-276. doi:10.1159/000484210.	Association-focused design
**88**	Nasiri S, Dadkhahfar S, Mansouri P, et al. Evaluation of serum level of sex hormones in women with frontal fibrosing alopecia in comparison to healthy controls. Dermatol Ther. 2020;33:e13842. doi:10.1111/dth.13842.	Association-focused design
**89**	Bazotti LX, Teixeira LM, Naspolini AP. Risk factors correlated with frontal fibrosing alopecia in Criciuma, Santa Catarina: a case-control study. Surg Cosmet Dermatol. 2022;14:e20220042. doi:10.5935/scd1984-8773.2022140042.	Association-focused design
**90**	Arasu A, Meah N, Eisman S, Wall D, Sinclair R. Vitamin D status in patients with frontal fibrosing alopecia: a retrospective study. JAAD Int. 2022;7:129-130. doi:10.1016/j.jdin.2022.03.008.	Association-focused design
**91**	Donati A, Lindgren BR, Abreu G, Hordinsky M. Prevalence of frontal fibrosing alopecia among Brazilian dermatologists: a cross-sectional survey. J Am Acad Dermatol. 2020;83:148-150.	Association-focused design
**92**	Porriño-Bustamante ML, Montero-Vílchez T, Pinedo-Moraleda FJ, Fernández-Flores Á, Fernández-Pugnaire MA, Arias-Santiago S. Frontal fibrosing alopecia and sunscreen use: a cross-sectional study of actinic damage. Acta Derm Venereol. 2022;102:adv00757. doi:10.2340/actadv.v102.306.	Association-focused design
**93**	Chen LC, Chung HJ. Increased risk of allergic contact dermatitis in patients with cutaneous lichen planus and lichen planopilaris, but not cutaneous lupus erythematosus: an All of Us database analysis. Arch Dermatol Res. 2024;316:440. doi:10.1007/s00403-024-03175-x.	Association-focused design

ID	Article	Reason for exclusion
94	Chen L, Senna M, Chung H. Elevated risk of allergic contact dermatitis associated with cutaneous lichen planus and lichen planopilaris, but not cutaneous lupus erythematosus. J Invest Dermatol. 2022;142:S153.	Association-focused design
95	Lim SH, Kang H, Heo YW, Lee WS, Lee S. Prevalence and incidence of comorbid diseases and mortality risk associated with lichen planopilaris: a Korean nationwide population-based study. Clin Exp Dermatol. 2023;48:1230-1237. doi:10.1093/ced/llad235.	Association-focused design
96	Joshi TP, Duruewuru A, Holla S, Naqvi Z, Zhu H, Ren V. Comorbidities associated with lichen planopilaris: a case–control study using the All of Us database. Int J Dermatol. 2023;62:e396-e397. doi:10.1111/ijd.16433.	Association-focused design
97	Lim SH, Koh SB, Lee WS, Lee S. Rapidly increasing incidence and prevalence of lichen planopilaris in an Asian population: a Korean nationwide population-based study. J Dermatol. 2022;49:1351-1353. doi:10.1111/1346-8138.16498.	Association-focused design
98	Nasimi M, Garmaroudi G, Ghiasi M, Lajevardi V, Fooladi Z, Ansari MS. Comorbidities in patients with lichen planopilaris: a case-control study. Skin Appendage Disord. 2022;8:302-306. doi:10.1159/000522371.	Association-focused design
99	Conic RRZ, Maghfour J, Damiani G, Bergfeld W. Exploring the association between lichen planopilaris, cardiovascular and metabolic disorders. J Eur Acad Dermatol Venereol. 2021;35:e826-e827. doi:10.1111/jdv.17513.	Association-focused design
100	Nasimi M, Lajevardi V, Mahmoudi H, Arbab F, Shakoei S. Metabolic syndrome in patients with oral lichen planus and lichen planopilaris: a cross-sectional study. Iran J Dermatol. 2021;24:186-192. doi:10.22034/ijd.2020.229042.1082.	Association-focused design
101	Manatis-Lornell A, Okhovat JP, Marks DH, Hagigeorges D, Senna MM. Comorbidities in patients with lichen planopilaris: a retrospective case-control study. J Am Acad Dermatol. 2020;83:205-207. doi:10.1016/j.jaad.2019.07.018.	Association-focused design
102	Brankov N, Conic RZ, Atanaskova-Mesinkovska N, Piliang M, Bergfeld WF. Comorbid conditions in lichen planopilaris: a retrospective data analysis of 334 patients. Int J Womens Dermatol. 2018;4:180-184. doi:10.1016/j.ijwd.2018.04.001.	Association-focused design
103	Conic RZ, Piliang M, Atanaskova-Mesinkovska N, Bergfeld WF. Association of lichen planopilaris with dyslipidemia. JAMA Dermatol. 2015;151:452-453. doi:10.1001/jamadermatol.2014.3781.	Association-focused design
104	Toossi P, Sebti A, Sarvghadi F. Thyroid diseases and lichen planopilaris: a case-control study. Iran J Dermatol. 2015;18:104-107.	Association-focused design
105	Mesinkovska NA, Brankov N, Piliang M, Kyei A, Bergfeld WF. Association of lichen planopilaris with thyroid disease: a retrospective case-control study. J Am Acad Dermatol. 2014;70:889-892. doi:10.1016/j.jaad.2013.12.014.	Association-focused design
106	Yu DA, Kim SR, Cho SI, Kwon O. Endocrine and metabolic comorbidities in primary cicatricial alopecia: a nationwide population-based study. J Dermatol. 2024;51:429-440. doi:10.1111/1346-8138.17080.	Association-focused design
107	Ogbutor C, Chen LC, Kelley KJ, Senna MM. Uterine and ovarian pathologies among patients with lichen planopilaris and frontal fibrosing alopecia. Clin Exp Dermatol. 2025;50:1877-1880. doi:10.1093/ced/llaf210.	Association-focused design

ID	Article	Reason for exclusion
**108**	Trager MH, Lavian J, Lee EY, Gary D, Jenkins F, Christiano AM, Bordone LA. Medical comorbidities and sex distribution among patients with lichen planopilaris and frontal fibrosing alopecia: a retrospective cohort study. J Am Acad Dermatol. 2021;84:1686-1689. doi:10.1016/j.jaad.2020.08.015.	Association-focused design
**109**	Fertig RM, Hu S, Maddy AJ, Balaban A, Aleid N, Aldahan A, Tosti A. Medical comorbidities in patients with lichen planopilaris: a retrospective case-control study. Int J Dermatol. 2018;57:804-809. doi:10.1111/ijd.13946.	Association-focused design
**110**	Nguyen TQ, Tsai M, Grogan T, Goh C. Thyroid disease in alopecia areata, lichen planopilaris, and frontal fibrosing alopecia: a case-control study of 144 patients. US Endocrinol. 2016;12:85-86.	Association-focused design
**111**	Kim SR, Yu D-A, Cho SI, Kwon O. Association of primary cicatricial alopecia with subsequent cardiovascular disease. J Invest Dermatol. 2024;144:1166-1169. doi:10.1016/j.jid.2023.10.021.	Mixed LPP and FFA population
**112**	Jerjen R, Pinczewski J, Sinclair R, Bhoyrul B. Clinicopathological characteristics and treatment outcomes of fibrosing alopecia in a pattern distribution: a retrospective cohort study. J Eur Acad Dermatol Venereol. 2021;35:2440-2447. doi:10.1111/jdv.17604.	Mixed LPP and FFA population
**113**	Panchaprateep R, Ruxrungtham P, Chancheewa B, Asawanonda P. Clinical characteristics, trichoscopy, histopathology and treatment outcomes of frontal fibrosing alopecia in an Asian population: a retro-prospective cohort study. J Dermatol. 2020;47:1301-1311. doi:10.1111/1346-8138.15517.	Mixed LPP and FFA population
**114**	Mansouri P, Rohani Nasab M, Safaei Naraghi Z, Shakoei S, Chalangari R, Martits-Chalangari K, Rahbar MR. Facial papules are early sign of frontal fibrosing alopecia: a cross-sectional study. Acta Med Iran. 2020;58(6):285-290.	Mixed LPP and FFA population
**115**	Kusano L. Epidemiological profile of frontal fibrosing alopecia. J Am Acad Dermatol. 2017;76(6 Suppl 1):AB123.	Overlapping population
**116**	Saceda-Corralo D, Pindado-Ortega C, Moreno-Arrones OM, Ortega-Quijano D, Fernández-Nieto D, Jiménez-Cauhe J, et al. Association of inflammation with progression of hair loss in women with frontal fibrosing alopecia. JAMA Dermatol. 2020;156(6):700-702. doi:10.1001/jamadermatol.2020.0359.	Overlapping population
**117**	Moreno-Arrones OM, Saceda-Corralo D, Rodrigues-Barata AR, Castellanos-Gonzalez M, Fernandez-Pugnaire MA, Grimalt R, Hermosa-Gelbard A, Bernardez C, Molina-Ruiz AM, Ormaechea-Perez N, Fernandez-Crehuet P, Arias-Santiago S, Alonso MJ, Jaen-Olasolo P, Vano-Galvan S. Factors influencing frontal fibrosing alopecia severity: a multicentre cross-sectional study. J Eur Acad Dermatol Venereol. 2019;33:e315-e316. doi:10.1111/jdv.15590.	Overlapping population
**118**	Lyakhovitsky A, Amichai B, Sizopoulou C, Barzilai A. A case series of 46 patients with lichen planopilaris: demographics, clinical evaluation, and treatment experience. J Dermatolog Treat. 2015;26(3):275-279. doi:10.3109/09546634.2014.933165.	Overlapping population
**119**	Walker CJ, Pathoulas JT, Flanagan KE, Pupo Wiss IM, Senna MM. Characterization of lichen planopilaris in men: a retrospective analysis. J Invest Dermatol. 2021;141(Suppl 1):S103.	Mixed LPP and FFA population

ID	Article	Reason for exclusion
**120**	Burns L, De Souza B, Flynn E, Hagigeorges D, Senna M. Lichen planopilaris and frontal fibrosing alopecia: a possible link with rosacea. J Invest Dermatol. 2020;140(Suppl 1):S58.	Mixed LPP and FFA population
**121**	Babahosseini H, Tavakolpour S, Mahmoudi HR, Balighi K, Teimourpour A, et al. Lichen planopilaris: retrospective study on the characteristics and treatment of 291 patients. J Dermatolog Treat. 2019;30(6):598-604. doi:10.1080/09546634.2018.1542480	Mixed LPP and FFA population
**122**	Soares VC, Mulinari-Brenner F, de Souza TE. Lichen planopilaris epidemiology: a retrospective study of 80 cases. An Bras Dermatol. 2015;90(5):666-670. doi:10.1590/abd1806-4841.20153625.	Mixed LPP and FFA population
**123**	Cevasco NC, Bergfeld WF, Remzi BK, Ramirez de Knott H. A case series of 29 patients with lichen planopilaris: the Cleveland Clinic Foundation experience on evaluation, diagnosis, and treatment. J Am Acad Dermatol. 2007;57(1):47-53. doi:10.1016/j.jaad.2007.01.011.	Mixed LPP and FFA population
**124**	Doche I, Romiti R, Rivitti-Machado MC, Gorbatenko-Roth K, Freese RL, Hordinsky MK. Quality-of-life impairment is not related to disease activity in lichen planopilaris and frontal fibrosing alopecia: results of a preliminary cross-sectional study. J Eur Acad Dermatol Venereol. 2022;36(4):e288-e290. doi:10.1111/jdv.17800.	Overlapping population
**125**	Ali S, Collins M, Pupo Wiss I. Vitamin D deficiency in patients with lichen planopilaris/frontal fibrosing alopecia. J Am Acad Dermatol. 2022;87(3 Suppl 1):AB128.	Mixed LPP and FFA population

Study IDs retain their original numbering.

Supplementary Table S4. Characteristics and comorbidity reporting of studies included in the systematic review of frontal fibrosing alopecia and lichen planopilaris

Abbreviation: NA, not available; NR, not reported

Study ID	Data source	Journal / (year)	Country	Publication type	Study design	Cases	Reported comorbidities	Funding (type) / Conflict of interest
**ID 1**	Ibn Sina University Hospital database / single-center	JAAD International / 2023	Morocco	Letter	Cross-sectional	38	Lichen planus pigmentosus; rosacea; vitiligo; psoriasis; acne; thyroid disorders; dyslipidemia; diabetes; high blood pressure; coronary artery disease; atopy; celiac disease; psoriatic arthritis; epilepsy	None / No
**ID 2**	Hospital- and private-practice dermatology centers database / multicenter	Journal of the European Academy of Dermatology and Venereology / 2023	Brazil	Letter	Case series	104	Lichen planus pigmentosus; thyroid disease; hepatitis C; obesity	None / No
**ID 3**	Centro dermatológico estético, Alicante database / single-center	Journal of Cutaneous Medicine and Surgery / 2023	Spain	Full text	Cross-sectional	118	Hypertension; hypercholesterolemia; hypothyroidism; arthrosis; anxiety; type II diabetes; osteoporosis	None / Yes
**ID 4**	Dermatology clinic University of Turin database / single-center	International Journal of Dermatology / 2022	Italy	Letter	Cross-sectional	13	Rosacea; cutaneous lichen planus; asthma; mucosal lichen planus; atopic dermatitis; contact dermatitis; hypercholesterolemia; hypertension; ulcerative colitis; androgenetic alopecia	None / Yes
**ID 5**	Instituto de	Journal of the	Brazil	Letter	Cross-	50	Lichen planus pigmentosus; rosacea	None / Yes

Study ID	Data source	Journal / (year)	Country	Publication type	Study design	Cases	Reported comorbidities	Funding (type) / Conflict of interest
	Dermatologia Professor Rubem David Azulay database / multicenter	American Academy of Dermatology / 2022			sectional			
**ID 6**	Dermatology departments from multiple academic hospitals in Brazil, Chile, and Colombia / multicenter	Journal of the American Academy of Dermatology / 2022	Brazil	Letter	Cross-sectional	33	Hypertension; anxiety/depression disorder; hypothyroidism; androgenetic alopecia; rosacea; cutaneous lichen planus; nail lichen planus; oral lichen planus; lichen planus pigmentosum; overweight; obesity	None / No
**ID 7**	Dermatology departments from multiple university hospitals in Spain / multicenter	Journal of the American Academy of Dermatology / 2022	Spain	Letter	Cross-sectional	39	Hypothyroidism; benign prostatic hyperplasia; prostate adenocarcinoma; rosacea; cutaneous lichen planus; androgenetic alopecia; rosacea	None / No
**ID 8**	Sapienza University of Rome and University of Padua / multicenter	Journal of Clinical and Aesthetic Dermatology / 2021	Italy	Full text	Case series	8	Lichen sclerosus; hypertension; hypercholesterolemia; psoriasis; alopecia areata; lichen planus; hypothyroidism; hyperthyroidism	None / No
**ID 9**	Istanbul University-Cerrahpaşa database / single-center	Turkiye Klinikleri Journal of Dermatology / 2021	Turkey	Full text	Case series	16	Thyroid disease (Hashimoto disease; hyperthyroidism); hypertension; ankylosing spondyloarthropathy; psoriasis; mixed connective tissue disease	None / No

Study ID	Data source	Journal / (year)	Country	Publication type	Study design	Cases	Reported comorbidities	Funding (type) / Conflict of interest
**ID 10**	Hospital-based dermatology clinics database / multicenter	Journal of the American Academy of Dermatology / 2021	Brazil/Spain	Letter	Case series	38	Autoimmune diseases; lichen planus pigmentosus	None / No
**ID 11**	Sapienza University of Rome database / single-center	Journal of Cosmetic Dermatology / 2021	Italy	Full text	Cross-sectional	119	Genital lichen sclerosus; cutaneous/mucosal/nail lichen planus; hypothyroidism; hypertension; hypercholesterolemia; alopecia areata; androgenetic alopecia	NR / NR
**ID 12**	UK Frontal Fibrosing Alopecia Consortium database (secondary care dermatology departments) / multicenter	British Journal of Dermatology / 2020	United Kingdom	Letter	Cross-sectional	711	Autoimmune thyroid disease; celiac disease; pernicious anemia; lichen planus (cutaneous, oral, vulvar, nails); autoimmune diseases (overall)	Public (NIHR Rare Diseases Translational Research Collaboration; NIHR Biomedical Research Centre) / No
**ID 13**	Faghihi Hospital, Shiraz University of Medical Sciences / single-center	Iranian Journal of Dermatology / 2020	Iran	Full text	Case series	22	Hypothyroidism; arterial hypertension; atopy (allergic rhinitis/asthma); dyslipidemia; ischemic heart disease; diabetes mellitus/prediabetes; rheumatoid arthritis; lichen planus; psychiatric disorders; iron deficiency anemia; duodenal ulcer; migraine; irritable bowel disease	Public (Shiraz University of Medical Sciences) / No
**ID 14**	Hospital Universitario	Actas Dermo-Sifiliográficas	Spain	Full text	Cross-sectional	75	Hypothyroidism; rosacea; oral/genital lichen planus; androgenetic alopecia	NR / No

Study ID	Data source	Journal / (year)	Country	Publication type	Study design	Cases	Reported comorbidities	Funding (type) / Conflict of interest
	Infanta Sofía database / single-center	/ 2020						
**ID 15**	France and Germany dermatology centers / multicenter	Journal of the European Academy of Dermatology and Venereology / 2019	France/Germany	Full text	Cross-sectional	490	Thyroid disease; hypothyroidism; hyperthyroidism; hashimoto thyroiditis; unspecified thyroid disease; lipid metabolic disorders; arterial hypertension; coronary heart disease; autoimmune diseases; vitiligo; cutaneous lupus erythematosus; alopecia areata; atopy; rosacea; psoriasis; diabetes mellitus (total); diabetes mellitus type I; diabetes mellitus type II; diabetes mellitus unknown/unspecified; localized scleroderma; rheumatoid arthritis; polymyalgia rheumatica; systemic sclerosis; Sjögren's syndrome; sharp syndrome; autoimmune hepatitis; giant cell arteritis; coeliac disease; psoriatic arthritis, irritable bowel syndrome; Crohn's disease; ulcerative colitis; mucosal lichen planus; cutaneous lichen planus; nail lichen planus	None / No
**ID 16**	Hospital de Clínicas, Universidade Federal do Paraná database / single-center	Anais Brasileiros de Dermatologia / 2019	Brazil	Letter	Cross-sectional	38	Arterial hypertension; dyslipidemia; depression; thyroid disease; hypothyroidism; anxiety; hepatophaty; diabetes mellitus; cardiopathy; osteoporosis/osteopenia; vitiligo; systemic lupus erythematosus; psoriasis; non-melanoma skin cancer; lichen planus pigmentosus; cutaneous lichen planus; nail lichen planus	None / No
**ID 17**	Goethe University	Journal of International	Germany	Full text	Cross-sectional	12	Hypothyroidism; rheumatoid arthritis; undifferenciated collagenosis;	None / No

Study ID	Data source	Journal / (year)	Country	Publication type	Study design	Cases	Reported comorbidities	Funding (type) / Conflict of interest
	Frankfurt database / single-center	Medical Research / 2019					hemochromatosis; hypertension; dyslipidemia; prolactinoma; allergies; psoriasis; rosacea; lichen planus mucosae; vitiligo; lichen sclerosus	
**ID 18**	Hospital Universitário Clementino Fraga Filho, UFRJ / single-center	Anais Brasileiros de Dermatologia / 2019	Brazil	Full text	Cross-sectional	16	Systemic lupus erythematosus, discoid lupus erythematosus, hypertension, thyroid disease, hypothyroidism, Hashimoto thyroiditis, hyperparathyroidism, lichen planus pigmentosus, vitiligo, diabetes mellitus, psoriasis, oral lichen planus	None / No
**ID 19**	Mayo Clinic database / single-center	Mayo Clinic Proceedings / 2018	United States	Full text	Cross-sectional	148	Hypothyroidism; thyroid disease (overall); hyperlipidemia/dyslipidemia; arterial hypertension; atopy; depression; obesity; osteopenia; osteoporosis; rosacea; impaired fasting glucose/prediabetes; type 2 diabetes mellitus; vitamin D deficiency; anemia; autoimmune diseases (Hashimoto thyroiditis; psoriasis; inflammatory bowel disease; Sjogren syndrome; systemic lupus erythematosus; vitiligo; celiac disease; Graves disease; scleroderma; rheumatoid arthritis; type 1 diabetes mellitus); lichen planus (cutaneous/oral/vulvar); lichen sclerosus; endometriosis; obstructive sleep apnea; acne, depression; seborrheic dermatitis; hypertriglyceridemia	NR / No
**ID 20**	University Hospital Ramón y Cajal database / single-center	Journal of the American Academy of Dermatology / 2018	Spain	Letter	Cross-sectional	103	Rosacea; oral lichen planus; cutaneous lichen planus; vitiligo; morphea; atopic dermatitis; contact dermatitis; psoriasis; erythema nodosum; melasma; primary keratoderma; vasculitis	None / No

Study ID	Data source	Journal / (year)	Country	Publication type	Study design	Cases	Reported comorbidities	Funding (type) / Conflict of interest
**ID 21**	Washington University Division of Dermatology database / single-center	Cutis / 2019	United States	Full text	Cross-sectional	29	Thyroid diseases; hypothyroidism; mucocutaneous lichen planus; psoriasis/psoriatic arthritis; vitiligo; systemic lupus erythematosus; Sjögren syndrome; iritis; ulcerative colitis; breast cancer	None / No
**ID 22**	Mayo Clinic database / single-center	Journal of the American Academy of Dermatology / 2017	United States	Full text	Cross-sectional	7	Hypogonadism; asthma; seasonal allergies; rosacea; seborrheic dermatitis; prostate adenocarcinoma; erectile dysfunction; lichen planus	None / No
**ID 23**	University of British Columbia database / single-center	International Journal of Dermatology / 2014	Canada	Full text	Cross-sectional	62	Hypothyroidism; Hashimoto thyroiditis; rheumatoid arthritis; systemic lupus erythematosus; discoid lupus erythematosus; polymyositis; vitiligo; psoriasis; alopecia areata; lichen planopilaris; lichen planus (oral/cutaneous/genital); female pattern hair loss; male pattern hair loss	Public (Canadian Hair Research Foundation ; Vancouver Hospital Research Foundation) / Yes
**ID 24**	Nelson R. Mandela School of Medicine, University of KwaZulu-Natal database / single-center	British Journal of Dermatology / 2013	South Africa	Letter	Case series	24	Lichen planus pigmentosus; HIV infection	None / No
**ID 25**	Centro Hospitalar do	European Journal of	Portugal	Full text	Cross-sectional	11	Androgenetic alopecia; lichen planus	None / No

Study ID	Data source	Journal / (year)	Country	Publication type	Study design	Cases	Reported comorbidities	Funding (type) / Conflict of interest
	Porto – Hospital de Santo António database / single-center	Dermatology / 2011						
**ID 26**	Royal Hallamshire Hospital, Sheffield database / single-center	British Journal of Dermatology / 2009	United Kingdom	Full text	Cross-sectional	18	Oral lichen planus; cutaneous lichen planus; vulvar lichen planus; thyroid disorders; vitiligo	NR / No
**ID 27**	Hospital-based dermatology departments database / multicenter	Journal of the American Academy of Dermatology / 2014	Spain	Full text	Cross-sectional	355	Dyslipidemia; hypothyroidism; arterial hypertension; osteoporosis; androgenetic alopecia; cutaneous lichen planus; oral lichen planus; lichen planopilaris; alopecia areata; vitiligo; lichen sclerosus	None / No
**ID 28**	University of California San Francisco Hair Center database / single-center	British Journal of Dermatology / 2010	United States / United Kingdom	Full text	Cross-sectional	36	Lichen planopilaris; oral lichen planus; vulvar lichen planus; cutaneous lichen planus; androgenetic alopecia; alopecia areata; central centrifugal cicatricial alopecia; telogen effluvium	None / No
**ID 29**	University of Bologna database / single-center	Journal of the European Academy of Dermatology and Venereology / 2019	Italy	Full text	Cross-sectional	65	thyroid disease; vitiligo; Sjögren syndrome; alopecia areata; systemic lupus erythematosus; scleroderma; atopy; lichen planopilaris; cutaneous lichen planus; genital lichen sclerosus; female pattern hair loss	None / No
**ID 30**	Ramathibodi Hospital,	International Journal of	Thailand	Full text	Cross-sectional	56	Diabetes mellitus; hypertension; dyslipidemia; hyperthyroidism; lichen planus	None / No

Study ID	Data source	Journal / (year)	Country	Publication type	Study design	Cases	Reported comorbidities	Funding (type) / Conflict of interest
	Mahidol University database / single-center	Dermatology / 2020					pigmentosus; lichen planus; discoid lupus erythematosus; androgenetic alopecia; seborrheic dermatitis; lichen planopilaris	
**ID 31**	Hospital do Servidor Público Municipal de São Paulo database / single-center	Anais Brasileiros de Dermatologia / 2022	Brazil	Letter	Cross-sectional	27	Arterial hypertension; diabetes mellitus; dyslipidemia; hypothyroidism; lichen planus pigmentosus	None / No
**ID 32**	Southern General Hospital, Glasgow database / single-center	Journal of the American Academy of Dermatology / 2012	United Kingdom	Full text	Cross-sectional	60	Hypothyroidism; autoimmune diseases (overall); pernicious anemia; Sjögren syndrome; alopecia areata; oral lichen planus; cutaneous lichen planus; vulvar lichen sclerosus; atopy	None / No
**ID 33**	St John's Institute of Dermatology, London database / single-center	British Journal of Dermatology / 2022	United Kingdom	Letter	Cross-sectional	17	Androgenetic alopecia; autoimmune diseases (overall); vitiligo; lichen sclerosus; oral lichen planus; erectile dysfunction; autoimmune thyroid disease	Public (British Skin Foundation; Alopecia UK; NIH-equivalent public grants) / No
**ID 34**	Hospital-based dermatology departments database / multicenter	Journal of the European Academy of Dermatology and Venereology /	Spain	Letter	Case series	12	Androgenetic alopecia; hypothyroidism	NR / NR

Study ID	Data source	Journal / (year)	Country	Publication type	Study design	Cases	Reported comorbidities	Funding (type) / Conflict of interest
		2017						
**ID 35**	Duke University Hair Disorders Research and Treatment Center database / single-center	Journal of the American Academy of Dermatology / 2013	United States	Full text	Case series	19	Hypertension; hyperlipidemia; osteoporosis	Public (Duke University Hair Disorders Research and Treatment Center) / Yes
**ID 36**	NYU Langone Health database / single-center	Journal of the American Academy of Dermatology / 2018	United States	Letter	Cross-sectional	92	Autoimmune diseases (overall); hypothyroidism; lichen sclerosus; lichen planus; psoriasis; rosacea; hormonal disorders; psychiatric disorders	None / Yes
**ID 37**	Hospital Universitario Donostia database / single-center	Actas Dermo-Sifiliográficas / 2016	Spain	Full text	Cross-sectional	12	Hypercholesterolemia; arterial hypertension; autoimmune hepatitis; prostate cancer; androgenetic alopecia; basal cell carcinoma; actinic keratosis, melanoma	NR / No
**ID 38**	University Hospital Ramón y Cajal, Madrid database / single-center	Journal of the American Academy of Dermatology / 2021	Spain	Full text	Case series	224	Rosacea; hypothyroidism	None / No
**ID 39**	Ludwig-Maximilian University Munich database	Facial Plastic Surgery / 2018	Germany	Full text	Cross-sectional	72	Hypothyroidism; arterial hypertension; giant cell arteritis); diabetes mellitus type II; hyperparathyroidism; psoriasis; mucosal lichen planus; alopecia areata; androgenetic	None / No

Study ID	Data source	Journal / (year)	Country	Publication type	Study design	Cases	Reported comorbidities	Funding (type) / Conflict of interest
	/ single-center						alopecia; melanoma; non-melanoma skin cancer; breast cancer; lung cancer; leukemia; polyarthritis	
**ID 40**	Hospital General Universitario de Ciudad Real database / single-center	Life / 2023	Spain	Full text	Cross-sectional	306	Hypothyroidism; autoimmune diseases (overall); alopecia areata; psoriasis; vitiligo; lichen planopilaris; cutaneous lichen planus; oral lichen planus; genital lichen planus; androgenetic alopecia	None / No
**ID 41**	International Dermoscopy Society multicenter registry (dermatology centers) / multicenter	Dermatology Practical & Conceptual / 2022	Multinational	Full text	Cross-sectional	188	Androgenetic alopecia; lichen planopilaris; lichen planus (skin/mucosae/nails)	None / No
**ID 42**	(NYU Grossman School of Medicine; Callender Center for Clinical Research databases / multicenter	British Journal of Dermatology / 2021	United States	Letter	Cross-sectional	140	Hypertension; dyslipidemia; thyroid disease (hypo- or hyperthyroidism); central centrifugal cicatricial alopecia; alopecia areata; systemic lupus erythematosus; seborrheic dermatitis; telogen effluvium; traction alopecia; androgenetic alopecia	None / Yes
**ID 43**	University Hospital Ramón y Cajal, Madrid database / single-center	Acta Dermato-Venereologica / 2023	Spain	Full text	Case series	103	Autoimmune thyroid disease (Hashimoto thyroiditis; Graves disease); rosacea; Sjögren syndrome; pernicious anemia; ankylosing spondylitis; discoid lupus erythematosus; breast cancer	Public (Biomedical Research Institute IRYCIS) / No

Study ID	Data source	Journal / (year)	Country	Publication type	Study design	Cases	Reported comorbidities	Funding (type) / Conflict of interest
**ID 44**	Cairo University & Benha University / multicenter	Journal of the European Academy of Dermatology and Venereology / 2022	Egypt	Letter	Cross-sectional	58	Thyroid dysfunction; rosacea; lichen planus; vitiligo; actinic lichen; photosensitivity; melasma; smoking; telogen effuvium, androgenectic alopecia; alopecia areata, lichen plano pilaris	None / No
**ID 45**	North Eastern Indira Gandhi Regional Institute of Health and Medical Sciences database / single-center	Indian Journal of Dermatology / 2023	India	Full text	Cross-sectional	21	Lichen planus pigmentosus; androgenetic alopecia; lichen planopilaris; hypopigmented macules; guttate hypomelanosis	None / No
**ID 46**	Royal London Hospital, Barts Health NHS Trust database / single-center	International Journal of Trichology / 2018	United Kingdom	Full text	Cross-sectional	40	Lichen planopilaris; lichen planus; Hashimoto thyroiditis; psoriasis; eczema; seborrheic dermatitis; fatty liver disease; breast cancer; female pattern hair loss; androgenetic alopecia; angular cheilitis; perioral dermatitis; hypertrhichosis	None / No
**ID 47**	Charing Cross Hospital, Imperial College Healthcare NHS Trust database / single-center	Clinical and Experimental Dermatology / 2025	United Kingdom	Letter	Case series	10	Uterine leiomyomas	None / No
**ID 48**	Cleveland Clinic Foundation database /	Journal of the American Academy of	United States	Full text	Cross-sectional	58	Vitamin D deficiency	Public (NIH – National

Study ID	Data source	Journal / (year)	Country	Publication type	Study design	Cases	Reported comorbidities	Funding (type) / Conflict of interest
	single-center	Dermatology / 2021						Institute of Arthritis and Musculoskeletal and Skin Diseases) / No
**ID 49**	Sheba Medical Center database / single-center	Dermatology / 2024	Israel	Full text	Cross-sectional	126	Lichen planus; seborrheic dermatitis; vitiligo; atopic dermatitis; psoriasis; rosacea; diabetes mellitus; hypertension; dyslipidemia; obesity; lupus; scleroderma; Sjogren syndrome; Raynaud phenomenon; fibromyalgia; gout; allergic rhinitis; food allergy; allergic conjunctivitis; asthma; chronic obstructive pulmonary disease; ischemic heart disease; arrhythmia; Celiac disease; gastroesophageal reflux disease; peptic ulcer; irritable bowel syndrome; colonic polyp; diverticulitis; benign prostatic hyperplasia; hypothyroidism; Hashimoto thyroiditis; thyroid nodules; migraine; cerebrovascular disease; epilepsy; meningioma; insomnia; anxiety; depression; post-traumatic stress disorder; schizoaffective disorder; basal cell carcinoma; melanoma; breast cancer; cervical cancer; lung cancer; squamous cell carcinoma of the vocal cord; thyroid cancer; autoimmune hemolytic anemia; IgA vasculitis	None / No
**ID 50**	University of	International	United	Letter	Cross-	24	Hypertension; hyperlipidemia; obesity;	None / Yes

Study ID	Data source	Journal / (year)	Country	Publication type	Study design	Cases	Reported comorbidities	Funding (type) / Conflict of interest
	Pennsylvania; University of Alabama at Birmingham database / multicenter	Journal of Women's Dermatology / 2024	States		sectional		gastroesophageal reflux disease; systemic lupus erythematosus; sarcoidosis; asthma; HIV infection; polycystic ovary syndrome; obstructive sleep apnea; atrial fibrillation; meningioma; deep vein thrombosis; migraine; androgenetic alopecia; diverticulitis; tachycardia	
**ID 51**	Razi Hospital, Guilan University of Medical Sciences database / single-center	International Journal of Dermatology and Venereology / 2023	Iran	Full text	Cross-sectional	60	Vitamin D deficiency; hypothyroidism; anemia; asthma and/or allergy; hypertension; diabetes mellitus; vitiligo; obesity; overweight;	None / No
**ID 52**	Mayo Clinic, Rochester, MN database / single-center	International Journal of Dermatology / 2021	United States	Full text	Case series	19	Hypertension; obesity; dyslipidemia; benign prostatic hyperplasia; tubular adenoma; mechanical back pain; degenerative arthritis; erectile dysfunction; gastroesophageal reflux disease; thyroid disease; depression; diabetes mellitus; nephrolithiasis; obstructive sleep apnea; prostatitis; pulmonary nodule; aortic stenosis; essential tremor; bicuspid aortic valve; cataracts; cholelithiasis; coronary artery disease; fasting hyperglycemia; focal segmental glomerulosclerosis; generalized anxiety disorder; gout; growth hormone deficiency; hepatitis C virus infection; hypospadias; IgA nephropathy; intestinal obstruction; atrial fibrillation; peripheral arterial disease; precocious puberty; hyperparathyroidism; seronegative polyarthritis; Tetralogy of Fallot; deep vein	None / No

Study ID	Data source	Journal / (year)	Country	Publication type	Study design	Cases	Reported comorbidities	Funding (type) / Conflict of interest
							thrombosis/ pulmonary embolis; androgenetic alopecia; basal cell carcinoma; dermatitis; acne; condyloma acuminatum; ichthyosis hystrix; lichen sclerosus; seborrheic dermatitis	
**ID 53**	Mayo Clinic, Rochester, MN database / single-center	Mayo Clinic Proceedings / 2020	United States	Full text	Cross-sectional	232	Depression; anxiety; sleep problem; neuropathy; migraines; dementia; thyroid disease; hypothyroidism; Hashimoto thyroiditis; Graves disease; subacute thyroiditis; thyroid nodule; thyroid cancer; adrenal adenoma; hypertension; arthritis; dyslipidemia; coronary artery disease; obesity; varicose veins/ venous stasis; arrhythmia; asthma; chronic obstructive pulmonary disease; gastrointestinal autoimmune diseases; celiac disease; ulcerative colitis; Sjogren syndrome; Crohn disease; type 1 diabetes mellitus; gastroesophageal reflux disease; colon polyps; hepatitis B virus infection; pernicious anemia; pancreatitis; hepatitis C virus infection; osteoporosis; vitamin D deficiency; anemia; renal disease; cataract/ glaucoma/ macular degeneration; rheumatoid arthritis; fibromyalgia; carpal tunnel syndrome; systemic lupus erythematosus; breast cancer; cervical cancer; leukemia; seborrheic keratosis; seborrheic dermatitis; dermatoheliosis; actinic keratosis; compound nevi; dermatofibroma; lentigines; non-melanocytic skin cancer; basal cell carcinoma; squamous cell carcinoma; acne;	NR / No

Study ID	Data source	Journal / (year)	Country	Publication type	Study design	Cases	Reported comorbidities	Funding (type) / Conflict of interest
							verruca vulgaris; rosacea; melanoma; onychodystrophy; hyperpigmentation; xerosis; intertrigo; drug reaction hypersensitivity; calluses; asteatotic dermatitis; atopic dermatitis; epidermal cyst; vitiligo; hidradenitis suppurativa; prurigo nodularis; folliculitis; tinea capitis; ecchymosis; psoriasis; petechiae; steroid atrophy; Schamberg purpura; impetigo; xerostoma; acrochordon; telogen effluvium	
**ID 54**	Mayo Clinic, Rochester, MN database / single-center	Journal of the American Academy of Dermatology / 2017	United States	Abstract	Cross-sectional	99	Dyslipidemia; prostate cancer; hypogonadism or erectile dysfunction; diabetes mellitus; thyroid disease; vitiligo; hepatitis C; Crohn disease; extracranial lichen planus (mucosal, genital, nail)	None / NR
**ID 55**	Cleveland Clinic database / single-center	Journal of the American Academy of Dermatology / 2016	United States	Abstract	Cross-sectional	246	Seborrheic dermatitis; hyperandrogenism	None / NR
**ID 56**	Hair Clinic database, Skin & Cancer Foundation Inc. (tertiary referral center) / single-center	Australasian Journal of Dermatology / 2018	Australia	Full text	Cross-sectional	32	Associated lichen planus (cutaneous, oral, genital, nail)	NR / No
**ID 57**	Hospital-based dermatology center database (single	Journal of the European Academy of Dermatology	Brazil	Letter	Cross-sectional	241	Hashimoto thyroiditis; hyperparathyroidism; hyperthyroidism; systemic lupus erythematosus; scleroderma; dermatomyositis; vitiligo; psoriasis; alopecia	None / Yes

Study ID	Data source	Journal / (year)	Country	Publication type	Study design	Cases	Reported comorbidities	Funding (type) / Conflict of interest
	dermatology center) / single-center	and Venereology / 2024					areata; urticaria; hypertension; dyslipidemia; type 2 diabetes mellitus; stroke; myocardial infarction; keratosis pilaris; rosacea-like dermatosis; androgenetic alopecia; lichen planus pigmentosus; mucosal lichen planus; cutaneous lichen planus; nail lichen planus; rhinitis; anxiety; depression; epilepsy	
**ID 58**	Hospital-based specialty hair clinics database (multiple academic centers in the United States) / multicenter	Journal of the American Academy of Dermatology / 2023	United States	Letter	Cross-sectional	270	Asthma; atopic dermatitis; contact dermatitis; medication allergy; seasonal/environmental allergies; urticaria; extracranial lichen planus (cutaneous, oral, genital, nail); androgenetic alopecia; seborrheic dermatitis; alopecia areata; discoid lupus erythematosus; folliculitis decalvans; scalp psoriasis; hypertension; dyslipidemia; type 2 diabetes mellitus; tobacco use	None / No
**ID 59**	University of Chile Clinical Hospital and private practice database / single-center	Clinical and Experimental Dermatology / 2017	Chile	Full text	Cross-sectional	103	Lichen planus	None / No
**ID 60**	Charité – Universitätsmedizin Berlin database / single-center	Journal der Deutschen Dermatologischen Gesellschaft / 2014	Germany	Full text	Cross-sectional	104	Arterial hypertension; dyslipidemia; diabetes mellitus; thyroid disease; vitiligo; alopecia areata; classic lichen planus; depression; iron deficiency; viral hepatitis (B or C); seborrheic dermatitis; COPD; coronary heart disease; breast cancer	None / No

Supplementary Table S5. Comorbidities reported in prevalence studies of patients with lichen planopilaris and frontal fibrosing alopecia.

Comorbidities	ID	Subtype	Study population, N	Mean age, y	Female, n	Events, n	Events, %
**Cardiovascular risk factors**							
**Hypertension**	Carrascoza et al. (2024)	FFA	138	NR	133	9	6,5%
	Meinhard et al. (2014)	FFA	32	NR	31	12	37,5%
	Ali et al. (2023)	FFA	38	53	38	7	18,4%
	García et al. (2023)	FFA	118	64	115	31	26,3%
	Moussa et al. (2022)	FFA	13	40,5	0	1	7,7%
	Doche et al. (2022)	FFA	33	53,1	0	8	24,2%
	Rossi et al. (2021)	FFA	8	58,8	0	3	37,5%
	Uzuncakmak et al. (2021)	FFA	16	51,9	16	3	18,8%
	Grassi et al. (2021)	FFA	119	NR	111	19	16%
	Aslani et al. (2020)	FFA	22	46	22	4	18,2%
	Kanti et al. (2019)	FFA	490	65	467	89	18,2%
	Kusano & Mulinari–Brenner (2019)	FFA	38	61,1	38	18	47,36%
	Valesky et al. (2019)	FFA	12	70,3	12	6	50%
	Secchin et al. (2019)	FFA	16	62	16	8	50%
	Imhof et al. (2018)	FFA	148	62,1	148	55	37,2%
	Vañó–Galván et al. (2014)	FFA	355	61	343	32	9%
	Suchonwanit et al. (2020)	FFA	56	51,3	54	8	14,2%

Comorbidities	ID	Subtype	Study population, N	Mean age, y	Female, n	Events, n	Events, %
	Rocha et al. (2022)	FFA	27	63,8	26	18	66,7%
	Ladizinski et al. (2013)	FFA	19	55,9	19	5	26,3%
	Ormaechea–Pérez et al. (2016)	FFA	12	75	0	3	25%
	Heppt et al. (2018)	FFA	72	62	70	21	29,2%
	Adotama et al. (2021)	FFA	140	NR	140	8	5,8%
	Lyakhovitsky et al. (2024)	LPP	126	47,92	89	15	11,9%
	Oak et al. (2024)	LPP	24	48,4	24	10	41,7%
	Gharaei Nejad et al. (2023)	LPP	60	43,6	44	10	16,7%
	Cantwell et al. (2021)	LPP	19	51,1	0	7	36,8%
	Larkin et al. (2020)	LPP	232	59,8	232	51	22%
	Carrascoza et al. (2024)	LPP	103	NR	68	6	5,8%
	Meinhard et al. (2014)	LPP	71	NR	59	25	35,2%
**Hyperglycemia / prediabetes**	Doche et al. (2022)	FFA	33	53,1	0	2	6%
	Aslani et al. (2020)	FFA	22	46	22	3	13,6%
	Imhof et al. (2018)	FFA	148	62,1	148	20	13,5%
	Cantwell et al. (2021)	LPP	19	51,1	0	1	5,3%
**Diabetes (overall)**	Meinhard et al. (2014)	FFA	32	NR	31	1	3,1%
	Ali et al. (2023)	FFA	38	53	38	3	7,9%
	Kanti et al. (2019)	FFA	490	65	467	16	3,3%
	Kusano & Mulinari–Brenner (2019)	FFA	38	61,1	38	2	5,26%

Comorbidities	ID	Subtype	Study population, N	Mean age, y	Female, n	Events, n	Events, %
	Secchin et al. (2019)	FFA	16	62	16	2	12,5%
	Suchonwanit et al. (2020)	FFA	56	51,3	54	10	17,8%
	Rocha et al. (2022)	FFA	27	63,8	26	8	29,6%
	Lyakhovitsky et al. (2024)	LPP	126	47,92	89	7	5,6%
	Gharaei Nejad et al. (2023)	LPP	60	43,6	44	7	11,7%
	Chaudhry et al. (2017)	LPP	99	47,8	0	6	6%
	Meinhard et al. (2014)	LPP	71	NR	59	3	4,2%
**Type 1 diabetes mellitus**	Kanti et al. (2019)	FFA	490	65	467	4	0,8%
	Imhof et al. (2018)	FFA	148	62,1	148	1	0,7%
**Type 2 diabetes mellitus**	Carrascoza et al. (2024)	FFA	138	NR	133	4	2,9%
	García et al. (2023)	FFA	118	64	115	8	6,8%
	Kanti et al. (2019)	FFA	490	65	467	11	2,3%
	Imhof et al. (2018)	FFA	148	62,1	148	5	3,4%
	Heppt et al. (2018)	FFA	72	62	70	2	2,8%
	Cantwell et al. (2021)	LPP	19	51,1	0	2	10,5%
	Carrascoza et al. (2024)	LPP	103	NR	68	5	4,8%
**Dyslipidemia**	Carrascoza et al. (2024)	FFA	138	NR	133	10	7,2%
	Meinhard et al. (2014)	FFA	32	NR	31	3	9,4%
	Ali et al. (2023)	FFA	38	53	38	8	21,1%
	García et al. (2023)	FFA	118	64	115	24	20,3%

Comorbidities	ID	Subtype	Study population, N	Mean age, y	Female, n	Events, n	Events, %
	Moussa et al. (2022)	FFA	13	40,5	0	1	7,7%
	Rossi et al. (2021)	FFA	8	58,8	0	1	12,5%
	Grassi et al. (2021)	FFA	119	NR	111	17	14,3%
	Aslani et al. (2020)	FFA	22	46	22	3	13,6%
	Kanti et al. (2019)	FFA	490	65	467	105	21,4%
	Kusano & Mulinari–Brenner (2019)	FFA	38	61,1	38	14	36,84%
	Valesky et al. (2019)	FFA	12	70,3	12	2	16,7%
	Imhof et al. (2018)	FFA	148	62,1	148	67	45,3%
	Vañó–Galván et al. (2014)	FFA	355	61	343	89	25%
	Suchonwanit et al. (2020)	FFA	56	51,3	54	7	12,5%
	Rocha et al. (2022)	FFA	27	63,8	26	8	29,6%
	Ladizinski et al. (2013)	FFA	19	55,9	19	2	10,5%
	Ormaechea–Pérez et al. (2016)	FFA	12	75	0	2	17%
	Adotama et al. (2021)	FFA	140	NR	140	9	6,5%
	Lyakhovitsky et al. (2024)	LPP	126	47,92	89	28	22,2%
	Oak et al. (2024)	LPP	24	48,4	24	4	16,7%
	Cantwell et al. (2021)	LPP	19	51,1	0	6	31,6%
	Larkin et al. (2020)	LPP	232	59,8	232	36	15,5%
	Chaudhry et al. (2017)	LPP	99	47,8	0	18	18,2%
	Carrascoza et al. (2024)	LPP	103	NR	68	3	2,9%

Comorbidities	ID	Subtype	Study population, N	Mean age, y	Female, n	Events, n	Events, %
	Meinhard et al. (2014)	LPP	71	NR	59	9	12,7%
**Hypertriglyceridemia**	Imhof et al. (2018)	FFA	148	62,1	148	18	12,2%
**Overweight**	Doche et al. (2022)	FFA	33	53,1	0	12	36,3%
	Gharaei Nejad et al. (2023)	LPP	60	43,6	44	29	48,3%
**Obesity**	Brito et al. (2023)	FFA	104	60,5	101	19	19,2%
	Doche et al. (2022)	FFA	33	53,1	0	2	6,1%
	Imhof et al. (2018)	FFA	148	62,1	148	27	18,2%
	Lyakhovitsky et al. (2024)	LPP	126	47,92	89	13	10,3%
	Oak et al. (2024)	LPP	24	48,4	24	2	8,3%
	Gharaei Nejad et al. (2023)	LPP	60	43,6	44	8	13,3%
	Cantwell et al. (2021)	LPP	19	51,1	0	7	36,8%
	Larkin et al. (2020)	LPP	232	59,8	232	17	7,3%
**Fatty liver**	Gkini et al. (2018)	FFA	40	65,9	40	1	2,5%
**Cardiovascular disease**							
**Cardiopathy**	Kusano & Mulinari–Brenner (2019)	FFA	38	61,1	38	2	5,26%
**Cardiac disease**	Lyakhovitsky et al. (2024)	LPP	126	47,92	89	3	2,4%
**Ischemic heart disease**	Carrascoza et al. (2024)	FFA	138	NR	133	0	0%
	Aslani et al. (2020)	FFA	22	46	22	3	13,6%
	Lyakhovitsky et al. (2024)	LPP	126	47,92	89	1	0,8%
	Carrascoza et al. (2024)	LPP	103	NR	68	1	0,9%

Comorbidities	ID	Subtype	Study population, N	Mean age, y	Female, n	Events, n	Events, %
**Coronary artery disease**	Meinhard et al. (2014)	FFA	32	NR	31	3	9,4%
	Ali et al. (2023)	FFA	38	53	38	1	2,6%
	Kanti et al. (2019)	FFA	490	65	467	30	6,1%
	Cantwell et al. (2021)	LPP	19	51,1	0	1	5,3%
	Larkin et al. (2020)	LPP	232	59,8	232	18	7,8%
	Meinhard et al. (2014)	LPP	71	NR	59	2	2,8%
**Congenital heart disease (Tetralogy of Fallot)**	Cantwell et al. (2021)	LPP	19	51,1	0	1	5,3%
**Arrhythmia**	Lyakhovitsky et al. (2024)	LPP	126	47,92	89	2	1,6%
	Larkin et al. (2020)	LPP	232	59,8	232	12	5,2%
**Atrial fibrillation / paroxysmal AF**	Oak et al. (2024)	LPP	24	48,4	24	1	4,2%
	Cantwell et al. (2021)	LPP	19	51,1	0	1	5,3%
**Tachycardia**	Oak et al. (2024)	LPP	24	48,4	24	1	4,2%
**Valvular disease**	Cantwell et al. (2021)	LPP	19	51,1	0	1	5,3%
**Cerebrovascular disease**							
**Cerebrovascular incident / stroke**	Carrascoza et al. (2024)	FFA	138	NR	133	0	0%
	Lyakhovitsky et al. (2024)	LPP	126	47,92	89	1	0,8%
	Carrascoza et al. (2024)	LPP	103	NR	68	2	1,9%
**Peripheral vascular disease**							
**Peripheral artery**	Cantwell et al. (2021)	LPP	19	51,1	0	1	5,3%

Comorbidities	ID	Subtype	Study population, N	Mean age, y	Female, n	Events, n	Events, %
**disease**							
**Varicose veins / venous stasis**	Larkin et al. (2020)	LPP	232	59,8	232	15	6,5%
**Deep vein thrombosis**	Oak et al. (2024)	LPP	24	48,4	24	1	4,2%
**Thromboembolic disease**	Cantwell et al. (2021)	LPP	19	51,1	0	1	5,3%
**Other hair disorders**							
**Nonscarring alopecias**							
**Androgenetic alopecia**	Carrascoza et al. (2024)	FFA	138	NR	133	12	8,7%
	Moussa et al. (2022)	FFA	13	40,5	0	3	23,1%
	Doche et al. (2022)	FFA	33	53,1	0	21	63,6%
	Lobato–Berezo et al. (2022)	FFA	39	69	0	30	76,9%
	Rossi et al. (2021)	FFA	8	58,8	0	4	50%
	Grassi et al. (2021)	FFA	119	NR	111	19	16%
	Maldonado Cid et al. (2020)	FFA	75	61	73	42	56,8%
	Banka et al. (2014)	FFA	62	NR	61	43	70%
	Conde Fernandes et al. (2011)	FFA	11	64,9	11	3	27,3%
	Vañó–Galván et al. (2014)	FFA	355	61	343	144	40%
	Samrao et al. (2010)	FFA	36	60	35	5	14%
	Starace et al. (2022)	FFA	188	62	185	73	38,8%
	Suchonwanit et al. (2020)	FFA	56	51,3	54	7	12,5%

Comorbidities	ID	Subtype	Study population, N	Mean age, y	Female, n	Events, n	Events, %
	Rayinda et al. (2022)	FFA	17	44	0	3	18%
	Alegre–Sánchez et al. (2017)	FFA	12	47,3	0	8	67%
	Ladizinski et al. (2013)	FFA	19	55,9	19	2	11%
	Ormaechea–Pérez et al. (2016)	FFA	12	75	0	10	83%
	Heppt et al. (2018)	FFA	72	62	70	25	34,7%
	Adotama et al. (2021)	FFA	140	NR	140	17	12,3%
	Dorgham et al. (2022)	FFA	58	50,5	58	6	10,3%
	Verma et al. (2023)	FFA	21	48,3	19	2	9,5%
	Gkini et al. (2018)	FFA	40	65,9	40	15	37,5%
	Oak et al. (2024)	LPP	24	48,4	24	12	50%
	Cantwell et al. (2021)	LPP	19	51,1	0	5	26,3%
	Carrascoza et al. (2024)	LPP	103	NR	68	14	13,6%
**Alopecia areata**	Carrascoza et al. (2024)	FFA	138	NR	133	1	0,7%
	Meinhard et al. (2014)	FFA	32	NR	31	1	3,1%
	Rossi et al. (2021)	FFA	8	58,8	0	1	12,5%
	Grassi et al. (2021)	FFA	119	NR	111	5	4,2%
	Kanti et al. (2019)	FFA	490	65	467	2	0,4%
	Banka et al. (2014)	FFA	62	NR	61	8	13%
	Vañó–Galván et al. (2014)	FFA	355	61	343	2	0,56%
	Samrao et al. (2010)	FFA	36	60	35	2	6%

Comorbidities	ID	Subtype	Study population, N	Mean age, y	Female, n	Events, n	Events, %
	Starace et al. (2019)	FFA	65	62,5	65	2	3,1%
	MacDonald et al. (2012)	FFA	60	64	60	1	1,7%
	Heppt et al. (2018)	FFA	72	62	70	1	1,4%
	Carmona–Rodriguez et al. (2023)	FFA	306	68,3	296	12	3,9%
	Adotama et al. (2021)	FFA	140	NR	140	8	5,8%
	Dorgham et al. (2022)	FFA	58	50,5	58	2	3,4%
	Carrascoza et al. (2024)	LPP	103	NR	68	0	0%
	Meinhard et al. (2014)	LPP	71	NR	59	1	1,4%
**Telogen effluvium**	Samrao et al. (2010)	FFA	36	60	35	1	2,8%
	Adotama et al. (2021)	FFA	140	NR	140	6	4,3%
	Dorgham et al. (2022)	FFA	58	50,5	58	18	31%
	Larkin et al. (2020)	LPP	232	59,8	232	1	0,4%
**Scarring alopecias**							
**Traction alopecia**	Adotama et al. (2021)	FFA	140	NR	140	0	0%
**Central centrifugal cicatricial alopecia**	Samrao et al. (2010)	FFA	36	60	35	1	2,8%
	Adotama et al. (2021)	FFA	140	NR	140	3	2,2%
**Lichen planopilaris**	Samrao et al. (2010)	FFA	36	60	35	5	14%
	Starace et al. (2019)	FFA	65	62,5	65	15	23%
	Starace et al. (2022)	FFA	188	62	185	35	19%
	Suchonwanit et al. (2020)	FFA	56	51,3	54	1	1,8%

Comorbidities	ID	Subtype	Study population, N	Mean age, y	Female, n	Events, n	Events, %
	MacDonald et al. (2012)	FFA	60	64	60	5	8%
	Carmona–Rodriguez et al. (2023)	FFA	306	68,3	296	30	9,8%
	Dorgham et al. (2022)	FFA	58	50,5	58	11	19%
	Verma et al. (2023)	FFA	21	48,3	19	2	9,5%
	Gkini et al. (2018)	FFA	40	65,9	40	3	7,5%
**Inflammatory dermatoses**							
**Psoriasis**	Carrascoza et al. (2024)	FFA	138	NR	133	2	1,4%
	Ali et al. (2023)	FFA	38	53	38	1	2,6%
	Rossi et al. (2021)	FFA	8	58,8	0	1	12,5%
	Uzuncakmak et al. (2021)	FFA	16	51,9	16	1	6,3%
	Kanti et al. (2019)	FFA	490	65	467	3	0,6%
	Kusano & Mulinari–Brenner (2019)	FFA	38	61,1	38	1	2,63%
	Valesky et al. (2019)	FFA	12	70,3	12	2	16,7%
	Secchin et al. (2019)	FFA	16	62	16	1	6,25%
	Imhof et al. (2018)	FFA	148	62,1	148	11	7,4%
	Pindado–Ortega et al. (2018)	FFA	103	55,8	103	1	1%
	Zhang et al. (2019)	FFA	29	55,4	28	2	7%
	Banka et al. (2014)	FFA	62	NR	61	2	3%
	Strazzulla et al. (2018)	FFA	92	55	90	2	2,2%
	Heppt et al. (2018)	FFA	72	62	70	2	2,8%

Comorbidities	ID	Subtype	Study population, N	Mean age, y	Female, n	Events, n	Events, %
	Carmona–Rodriguez et al. (2023)	FFA	306	68,3	296	8	2,6%
	Gkini et al. (2018)	FFA	40	65,9	40	1	2,5%
	Lyakhovitsky et al. (2024)	LPP	126	47,92	89	1	0,8%
	Larkin et al. (2020)	LPP	232	59,8	232	1	0,4%
	Carrascoza et al. (2024)	LPP	103	NR	68	0	0%
**Dermatitis (psoriasiform / NOS)**	Cantwell et al. (2021)	LPP	19	51,1	0	3	15,8%
**Rosacea**	Carrascoza et al. (2024)	FFA	138	NR	133	8	5,8%
	Ali et al. (2023)	FFA	38	53	38	11	28,9%
	Moussa et al. (2022)	FFA	13	40,5	0	4	30,8%
	Salas–Callo et al. (2022)	FFA	50	57,7	44	0	0%
	Doche et al. (2022)	FFA	33	53,1	0	11	33,3%
	Lobato–Berezo et al. (2022)	FFA	39	69	0	12	30,8%
	Maldonado Cid et al. (2020)	FFA	75	61	73	15	20%
	Kanti et al. (2019)	FFA	490	65	467	9	1,8%
	Valesky et al. (2019)	FFA	12	70,3	12	1	8,3%
	Imhof et al. (2018)	FFA	148	62,1	148	23	15,5%
	Pindado–Ortega et al. (2018)	FFA	103	55,8	103	35	34%
	Saceda–Corralo et al. (2023)	FFA	103	61	103	28	35,4%
	Tolkachjov et al. (2017)	FFA	7	54,6	0	1	14,3%
	Strazzulla et al. (2018)	FFA	92	55	90	3	3,2%

Comorbidities	ID	Subtype	Study population, N	Mean age, y	Female, n	Events, n	Events, %
	Dorgham et al. (2022)	FFA	58	50,5	58	2	3,4%
	Lyakhovitsky et al. (2024)	LPP	126	47,92	89	1	0,8%
	Larkin et al. (2020)	LPP	232	59,8	232	5	2,2%
	Carrascoza et al. (2024)	LPP	103	NR	68	0	0%
**Perioral dermatitis**	Gkini et al. (2018)	FFA	40	65,9	40	1	2,5%
**Acne**	Ali et al. (2023)	FFA	38	53	38	1	2,6%
	Imhof et al. (2018)	FFA	148	62,1	148	8	5,4%
	Cantwell et al. (2021)	LPP	19	51,1	0	1	5,3%
	Larkin et al. (2020)	LPP	232	59,8	232	6	2,6%
**Seborrheic dermatitis**	Meinhard et al. (2014)	FFA	32	NR	31	0	0%
	Imhof et al. (2018)	FFA	148	62,1	148	27	18,2%
	Tolkachjov et al. (2017)	FFA	7	54,6	0	2	28,6%
	Suchonwanit et al. (2020)	FFA	56	51,3	54	5	8,9%
	Adotama et al. (2021)	FFA	140	NR	140	5	3,6%
	Gkini et al. (2018)	FFA	40	65,9	40	1	2,5%
	Lyakhovitsky et al. (2024)	LPP	126	47,92	89	21	16,7%
	Cantwell et al. (2021)	LPP	19	51,1	0	1	5,3%
	Larkin et al. (2020)	LPP	232	59,8	232	28	12,1%
	Ratnaparkhi et al. (2016)	LPP	246	NR	NR	106	43,1%
	Meinhard et al. (2014)	LPP	71	NR	59	3	4,2%

Comorbidities	ID	Subtype	Study population, N	Mean age, y	Female, n	Events, n	Events, %
**Eczema**	Gkini et al. (2018)	FFA	40	65,9	40	1	2,5%
**Prurigo nodularis**	Larkin et al. (2020)	LPP	232	59,8	232	1	0,4%
**Hidradenitis suppurativa**	Larkin et al. (2020)	LPP	232	59,8	232	1	0,4%
**Lichen spectrum disorders**							
**Lichen planus**	Carrascoza et al. (2024)	FFA	138	NR	133	6	4,3%
	Pathoulas et al. (2023)	FFA	37	56,92	0	0	0%
	Mardones & Shapiro (2017)	FFA	27	NR	26	0	0%
	Meinhard et al. (2014)	FFA	32	NR	31	0	0%
	Moussa et al. (2022)	FFA	13	40,5	0	2	15,4%
	Doche et al. (2022)	FFA	33	53,1	0	2	6%
	Grassi et al. (2021)	FFA	119	NR	111	19	16%
	McSweeney et al. (2020)	FFA	711	66	711	60	9,5%
	Aslani et al. (2020)	FFA	22	46	22	2	9,1%
	Maldonado Cid et al. (2020)	FFA	75	61	73	7	9,6%
	Kanti et al. (2019)	FFA	490	65	467	61	12,4%
	Kusano & Mulinari–Brenner (2019)	FFA	38	61,1	38	23	60,2%
	Valesky et al. (2019)	FFA	12	70,3	12	1	8,33%
	Secchin et al. (2019)	FFA	16	62	16	1	6,25%
	Imhof et al. (2018)	FFA	148	62,1	148	27	18,2%
	Zhang et al. (2019)	FFA	29	55,4	28	8	28%

Comorbidities	ID	Subtype	Study population, N	Mean age, y	Female, n	Events, n	Events, %
	Tolkachjov et al. (2017)	FFA	7	54,6	0	0	0%
	Banka et al. (2014)	FFA	62	NR	61	10	16%
	Conde Fernandes et al. (2011)	FFA	11	64,9	11	0	0%
	Tan and Messenger (2009)	FFA	18	55,5	18	3	17%
	Samrao et al. (2010)	FFA	36	60	35	3	8%
	Starace et al. (2022)	FFA	188	62	185	10	5,3%
	Suchonwanit et al. (2020)	FFA	56	51,3	54	1	1,8%
	Strazzulla et al. (2018)	FFA	92	55	90	1	1,1%
	Dorgham et al. (2022)	FFA	58	50,5	58	2	3,4%
	Gkini et al. (2018)	FFA	40	65,9	40	4	10%
	Lyakhovitsky et al. (2024)	LPP	126	47,92	89	17	13,5%
	Kerkemeyer & Green (2018)	LPP	32	60,5	29	5	15,6%
	Carrascoza et al. (2024)	LPP	103	NR	68	13	12,6%
	Pathoulas et al. (2023)	LPP	215	52,03	0	27	12,56%
	Mardones & Shapiro (2017)	LPP	75	NR	41	2	2,7%
	Meinhard et al. (2014)	LPP	71	NR	59	4	5,6%
**Lichen planus (cutaneous)**	Carrascoza et al. (2024)	FFA	138	NR	133	2	1,4%
	Pathoulas et al. (2023)	FFA	37	56,92	0	0	0%
	Moussa et al. (2022)	FFA	13	40,5	0	2	15,4%
	Doche et al. (2022)	FFA	33	53,1	0	2	6%

Comorbidities	ID	Subtype	Study population, N	Mean age, y	Female, n	Events, n	Events, %
	McSweeney et al. (2020)	FFA	711	66	711	7	1,1%
	Kanti et al. (2019)	FFA	490	65	467	14	3%
	Kusano & Mulinari–Brenner (2019)	FFA	38	61,1	38	21	55%
	Pindado–Ortega et al. (2018)	FFA	103	55,8	103	2	1,9%
	Banka et al. (2014)	FFA	62	NR	61	1	2%
	Tan and Messenger (2009)	FFA	18	55,5	18	1	5,5%
	Vañó–Galván et al. (2014)	FFA	355	61	343	23	6,5%
	Samrao et al. (2010)	FFA	36	60	35	1	2,8%
	Carmona–Rodriguez et al. (2023)	FFA	306	68,3	296	5	1,6%
	Kerkemeyer & Green (2018)	LPP	32	60,5	29	1	3%
	Carrascoza et al. (2024)	LPP	103	NR	68	4	3,9%
	Pathoulas et al. (2023)	LPP	215	52,03	0	16	7,44%
**Lichen planus (oral)**	Pathoulas et al. (2023)	FFA	37	56,92	0	0	0%
	Doche et al. (2022)	FFA	33	53,1	0	0	0%
	Secchin et al. (2019)	FFA	16	62	16	1	6,25%
	Imhof et al. (2018)	FFA	148	62,1	148	16	10,8%
	Pindado–Ortega et al. (2018)	FFA	103	55,8	103	6	5,8%
	Samrao et al. (2010)	FFA	36	60	35	1	2,8%
	Rayinda et al. (2022)	FFA	17	44	0	1	6%
	Carmona–Rodriguez et al. (2023)	FFA	306	68,3	296	10	3,3%

Comorbidities	ID	Subtype	Study population, N	Mean age, y	Female, n	Events, n	Events, %
	Kerkemeyer & Green (2018)	LPP	32	60,5	29	2	6%
	Pathoulas et al. (2023)	LPP	215	52,03	0	11	5,12%
**Lichen planus (genital)**	Pathoulas et al. (2023)	FFA	37	56,92	0	0	0%
	McSweeney et al. (2020)	FFA	711	66	711	22	3,5%
	Imhof et al. (2018)	FFA	148	62,1	148	15	10,1%
	Banka et al. (2014)	FFA	62	NR	61	1	2%
	Tan and Messenger (2009)	FFA	18	55,5	18	3	17%
	Samrao et al. (2010)	FFA	36	60	35	1	2,8%
	Carmona–Rodriguez et al. (2023)	FFA	306	68,3	296	3	1%
	Kerkemeyer & Green (2018)	LPP	32	60,5	29	1	3%
	Pathoulas et al. (2023)	LPP	215	52,03	0	3	1,4%
**Lichen planus (mucosal)**	Carrascoza et al. (2024)	FFA	138	NR	133	3	2,2%
	Moussa et al. (2022)	FFA	13	40,5	0	0	0%
	McSweeney et al. (2020)	FFA	711	66	711	227	32%
	Maldonado Cid et al. (2020)	FFA	75	61	73	5	7%
	Kanti et al. (2019)	FFA	490	65	467	16	3%
	Valesky et al. (2019)	FFA	12	70,3	12	1	8,33%
	Vañó–Galván et al. (2014)	FFA	355	61	343	12	3,40%
	MacDonald et al. (2012)	FFA	60	64	60	1	1,7%
	Heppt et al. (2018)	FFA	72	62	70	3	4,2%

Comorbidities	ID	Subtype	Study population, N	Mean age, y	Female, n	Events, n	Events, %
	Carrascoza et al. (2024)	LPP	103	NR	68	8	7,7%
**Lichen planus (nail)**	Carrascoza et al. (2024)	FFA	138	NR	133	1	0,7%
	Pathoulas et al. (2023)	FFA	37	56,92	0	0	0%
	Doche et al. (2022)	FFA	33	53,1	0	0	0%
	McSweeney et al. (2020)	FFA	711	66	711	2	0,3%
	Kanti et al. (2019)	FFA	490	65	467	31	6%
	Kusano & Mulinari–Brenner (2019)	FFA	38	61,1	38	1	2,6%
	Kerkemeyer & Green (2018)	LPP	32	60,5	29	1	3%
	Carrascoza et al. (2024)	LPP	103	NR	68	1	0,7%
	Pathoulas et al. (2023)	LPP	215	52,03	0	6	2,79%
**Lichen planus pigmentosus**	Carrascoza et al. (2024)	FFA	138	NR	133	22	15,9%
	Ali et al. (2023)	FFA	38	53	38	14	36,8%
	Salas–Callo et al. (2022)	FFA	50	57,7	44	14	28%
	Doche et al. (2022)	FFA	33	53,1	0	1	3%
	Fernandes Melo et al. (2021)	FFA	38	55	37	2	5,2%
	Kusano & Mulinari–Brenner (2019)	FFA	38	61,1	38	1	2,6%
	Secchin et al. (2019)	FFA	16	62	16	5	31,25%
	Dlova et al. (2013)	FFA	44	NR	NR	24	54,5%
	Suchonwanit et al. (2020)	FFA	56	51,3	54	5	8,9%
	Rocha et al. (2022)	FFA	27	63,8	26	6	22%

Comorbidities	ID	Subtype	Study population, N	Mean age, y	Female, n	Events, n	Events, %
	Verma et al. (2023)	FFA	21	48,3	19	6	28,6%
	Carrascoza et al. (2024)	LPP	103	NR	68	1	0,9%
**Lichen sclerosus**	Rossi et al. (2021)	FFA	8	58,8	0	2	25%
	Grassi et al. (2021)	FFA	119	NR	111	15	12,6%
	Valesky et al. (2019)	FFA	12	70,3	12	1	8,3%
	Imhof et al. (2018)	FFA	148	62,1	148	8	5,4%
	Vañó–Galván et al. (2014)	FFA	355	61	343	1	0,28%
	MacDonald et al. (2012)	FFA	60	64	60	1	1,7%
	Rayinda et al. (2022)	FFA	17	44	0	1	6%
	Strazzulla et al. (2018)	FFA	92	55	90	4	4,3%
	Cantwell et al. (2021)	LPP	19	51,1	0	1	5,3%
**Actinic lichen**	Dorgham et al. (2022)	FFA	58	50,5	58	8	13,8%
**Other types of lichen**	Brito et al. (2023)	FFA	104	60,5	101	4	3,9%
**Pigmentation disorders**							
**Vitiligo**	Carrascoza et al. (2024)	FFA	138	NR	133	3	2,2%
	Meinhard et al. (2014)	FFA	32	NR	31	2	6,3%
	Ali et al. (2023)	FFA	38	53	38	1	2,6%
	Kanti et al. (2019)	FFA	490	65	467	12	2,5%
	Kusano & Mulinari–Brenner (2019)	FFA	38	61,1	38	2	5,26%
	Valesky et al. (2019)	FFA	12	70,3	12	1	8,3%

Comorbidities	ID	Subtype	Study population, N	Mean age, y	Female, n	Events, n	Events, %
	Secchin et al. (2019)	FFA	16	62	16	3	18,75%
	Imhof et al. (2018)	FFA	148	62,1	148	4	2,7%
	Pindado–Ortega et al. (2018)	FFA	103	55,8	103	1	1%
	Zhang et al. (2019)	FFA	29	55,4	28	1	3%
	Banka et al. (2014)	FFA	62	NR	61	1	2%
	Tan and Messenger (2009)	FFA	18	55,5	18	1	5,5%
	Vañó–Galván et al. (2014)	FFA	355	61	343	2	0,56%
	Starace et al. (2019)	FFA	65	62,5	65	5	7,7%
	Rayinda et al. (2022)	FFA	17	44	0	1	6%
	Carmona–Rodriguez et al. (2023)	FFA	306	68,3	296	5	1,6%
	Dorgham et al. (2022)	FFA	58	50,5	58	1	1,7%
	Lyakhovitsky et al. (2024)	LPP	126	47,92	89	2	1,6%
	Gharaei Nejad et al. (2023)	LPP	60	43,6	44	3	5%
	Larkin et al. (2020)	LPP	232	59,8	232	1	0,4%
	Chaudhry et al. (2017)	LPP	99	47,8	0	1	1%
	Carrascoza et al. (2024)	LPP	103	NR	68	0	0%
	Meinhard et al. (2014)	LPP	71	NR	59	0	0%
**Melasma**	Pindado–Ortega et al. (2018)	FFA	103	55,8	103	1	1%
	Dorgham et al. (2022)	FFA	58	50,5	58	2	3,4%
**Hyperpigmentation**	Larkin et al. (2020)	LPP	232	59,8	232	4	1,7%

Comorbidities	ID	Subtype	Study population, N	Mean age, y	Female, n	Events, n	Events, %
**Lentigines**	Larkin et al. (2020)	LPP	232	59,8	232	7	3,1%
**Guttate hypomelanosis**	Verma et al. (2023)	FFA	21	48,3	19	1	4,76%
**Atopy**							
**Atopic dermatitis**	Pathoulas et al. (2023)	FFA	37	56,92	0	2	5,41%
	Ali et al. (2023)	FFA	38	53	38	2	5%
	Moussa et al. (2022)	FFA	13	40,5	0	1	7,7%
	Valesky et al. (2019)	FFA	12	70,3	12	1	8,3%
	Pindado–Ortega et al. (2018)	FFA	103	55,8	103	8	7,7%
	Lyakhovitsky et al. (2024)	LPP	126	47,92	89	4	3,2%
	Larkin et al. (2020)	LPP	232	59,8	232	2	0,9%
	Pathoulas et al. (2023)	LPP	215	52,03	0	14	6,51%
**Asthma**	Pathoulas et al. (2023)	FFA	37	56,92	0	3	8,11%
	Moussa et al. (2022)	FFA	13	40,5	0	1	7,7%
	Lyakhovitsky et al. (2024)	LPP	126	47,92	89	1	0,8%
	Oak et al. (2024)	LPP	24	48,4	24	1	4,2%
	Gharaei Nejad et al. (2023)	LPP	60	43,6	44	11	18,3%
	Larkin et al. (2020)	LPP	232	59,8	232	10	4,3%
	Pathoulas et al. (2023)	LPP	215	52,03	0	21	9,77%
**Allergic rhinitis**	Carrascoza et al. (2024)	FFA	138	NR	133	1	0,7%
	Pathoulas et al. (2023)	FFA	37	56,92	0	3	8,11%

Comorbidities	ID	Subtype	Study population, N	Mean age, y	Female, n	Events, n	Events, %
	Lyakhovitsky et al. (2024)	LPP	126	47,92	89	8	6,3%
	Carrascoza et al. (2024)	LPP	103	NR	68	1	0,9%
	Pathoulas et al. (2023)	LPP	215	52,03	0	20	9,3%
**Contact dermatitis**	Pathoulas et al. (2023)	FFA	37	56,92	0	0	0%
	Moussa et al. (2022)	FFA	13	40,5	0	1	7,7%
	Pindado–Ortega et al. (2018)	FFA	103	55,8	103	8	7,7%
	Pathoulas et al. (2023)	LPP	215	52,03	0	8	3,72%
**Allergy**	Valesky et al. (2019)	FFA	12	70,3	12	7	58,3%
**Food allergy**	Lyakhovitsky et al. (2024)	LPP	126	47,92	89	1	0,8%
**Allergic conjunctivitis**	Lyakhovitsky et al. (2024)	LPP	126	47,92	89	1	0,8%
**Atopy (general)**	Aslani et al. (2020)	FFA	22	46	22	4	18,2%
	Kanti et al. (2019)	FFA	490	65	467	17	3,5%
	Imhof et al. (2018)	FFA	148	62,1	148	65	43,9%
	Tolkachjov et al. (2017)	FFA	7	54,6	0	2	28,6%
	Starace et al. (2019)	FFA	65	62,5	65	23	35,4%
	MacDonald et al. (2012)	FFA	60	64	60	9	15%
	Lyakhovitsky et al. (2024)	LPP	126	47,92	89	10	7,90%
**Urticaria**	Carrascoza et al. (2024)	FFA	138	NR	133	3	2,10%
	Pathoulas et al. (2023)	FFA	37	56,92	0	0	0%
	Carrascoza et al. (2024)	LPP	103	NR	68	0	0%

Comorbidities	ID	Subtype	Study population, N	Mean age, y	Female, n	Events, n	Events, %
	Pathoulas et al. (2023)	LPP	215	52,03	0	1	0,47%
**Benign cutaneous lesions**							
**Acrochordon**	Larkin et al. (2020)	LPP	232	59,8	232	1	0,4%
**Epidermal cyst**	Larkin et al. (2020)	LPP	232	59,8	232	2	0,9%
**Seborrheic keratosis**	Larkin et al. (2020)	LPP	232	59,8	232	33	14,2%
**Dermatofibroma**	Larkin et al. (2020)	LPP	232	59,8	232	9	3,9%
**Compound nevi**	Larkin et al. (2020)	LPP	232	59,8	232	13	5,6%
**Calluses**	Larkin et al. (2020)	LPP	232	59,8	232	2	0,9%
**Skin cancer and precancerous skin lesions**							
**Actinic keratosis**	Ormaechea–Pérez et al. (2016)	FFA	12	75	0	2	17%
	Larkin et al. (2020)	LPP	232	59,8	232	14	6%
**Dermatoheliosis**	Larkin et al. (2020)	LPP	232	59,8	232	15	6,5%
**Skin cancer**							
**Skin cancer (overall)**	Lyakhovitsky et al. (2024)	LPP	126	47,92	89	4	3,2%
**Non–melanoma skin cancer**	Kusano & Mulinari–Brenner (2019)	FFA	38	61,1	38	3	7,89%
	Heppt et al. (2018)	FFA	72	62	70	2	2,8%
	Larkin et al. (2020)	LPP	232	59,8	232	16	3,1%
**Basal cell carcinoma**	Ormaechea–Pérez et al. (2016)	FFA	12	75	0	2	17%
	Lyakhovitsky et al. (2024)	LPP	126	47,92	89	3	2,4%
	Cantwell et al. (2021)	LPP	19	51,1	0	3	15,8%

Comorbidities	ID	Subtype	Study population, N	Mean age, y	Female, n	Events, n	Events, %
	Larkin et al. (2020)	LPP	232	59,8	232	11	4,7%
**Squamous cell carcinoma**	Larkin et al. (2020)	LPP	232	59,8	232	4	1,7%
**Melanoma**	Ormaechea–Pérez et al. (2016)	FFA	12	75	0	1	8,5%
	Heppt et al. (2018)	FFA	72	62	70	4	5,6%
	Lyakhovitsky et al. (2024)	LPP	126	47,92	89	1	0,8%
	Larkin et al. (2020)	LPP	232	59,8	232	5	2,2%
**Malignancies**							
**Non–skin malignancies**	Lyakhovitsky et al. (2024)	LPP	126	47,92	89	7	5,5%
**Breast cancer**	Meinhard et al. (2014)	FFA	32	NR	31	0	0%
	Saceda–Corralo et al. (2023)	FFA	103	61	103	8	7,7%
	Zhang et al. (2019)	FFA	29	55,4	28	6	21%
	Heppt et al. (2018)	FFA	72	62	70	3	4,2%
	Gkini et al. (2018)	FFA	40	65,9	40	1	2,5%
	Larkin et al. (2020)	LPP	232	59,8	232	11	4,7%
	Meinhard et al. (2014)	LPP	71	NR	59	2	3,4%
	Lyakhovitsky et al. (2024)	LPP	126	47,92	89	3	2,4%
**Cervical cancer**	Larkin et al. (2020)	LPP	232	59,8	232	9	3,9%
	Lyakhovitsky et al. (2024)	LPP	126	47,92	89	1	0,8%
**Prostate cancer**	Lobato–Berezo et al. (2022)	FFA	39	69	0	6	15,4%
	Tolkachjov et al. (2017)	FFA	7	54,6	0	1	14,3%

Comorbidities	ID	Subtype	Study population, N	Mean age, y	Female, n	Events, n	Events, %
	Ormaechea–Pérez et al. (2016)	FFA	12	75	0	1	8%
	Chaudhry et al. (2017)	LPP	99	47,8	0	7	7%
**Lung cancer**	Heppt et al. (2018)	FFA	72	62	70	1	1,4%
	Lyakhovitsky et al. (2024)	LPP	126	47,92	89	1	0,8%
**Leukemia**	Heppt et al. (2018)	FFA	72	62	70	1	1,4%
	Larkin et al. (2020)	LPP	232	59,8	232	6	2,6%
**Thyroid cancer**	Lyakhovitsky et al. (2024)	LPP	126	47,92	89	1	0,8%
	Larkin et al. (2020)	LPP	232	59,8	232	1	0,4%
**Vocal cord SCC**	Lyakhovitsky et al. (2024)	LPP	126	47,92	89	1	0,8%
**Thyroid diseases**							
**Thyroid disorder**	Meinhard et al. (2014)	FFA	32	NR	31	10	31,3%
	Ali et al. (2023)	FFA	38	53	38	9	23,7%
	Brito et al. (2023)	FFA	104	60,5	101	21	20,2%
	McSweeney et al. (2020)	FFA	711	66	711	88	12,9%
	Kanti et al. (2019)	FFA	490	65	467	182	37,1%
	Kusano & Mulinari–Brenner (2019)	FFA	38	61,1	38	14	36,84%
	Secchin et al. (2019)	FFA	16	62	16	6	37,5%
	Imhof et al. (2018)	FFA	148	62,1	148	68	45,9%
	Zhang et al. (2019)	FFA	29	55,4	28	13	45%
	Tolkachjov et al. (2017)	FFA	7	54,6	0	0	0%

Comorbidities	ID	Subtype	Study population, N	Mean age, y	Female, n	Events, n	Events, %
	Tan and Messenger (2009)	FFA	18	55,5	18	2	11%
	Samrao et al. (2010)	FFA	36	60	35	0	0%
	Starace et al. (2019)	FFA	65	62,5	65	18	27,7%
	Rayinda et al. (2022)	FFA	17	44	0	0	0%
	Alegre–Sánchez et al. (2017)	FFA	12	47,3	0	0	0%
	Adotama et al. (2021)	FFA	140	NR	140	14	10,1%
	Dorgham et al. (2022)	FFA	58	50,5	58	9	15,5%
	Lyakhovitsky et al. (2024)	LPP	126	47,92	89	20	15,9%
	Cantwell et al. (2021)	LPP	19	51,1	0	3	15,8%
	Larkin et al. (2020)	LPP	232	59,8	232	71	30,6%
	Chaudhry et al. (2017)	LPP	99	47,8	0	6	6%
	Meinhard et al. (2014)	LPP	71	NR	59	7	9,9%
**Thyroiditis**	Secchin et al. (2019)	FFA	16	62	16	2	12,5%
	Larkin et al. (2020)	LPP	232	59,8	232	6	3%
**Hypothyroidism**	García et al. (2023)	FFA	118	64	115	21	17,8%
	Doche et al. (2022)	FFA	33	53,1	0	3	9,1%
	Lobato–Berezo et al. (2022)	FFA	39	69	0	2	5,1%
	Rossi et al. (2021)	FFA	8	58,8	0	0	0%
	Grassi et al. (2021)	FFA	119	NR	111	21	17,6%
	Aslani et al. (2020)	FFA	22	46	22	9	40,9%

Comorbidities	ID	Subtype	Study population, N	Mean age, y	Female, n	Events, n	Events, %
	Maldonado Cid et al. (2020)	FFA	75	61	73	11	14,7%
	Kanti et al. (2019)	FFA	490	65	467	60	12,2%
	Kusano & Mulinari–Brenner (2019)	FFA	38	61,1	38	11	28,94%
	Valesky et al. (2019)	FFA	12	70,3	12	7	58,3%
	Secchin et al. (2019)	FFA	16	62	16	3	18,75%
	Imhof et al. (2018)	FFA	148	62,1	148	66	44,6%
	Pindado–Ortega et al. (2021)	FFA	224	61,2	222	38	16,9%
	Zhang et al. (2019)	FFA	29	55,4	28	10	35%
	Banka et al. (2014)	FFA	62	NR	61	13	21%
	Vañó–Galván et al. (2014)	FFA	355	61	343	52	15%
	Rocha et al. (2022)	FFA	27	63,8	26	7	25,9%
	MacDonald et al. (2012)	FFA	60	64	60	14	23%
	Strazzulla et al. (2018)	FFA	92	55	90	7	7,6%
	Ormaechea–Pérez et al. (2016)	FFA	12	75	0	0	0%
	Heppt et al. (2018)	FFA	72	62	70	21	29,2%
	Carmona–Rodriguez et al. (2023)	FFA	306	68,3	296	70	22,9%
	Lyakhovitsky et al. (2024)	LPP	126	47,92	89	15	17,2%
	Gharaei Nejad et al. (2023)	LPP	60	43,6	44	13	21,7%
	Larkin et al. (2020)	LPP	232	59,8	232	54	23,2%
**Hyperthyroidism**	Uzuncakmak et al. (2021)	FFA	16	51,9	16	1	6,3%

Comorbidities	ID	Subtype	Study population, N	Mean age, y	Female, n	Events, n	Events, %
	Kanti et al. (2019)	FFA	490	65	467	11	2,4%
	Suchonwanit et al. (2020)	FFA	56	51,3	54	2	3,6%
**Hashimoto thyroiditis**	Carrascoza et al. (2024)	FFA	138	NR	133	24	17,4%
	Uzuncakmak et al. (2021)	FFA	16	51,9	16	3	18,8%
	Kanti et al. (2019)	FFA	490	65	467	26	5,3%
	Secchin et al. (2019)	FFA	16	62	16	2	12,5%
	Imhof et al. (2018)	FFA	148	62,1	148	12	8,1%
	Saceda–Corralo et al. (2023)	FFA	103	61	103	11	10,7%
	Banka et al. (2014)	FFA	62	NR	61	3	5%
	Gkini et al. (2018)	FFA	40	65,9	40	1	2,5%
	Lyakhovitsky et al. (2024)	LPP	126	47,92	89	2	1,6%
	Larkin et al. (2020)	LPP	232	59,8	232	21	9%
	Carrascoza et al. (2024)	LPP	103	NR	68	17	16,5%
**Graves disease**	Imhof et al. (2018)	FFA	148	62,1	148	2	1,4%
	Saceda–Corralo et al. (2023)	FFA	103	61	103	3	2,9%
	Larkin et al. (2020)	LPP	232	59,8	232	6	3%
**Benign thyroid nodules**	Lyakhovitsky et al. (2024)	LPP	126	47,92	89	2	1,6%
	Cantwell et al. (2021)	LPP	19	51,1	0	5	26,3%
	Larkin et al. (2020)	LPP	232	59,8	232	4	3,1%
**Other thyroid conditions**	Kanti et al. (2019)	FFA	490	65	467	85	17,3%

Comorbidities	ID	Subtype	Study population, N	Mean age, y	Female, n	Events, n	Events, %
**Other endocrine disorders**							
**Hyperparathyroidism**	Carrascoza et al. (2024)	FFA	138	NR	133	0	0%
	Secchin et al. (2019)	FFA	16	62	16	1	6,25%
	Heppt et al. (2018)	FFA	72	62	70	1	1,4%
	Cantwell et al. (2021)	LPP	19	51,1	0	1	5,3%
	Carrascoza et al. (2024)	LPP	103	NR	68	1	0,9%
**Prolactinoma**	Valesky et al. (2019)	FFA	12	70,3	12	2	16,7%
**Growth hormone deficiency**	Cantwell et al. (2021)	LPP	19	51,1	0	1	5,3%
**Adrenal adenoma**	Larkin et al. (2020)	LPP	232	59,8	232	2	0,9%
**Gynecological / hormone–related disorders**							
**PCOS**	Imhof et al. (2018)	FFA	148	62,1	148	2	1,4%
	Oak et al. (2024)	LPP	24	48,4	24	1	4,2%
**PCOS/endometriosis**	Strazzulla et al. (2018)	FFA	92	55	90	2	2,2%
**Endometriosis**	Imhof et al. (2018)	FFA	148	62,1	148	11	7,4%
**Uterine leiomyoma**	Bala et al. (2025)	LPP	10	57	10	5	50%
**Erectile dysfunction**	Tolkachjov et al. (2017)	FFA	7	54,6	0	2	28,6%
	Rayinda et al. (2022)	FFA	17	44	0	1	6%
	Cantwell et al. (2021)	LPP	19	51,1	0	3	15,8%
	Chaudhry et al. (2017)	LPP	99	47,8	0	8	8,1%
**Hirsutism**	Imhof et al. (2018)	FFA	148	62,1	148	3	2%

Comorbidities	ID	Subtype	Study population, N	Mean age, y	Female, n	Events, n	Events, %
**Hypertrichosis**	Gkini et al. (2018)	FFA	40	65,9	40	1	2,5%
**Precocious puberty**	Cantwell et al. (2021)	LPP	19	51,1	0	1	5,3%
**Gastrointestinal diseases**							
**Gastrointestinal disease (overall)**	Lyakhovitsky et al. (2024)	LPP	126	47,92	89	10	7,9%
**Inflammatory bowel disease (overall)**	Imhof et al. (2018)	FFA	148	62,1	148	8	5,4%
**Crohn's disease**	Kanti et al. (2019)	FFA	490	65	467	1	0,2%
	Larkin et al. (2020)	LPP	232	59,8	232	4	1,7%
	Chaudhry et al. (2017)	LPP	99	47,8	0	1	1%
**Ulcerative colitis**	Moussa et al. (2022)	FFA	13	40,5	0	1	7,7%
	Kanti et al. (2019)	FFA	490	65	467	1	0,2%
	Zhang et al. (2019)	FFA	29	55,4	28	1	3%
	Larkin et al. (2020)	LPP	232	59,8	232	11	4,7%
**Other gastrointestinal diseases**							
**Celiac disease**	Ali et al. (2023)	FFA	38	53	38	1	2,6%
	McSweeney et al. (2020)	FFA	711	66	711	10	1,5%
	Kanti et al. (2019)	FFA	490	65	467	1	0,2%
	Imhof et al. (2018)	FFA	148	62,1	148	3	2%
	Lyakhovitsky et al. (2024)	LPP	126	47,92	89	1	0,8%
	Larkin et al. (2020)	LPP	232	59,8	232	8	3,4%

Comorbidities	ID	Subtype	Study population, N	Mean age, y	Female, n	Events, n	Events, %
**GERD**	Lyakhovitsky et al. (2024)	LPP	126	47,92	89	4	3,2%
	Oak et al. (2024)	LPP	24	48,4	24	4	16,7%
	Cantwell et al. (2021)	LPP	19	51,1	0	3	15,8%
	Larkin et al. (2020)	LPP	232	59,8	232	17	7,3%
**Duodenal ulcer**	Aslani et al. (2020)	FFA	22	46	22	1	4,5%
**Peptic ulcer**	Lyakhovitsky et al. (2024)	LPP	126	47,92	89	2	1,6%
**Irritable bowel syndrome**	Aslani et al. (2020)	FFA	22	46	22	1	4,5%
	Kanti et al. (2019)	FFA	490	65	467	3	0,6%
	Lyakhovitsky et al. (2024)	LPP	126	47,92	89	1	0,8%
**Gastrointestinal autoimmune diseases**	Larkin et al. (2020)	LPP	232	59,8	232	34	14,7%
**Pancreatitis**	Larkin et al. (2020)	LPP	232	59,8	232	2	0,9%
**Appendectomy**	Larkin et al. (2020)	LPP	232	59,8	232	6	2,6%
**Colon polyps**	Lyakhovitsky et al. (2024)	LPP	126	47,92	89	1	0,8%
	Larkin et al. (2020)	LPP	232	59,8	232	15	6,4%
**Intestinal obstruction**	Cantwell et al. (2021)	LPP	19	51,1	0	1	5,3%
**Diverticulitis**	Lyakhovitsky et al. (2024)	LPP	126	47,92	89	1	0,8%
	Oak et al. (2024)	LPP	24	48,4	24	1	4,2%
**Tubular adenoma**	Cantwell et al. (2021)	LPP	19	51,1	0	5	26,3%
**Liver disease**							
**Viral hepatitis**	Meinhard et al. (2014)	FFA	32	NR	31	0	0%

Comorbidities	ID	Subtype	Study population, N	Mean age, y	Female, n	Events, n	Events, %
	Meinhard et al. (2014)	LPP	71	NR	59	1	1,4%
**Hepatitis B**	Larkin et al. (2020)	LPP	232	59,8	232	11	4,7%
**Hepatitis C**	Brito et al. (2023)	FFA	104	60,5	101	4	3,9%
	Cantwell et al. (2021)	LPP	19	51,1	0	1	5,3%
	Larkin et al. (2020)	LPP	232	59,8	232	2	0,9%
	Chaudhry et al. (2017)	LPP	99	47,8	0	1	1%
**Autoimmune hepatitis**	Kanti et al. (2019)	FFA	490	65	467	1	0,2%
	Ormaechea–Pérez et al. (2016)	FFA	12	75	0	1	8%
**Hemochromatosis**	Valesky et al. (2019)	FFA	12	70,3	12	1	8,33%
**Hepatopathy**	Kusano & Mulinari–Brenner (2019)	FFA	38	61,1	38	4	10,52%
**Cholelithiasis**	García et al. (2023)	FFA	118	64	115	11	9,3%
	Cantwell et al. (2021)	LPP	19	51,1	0	1	5,3%
**Systemic autoimmune diseases**							
**Autoimmune (overall)**	Fernandes Melo et al. (2021)	FFA	38	55	37	4	13,8%
	McSweeney et al. (2020)	FFA	711	66	711	141	20,7%
	Kanti et al. (2019)	FFA	490	65	467	35	7,1%
	Pindado–Ortega et al. (2018)	FFA	103	55,8	103	11	10,7%
	Tolkachjov et al. (2017)	FFA	7	54,6	0	0	0%
	MacDonald et al. (2012)	FFA	60	64	60	18	30%
	Rayinda et al. (2022)	FFA	17	44	0	2	12%

Comorbidities	ID	Subtype	Study population, N	Mean age, y	Female, n	Events, n	Events, %
	Strazzulla et al. (2018)	FFA	92	55	90	10	10,9%
	Carmona–Rodriguez et al. (2023)	FFA	306	68,3	296	123	40,2%
	Lyakhovitsky et al. (2024)	LPP	126	47,92	89	9	7,1%
**Rheumatoid arthritis**	Aslani et al. (2020)	FFA	22	46	22	2	9,1%
	Kanti et al. (2019)	FFA	490	65	467	4	0,8%
	Valesky et al. (2019)	FFA	12	70,3	12	3	25%
	Imhof et al. (2018)	FFA	148	62,1	148	2	1,4%
	Banka et al. (2014)	FFA	62	NR	61	4	6%
	Larkin et al. (2020)	LPP	232	59,8	232	10	4,3%
**Ankylosing spondylitis**	Uzuncakmak et al. (2021)	FFA	16	51,9	16	1	6,3%
	Saceda–Corralo et al. (2023)	FFA	103	61	103	1	1%
**Mixed connective tissue disease**	Uzuncakmak et al. (2021)	FFA	16	51,9	16	1	6,3%
	Kanti et al. (2019)	FFA	490	65	467	1	0,2%
**Psoriatic arthritis**	Ali et al. (2023)	FFA	38	53	38	1	2,6%
	Kanti et al. (2019)	FFA	490	65	467	1	0,2%
**Polymyalgia rheumatica**	Kanti et al. (2019)	FFA	490	65	467	2	0,4%
**Polymyositis**	Banka et al. (2014)	FFA	62	NR	61	1	2%
**Polyarthritis**	Heppt et al. (2018)	FFA	72	62	70	6	8,3%
**Undifferentiated connective tissue disease**	Valesky et al. (2019)	FFA	12	70,3	12	1	8,33%

Comorbidities	ID	Subtype	Study population, N	Mean age, y	Female, n	Events, n	Events, %
**Sjögren syndrome**	Kanti et al. (2019)	FFA	490	65	467	1	0,2%
	Imhof et al. (2018)	FFA	148	62,1	148	6	4,1%
	Saceda–Corralo et al. (2023)	FFA	103	61	103	1	1%
	Zhang et al. (2019)	FFA	29	55,4	28	1	3%
	Starace et al. (2019)	FFA	65	62,5	65	2	3,1%
	MacDonald et al. (2012)	FFA	60	64	60	1	1,7%
	Lyakhovitsky et al. (2024)	LPP	126	47,92	89	2	1,6%
	Larkin et al. (2020)	LPP	232	59,8	232	12	5,2%
**Giant cell arteritis**	Kanti et al. (2019)	FFA	490	65	467	1	0,2%
	Heppt et al. (2018)	FFA	72	62	70	1	1,4%
**Osteoarthritis**	García et al. (2023)	FFA	118	64	115	11	9,3%
**Degenerative arthritis**	Cantwell et al. (2021)	LPP	19	51,1	0	3	15,8%
**Seronegative arthritis**	Cantwell et al. (2021)	LPP	19	51,1	0	1	5,3%
**Arthritis/joint pain**	Larkin et al. (2020)	LPP	232	59,8	232	41	17,6%
**Vasculitis**	Pindado–Ortega et al. (2018)	FFA	103	55,8	103	1	1%
**IgA vasculitis**	Lyakhovitsky et al. (2024)	LPP	126	47,92	89	1	0,8%
**Systemic lupus erythematosus**	Carrascoza et al. (2024)	FFA	138	NR	133	5	3,6%
	Kusano & Mulinari–Brenner (2019)	FFA	38	61,1	38	1	2,63%
	Secchin et al. (2019)	FFA	16	62	16	5	31,25%
	Imhof et al. (2018)	FFA	148	62,1	148	5	3,4%

Comorbidities	ID	Subtype	Study population, N	Mean age, y	Female, n	Events, n	Events, %
	Zhang et al. (2019)	FFA	29	55,4	28	1	3%
	Banka et al. (2014)	FFA	62	NR	61	2	3%
	Starace et al. (2019)	FFA	65	62,5	65	2	3,1%
	Adotama et al. (2021)	FFA	140	NR	140	3	2,2%
	Lyakhovitsky et al. (2024)	LPP	126	47,92	89	2	1,6%
	Oak et al. (2024)	LPP	24	48,4	24	1	4,2%
	Larkin et al. (2020)	LPP	232	59,8	232	2	0,9%
	Carrascoza et al. (2024)	LPP	103	NR	68	2	1,9%
**Cutaneous lupus**	Kanti et al. (2019)	FFA	490	65	467	7	1,4%
**Discoid lupus**	Secchin et al. (2019)	FFA	16	62	16	5	31,25%
	Saceda–Corralo et al. (2023)	FFA	103	61	103	1	1%
	Banka et al. (2014)	FFA	62	NR	61	2	3%
	Suchonwanit et al. (2020)	FFA	56	51,3	54	1	1,8%
**Dermatomyositis**	Carrascoza et al. (2024)	FFA	138	NR	133	0	0%
	Carrascoza et al. (2024)	LPP	103	NR	68	2	1,9%
**Morphea**	Pindado–Ortega et al. (2018)	FFA	103	55,8	103	1	1%
**Scleroderma/systemic sclerosis**	Carrascoza et al. (2024)	FFA	138	NR	133	1	0,7%
	Kanti et al. (2019)	FFA	490	65	467	1	0,2%
	Imhof et al. (2018)	FFA	148	62,1	148	2	1,4%
	Starace et al. (2019)	FFA	65	62,5	65	2	3,1%

Comorbidities	ID	Subtype	Study population, N	Mean age, y	Female, n	Events, n	Events, %
	Kanti et al. (2019)	FFA	490	65	467	2	0,4%
	Lyakhovitsky et al. (2024)	LPP	126	47,92	89	1	0,8%
	Carrascoza et al. (2024)	LPP	103	NR	68	2	1,9%
**Sarcoidosis**	Oak et al. (2024)	LPP	24	48,4	24	2	8,3%
**Other autoimmune**	Lyakhovitsky et al. (2024)	LPP	126	47,92	89	2	1,6%
**Other rheumatologic and musculoskeletal disorders**							
**Osteoporosis**	García et al. (2023)	FFA	118	64	115	8	6,8%
	Kusano & Mulinari–Brenner (2019)	FFA	38	61,1	38	1	2,63%
	Imhof et al. (2018)	FFA	148	62,1	148	23	15,5%
	Vañó–Galván et al. (2014)	FFA	355	61	343	31	9%
	Ladizinski et al. (2013)	FFA	19	55,9	19	6	31,6%
	Larkin et al. (2020)	LPP	232	59,8	232	28	12%
**Osteopenia**	Kusano & Mulinari–Brenner (2019)	FFA	38	61,1	38	1	2,63%
	Imhof et al. (2018)	FFA	148	62,1	148	25	16,9%
**Musculoskeletal pain**	Larkin et al. (2020)	LPP	232	59,8	232	20	8,6%
**Mechanical back pain**	Cantwell et al. (2021)	LPP	19	51,1	0	4	21,1%
**Fibromyalgia**	Lyakhovitsky et al. (2024)	LPP	126	47,92	89	2	1,6%
	Larkin et al. (2020)	LPP	232	59,8	232	8	3,4%
**Gout**	Lyakhovitsky et al. (2024)	LPP	126	47,92	89	1	0,8%
	Cantwell et al. (2021)	LPP	19	51,1	0	1	5,3%

Comorbidities	ID	Subtype	Study population, N	Mean age, y	Female, n	Events, n	Events, %
**Raynaud's syndrome**	Lyakhovitsky et al. (2024)	LPP	126	47,92	89	2	1,6%
**Carpal tunnel syndrome**	Larkin et al. (2020)	LPP	232	59,8	232	5	2,2%
**Pulmonary diseases**							
**Pulmonary disease (overall)**	Lyakhovitsky et al. (2024)	LPP	126	47,92	89	2	1,6%
**COPD**	Meinhard et al. (2014)	FFA	32	NR	31	0	0%
	Lyakhovitsky et al. (2024)	LPP	126	47,92	89	1	0,8%
	Larkin et al. (2020)	LPP	232	59,8	232	4	1,7%
	Meinhard et al. (2014)	LPP	71	NR	59	2	2,8%
**Obstructive sleep apnea**	Imhof et al. (2018)	FFA	148	62,1	148	9	6,1%
	Oak et al. (2024)	LPP	24	48,4	24	1	4,2%
	Cantwell et al. (2021)	LPP	19	51,1	0	2	10,5%
**Pulmonary nodule**	Cantwell et al. (2021)	LPP	19	51,1	0	2	10,5%
**Neurological disorders**							
**Neurologic disease (overall)**	Lyakhovitsky et al. (2024)	LPP	126	47,92	89	6	4,8%
**Migraine**	Aslani et al. (2020)	FFA	22	46	22	1	4,5%
	Lyakhovitsky et al. (2024)	LPP	126	47,92	89	2	1,6%
	Oak et al. (2024)	LPP	24	48,4	24	1	4,2%
	Larkin et al. (2020)	LPP	232	59,8	232	11	4,7%
**Epilepsy**	Carrascoza et al. (2024)	FFA	138	NR	133	0	0%

Comorbidities	ID	Subtype	Study population, N	Mean age, y	Female, n	Events, n	Events, %
	Ali et al. (2023)	FFA	38	53	38	1	2,6%
	Lyakhovitsky et al. (2024)	LPP	126	47,92	89	2	1,6%
	Carrascoza et al. (2024)	LPP	103	NR	68	1	0,9%
**Dementia**	Larkin et al. (2020)	LPP	232	59,8	232	5	2,2%
**Benign essential tremor**	Cantwell et al. (2021)	LPP	19	51,1	0	1	5,3%
**Neuropathy**	Larkin et al. (2020)	LPP	232	59,8	232	13	5,6%
**Meningioma**	Lyakhovitsky et al. (2024)	LPP	126	47,92	89	1	0,8%
	Oak et al. (2024)	LPP	24	48,4	24	1	4,2%
**Psychiatric disorders**							
**Anxiety**	Carrascoza et al. (2024)	FFA	138	NR	133	1	0,7%
	García et al. (2023)	FFA	118	64	115	10	8,5%
	Kusano & Mulinari–Brenner (2019)	FFA	38	61,1	38	3	7,89%
	Lyakhovitsky et al. (2024)	LPP	126	47,92	89	12	9,5%
	Cantwell et al. (2021)	LPP	19	51,1	0	1	5,3%
	Larkin et al. (2020)	LPP	232	59,8	232	97	41,8%
	Carrascoza et al. (2024)	LPP	103	NR	68	1	0,9%
**Depression**	Carrascoza et al. (2024)	FFA	138	NR	133	1	0,7%
	Meinhard et al. (2014)	FFA	32	NR	31	0	0%
	Kusano & Mulinari–Brenner (2019)	FFA	38	61,1	38	14	36,84%
	Imhof et al. (2018)	FFA	148	62,1	148	41	27,7%

Comorbidities	ID	Subtype	Study population, N	Mean age, y	Female, n	Events, n	Events, %
	Lyakhovitsky et al. (2024)	LPP	126	47,92	89	11	8,7%
	Cantwell et al. (2021)	LPP	19	51,1	0	2	10,5%
	Larkin et al. (2020)	LPP	232	59,8	232	106	45,7%
	Carrascoza et al. (2024)	LPP	103	NR	68	1	0,9%
	Meinhard et al. (2014)	LPP	71	NR	59	5	7%
**Anxiety/depression**	Doche et al. (2022)	FFA	33	53,1	0	4	12,1%
	Strazzulla et al. (2018)	FFA	92	55	90	5	5,4%
**Post–traumatic stress disorder**	Lyakhovitsky et al. (2024)	LPP	126	47,92	89	3	2,4%
**Schizoaffective disorder**	Lyakhovitsky et al. (2024)	LPP	126	47,92	89	1	0,8%
**Insomnia**	Lyakhovitsky et al. (2024)	LPP	126	47,92	89	14	11,1%
	Larkin et al. (2020)	LPP	232	59,8	232	68	29,3%
**Smoking**	Lyakhovitsky et al. (2024)	LPP	126	47,92	89	19	15,1%
**Renal / urologic disorders**							
**Renal/urologic disorders (overall)**	Lyakhovitsky et al. (2024)	LPP	126	47,92	89	1	0,8%
	Larkin et al. (2020)	LPP	232	59,8	232	19	8,2%
**Benign prostatic hyperplasia**	Lobato–Berezo et al. (2022)	FFA	39	69	0	13	33,3%
	Lyakhovitsky et al. (2024)	LPP	126	47,92	89	1	0,8%
**Prostatitis**	Cantwell et al. (2021)	LPP	19	51,1	0	2	10,5%
**Hypospadias**	Cantwell et al. (2021)	LPP	19	51,1	0	1	5,3%
**Nephrolithiasis**	Cantwell et al. (2021)	LPP	19	51,1	0	2	10,5%

Comorbidities	ID	Subtype	Study population, N	Mean age, y	Female, n	Events, n	Events, %
**IgA nephropathy**	Cantwell et al. (2021)	LPP	19	51,1	0	1	5,3%
**Focal segmental glomerulosclerosis**	Cantwell et al. (2021)	LPP	19	51,1	0	1	5,3%
**Infectious diseases**							
**Condyloma acuminatum**	Cantwell et al. (2021)	LPP	19	51,1	0	1	5,3%
**Verruca vulgaris**	Larkin et al. (2020)	LPP	232	59,8	232	5	2,2%
**Impetigo**	Larkin et al. (2020)	LPP	232	59,8	232	1	0,4%
**Tinea capitis**	Larkin et al. (2020)	LPP	232	59,8	232	1	0,4%
**Hematologic and nutritional disorders**							
**Anemia**	Imhof et al. (2018)	FFA	148	62,1	148	15	10,1%
	Gharaei Nejad et al. (2023)	LPP	60	43,6	44	12	20%
	Larkin et al. (2020)	LPP	232	59,8	232	11	4,7%
**Iron deficiency anemia**	Aslani et al. (2020)	FFA	22	46	22	1	4,5%
**Pernicious anemia**	McSweeney et al. (2020)	FFA	711	66	711	8	1,2%
	Saceda–Corralo et al. (2023)	FFA	103	61	103	1	1%
	MacDonald et al. (2012)	FFA	60	64	60	1	1,7%
	Larkin et al. (2020)	LPP	232	59,8	232	4	1,7%
**Autoimmune hemolytic anemia**	Lyakhovitsky et al. (2024)	LPP	126	47,92	89	1	0,8%
**Iron deficiency**	Meinhard et al. (2014)	FFA	32	NR	31	1	3,1%
	Meinhard et al. (2014)	LPP	71	NR	59	3	4,2%

Comorbidities	ID	Subtype	Study population, N	Mean age, y	Female, n	Events, n	Events, %
**Vitamin D deficiency**	Imhof et al. (2018)	FFA	148	62,1	148	16	10,8%
	Gharaei Nejad et al. (2023)	LPP	60	43,6	44	18	30%
	Larkin et al. (2020)	LPP	232	59,8	232	22	9,4%
	Conic et al. (2021)	LPP	58	56,6	55	12	21%
**Other diseases**							
**Erythema nodosum**	Pindado–Ortega et al. (2018)	FFA	103	55,8	103	2	1,9%
**Hyperhidrosis**	Pindado–Ortega et al. (2018)	FFA	103	55,8	103	1	1%
**Angular cheilitis**	Gkini et al. (2018)	FFA	40	65,9	40	1	2,5%
**Keratosis pilaris**	Carrascoza et al. (2024)	FFA	138	NR	133	1	0,7%
	Carrascoza et al. (2024)	LPP	103	NR	68	1	0,9%
**Asteatotic dermatitis**	Larkin et al. (2020)	LPP	232	59,8	232	2	0,9%
**Xerosis**	Larkin et al. (2020)	LPP	232	59,8	232	4	1,7%
**Ichthyosis hystrix (localized)**	Cantwell et al. (2021)	LPP	19	51,1	0	1	5,3%
**Xerostomia**	Larkin et al. (2020)	LPP	232	59,8	232	1	0,4%
**Intertrigo**	Larkin et al. (2020)	LPP	232	59,8	232	3	1,3%
**Ecchymosis**	Larkin et al. (2020)	LPP	232	59,8	232	1	0,4%
**Folliculitis**	Larkin et al. (2020)	LPP	232	59,8	232	1	0,4%
**Petechiae**	Larkin et al. (2020)	LPP	232	59,8	232	1	0,4%
**Steroid atrophy**	Larkin et al. (2020)	LPP	232	59,8	232	1	0,4%
**Schamberg purpura**	Larkin et al. (2020)	LPP	232	59,8	232	1	0,4%

Comorbidities	ID	Subtype	Study population, N	Mean age, y	Female, n	Events, n	Events, %
**Onychodystrophy**	Larkin et al. (2020)	LPP	232	59,8	232	5	2,2%
**Drug reaction hypersensitivity**	Larkin et al. (2020)	LPP	232	59,8	232	2	0,9%
**HIV**	Oak et al. (2024)	LPP	24	48,4	24	1	4,2%
**Cataracts**	Cantwell et al. (2021)	LPP	19	51,1	0	1	5,3%
**Vision problems**	Larkin et al. (2020)	LPP	232	59,8	232	15	6,5%
**Iritis**	Zhang et al. (2019)	FFA	29	55,4	28	1	3%

Supplementary Table S6. Sex-stratified pooled prevalence of comorbidities in FFA and classic LPP

This post hoc analysis includes comorbidities with a pooled estimate for at least one sex within each diagnosis. Data were obtained from single-sex samples or explicitly sex-disaggregated counts. Prevalence and I^2 are expressed as percentages. Random-effects estimates used inverse-variance pooling of logit-transformed proportions, DerSimonian–Laird estimation of between-study variance, and conventional normal-approximation confidence intervals. A continuity correction of 0.5 was applied to event and non-event counts in studies with zero or all events. Contributing studies may differ between sexes and outcomes; no formal between-sex comparisons were performed. Estimates based on few studies should be interpreted cautiously.

A. Frontal fibrosing alopecia (FFA)

Comorbidity	Women				Men			
	k	Events/N	Pooled prevalence, % [95% CI]	I^2 (%)	k	Events/N	Pooled prevalence, % [95% CI]	I^2 (%)
Acne	2	9/186	5.0 [2.6–9.3]	0.0	0	—	NC	NA
Alopecia areata	6	19/901	2.6 [1.3–5.5]	56.7	2	1/31	6.5 [1.1–29.9]	12.2
Androgenetic alopecia	7	221/672	27.2 [14.6–45.0]	91.9	9	82/137	58.0 [39.9–74.2]	66.2
Ankylosing spondylitis	2	2/119	2.5 [0.4–14.1]	43.5	0	—	NC	NA
Asthma	0	—	NC	NA	2	4/50	8.0 [3.0–19.5]	0.0
Atopic dermatitis	3	11/153	7.3 [4.1–12.7]	0.0	2	3/50	6.1 [2.0–17.2]	0.0
Atopy (general)	5	117/762	18.6 [6.0–44.9]	96.4	2	3/30	12.7 [1.7–55.0]	63.1
Autoimmune disease (overall)	4	204/1341	15.0 [8.0–26.5]	93.0	3	3/47	8.0 [2.8–20.7]	0.0
Autoimmune hepatitis	1	1/467	NC	NA	2	1/35	5.2 [1.0–22.3]	0.0
Breast cancer	2	9/143	6.3 [2.7–14.3]	17.9	0	—	NC	NA
Celiac disease	4	15/1364	1.3 [0.6–2.8]	31.5	1	0/23	NC	NA
Coexisting classic LPP	4	34/223	14.7 [8.5–24.1]	58.5	0	—	NC	NA
Contact dermatitis	1	8/103	NC	NA	2	1/50	4.1 [0.8–19.5]	7.4

Comorbidity	Women				Men			
	k	Events/N	Pooled prevalence, % [95% CI]	I² (%)	k	Events/N	Pooled prevalence, % [95% CI]	I² (%)
Coronary artery disease	2	30/505	6.0 [4.2–8.5]	0.0	1	1/23	NC	NA
Depression	2	55/186	30.1 [22.9–38.3]	16.9	0	—	NC	NA
Diabetes mellitus (types 1 and 2)	4	22/553	5.3 [2.8–9.8]	37.5	1	1/23	NC	NA
Diabetes type 1	2	5/609	0.8 [0.3–2.0]	0.0	1	0/23	NC	NA
Diabetes type 2	2	15/609	2.5 [1.5–4.1]	0.0	1	1/23	NC	NA
Discoid lupus	2	6/119	6.8 [0.2–75.8]	91.2	0	—	NC	NA
Dyslipidemia	8	207/884	20.5 [12.2–32.1]	88.4	4	7/56	12.8 [6.2–24.6]	0.0
Erectile dysfunction	0	—	NC	NA	2	3/24	14.8 [2.8–51.6]	48.9
Graves disease	2	5/251	2.1 [0.9–5.0]	0.0	0	—	NC	NA
Hashimoto disease	6	55/790	8.2 [5.5–12.0]	41.6	1	0/23	NC	NA
Hyperglycemia / Prediabetes	2	23/170	13.5 [9.2–19.5]	0.0	1	2/33	NC	NA
Hypertension	10	200/916	25.9 [17.1–37.1]	85.7	5	18/89	21.7 [14.1–32.0]	0.0
Hyperthyroidism	2	12/483	2.6 [1.5–4.4]	0.0	1	0/23	NC	NA
Hypothyroidism	9	241/1217	26.0 [17.1–37.6]	90.8	6	7/127	6.9 [3.5–12.9]	0.0
Irritable bowel syndrome	2	3/489	1.3 [0.1–11.8]	73.2	1	1/23	NC	NA
Lichen planus	10	123/1074	12.6 [6.4–23.4]	87.5	4	4/90	7.4 [3.1–16.8]	0.0
Lichen planus (cutaneous)	4	31/870	5.9 [0.3–52.8]	97.0	3	4/83	7.1 [2.2–20.3]	28.8
Lichen planus (genital)	3	40/877	7.5 [2.7–19.4]	88.5	1	0/37	NC	NA
Lichen planus (mucosal)	2	2/72	3.7 [0.7–16.6]	25.3	1	0/13	NC	NA
Lichen planus (nail)	2	3/749	0.8 [0.1–6.6]	70.1	2	0/70	1.4 [0.2–9.2]	0.0
Lichen planus (oral)	3	23/267	9.0 [6.0–13.1]	0.0	3	1/87	2.8 [0.7–10.7]	0.0
Lichen planus pigmentosus	3	20/92	21.0 [6.6–50.0]	75.9	1	1/33	NC	NA
Lichen sclerosus	4	23/331	6.9 [3.4–13.5]	50.1	2	3/25	14.0 [3.1–45.2]	38.3
Melasma	2	3/161	2.2 [0.7–7.1]	8.6	0	—	NC	NA
Mixed connective tissue disease	2	2/483	1.2 [0.0–25.6]	82.5	1	0/23	NC	NA
Osteopenia	2	26/186	8.6 [1.4–39.2]	73.6	0	—	NC	NA
Osteoporosis	3	30/205	15.2 [5.8–34.2]	71.0	0	—	NC	NA
Pernicious anemia	3	10/874	1.2 [0.6–2.1]	0.0	0	—	NC	NA
Prostate cancer	0	—	NC	NA	3	8/58	14.1 [7.2–25.7]	0.0

Comorbidity	Women				Men			
	k	Events/N	Pooled prevalence, % [95% CI]	I² (%)	k	Events/N	Pooled prevalence, % [95% CI]	I² (%)
Psoriasis	9	22/878	3.5 [1.5–7.7]	63.2	2	1/31	6.5 [1.1–29.9]	12.2
Psoriatic arthritis	2	1/505	0.6 [0.0–12.7]	71.0	1	1/23	NC	NA
Rheumatoid arthritis	4	11/649	4.2 [0.7–20.0]	86.9	1	0/23	NC	NA
Rosacea	6	78/826	10.5 [3.7–26.2]	93.3	5	31/115	28.1 [20.4–37.3]	0.0
Scleroderma/systemic sclerosis	2	4/213	2.0 [0.8–5.3]	0.0	0	—	NC	NA
Seborrheic dermatitis	3	33/328	6.5 [1.5–24.1]	87.5	1	2/7	NC	NA
Sjögren syndrome	5	11/843	1.7 [0.6–4.5]	54.1	1	0/23	NC	NA
Systemic lupus erythematosus	5	16/407	5.0 [1.5–14.9]	79.9	0	—	NC	NA
Telogen effluvium	2	24/198	12.7 [1.5–58.2]	95.2	0	—	NC	NA
Thyroid disorders	10	407/1699	24.2 [15.4–35.8]	94.1	4	3/59	8.7 [3.5–20.2]	0.0
Ulcerative colitis	1	0/467	NC	NA	2	2/36	5.8 [1.4–20.4]	0.0
Vitiligo	11	32/986	4.4 [2.6–7.2]	42.2	3	2/41	6.7 [1.9–20.9]	0.0

B. Classic lichen planopilaris (LPP)

Comorbidity	Women				Men			
	k	Events/N	Pooled prevalence, % [95% CI]	I² (%)	k	Events/N	Pooled prevalence, % [95% CI]	I² (%)
Asthma	2	11/256	4.3 [2.4–7.6]	0.0	1	21/215	NC	NA
Dyslipidemia	2	40/256	15.6 [11.7–20.6]	0.0	1	18/99	NC	NA
Gastroesophageal reflux disease	2	21/256	10.0 [4.4–21.0]	57.8	1	3/19	NC	NA
Hypertension	2	61/256	29.3 [14.4–50.5]	77.3	1	7/19	NC	NA
Migraine	2	12/256	4.7 [2.7–8.1]	0.0	0	—	NC	NA
Obesity	2	19/256	7.4 [4.8–11.3]	0.0	1	7/19	NC	NA
Systemic lupus erythematosus	2	3/256	1.6 [0.4–7.1]	40.2	0	—	NC	NA

Abbreviations and notes: FFA, frontal fibrosing alopecia; LPP, lichen planopilaris; CI, confidence interval; k, number of independent studies; Events/N, observed cases/participants with available sex-specific data; I^2, inconsistency statistic; NC, not calculated (fewer than two studies); NA, not applicable; —, no eligible sex-specific data.

Supplementary Table S7. Methodological quality assessment of included studies using the Joanna Briggs Institute checklist

JBI checklist items:

Q1, sample frame appropriate to address the target population;

Q2, study participants sampled appropriately;

Q3, sample size adequate;

Q4, study subjects and setting described in detail;

Q5, data analysis conducted with sufficient coverage of the identified sample;

Q6, valid methods used for identification of the condition;

Q7, condition measured in a standard, reliable way for all participants;

Q8, appropriate statistical analysis;

Q9, adequate response rate and management of low response rate.

JBI score: number of items rated as "Yes".

ID	Q1	Q2	Q3	Q4	Q5	Q6	Q7	Q8	Q9	JBI score
**ID 1**	Unclear	Yes	Unclear	Yes	Yes	Unclear	Yes	Yes	Unclear	5
**ID 2**	Yes	Yes	Yes	Yes	Yes	Yes	Yes	Yes	Unclear	8
**ID 3**	Unclear	Yes	Yes	Yes	Yes	Yes	Yes	Yes	Unclear	7
**ID 4**	Unclear	Yes	No	Yes	Yes	Yes	Yes	Yes	Unclear	6
**ID 5**	Unclear	Yes	Yes	Yes	Yes	Yes	Yes	Yes	Unclear	7
**ID 6**	Yes	Yes	Unclear	Yes	Yes	Unclear	Yes	Yes	Unclear	6
**ID 7**	Yes	Yes	Unclear	Yes	Yes	Yes	Yes	Yes	Unclear	7

ID	Q1	Q2	Q3	Q4	Q5	Q6	Q7	Q8	Q9	JBI score
**ID 8**	Unclear	Yes	No	Yes	Yes	Yes	Yes	Yes	Unclear	6
**ID 9**	Unclear	Yes	No	Yes	Yes	Yes	Yes	Yes	Unclear	6
**ID 10**	Yes	Yes	Unclear	Yes	Yes	Unclear	Yes	Yes	Unclear	6
**ID 11**	Yes / Unclear	Yes	Yes	Yes	Yes	Yes	Yes	Yes	Unclear	7
**ID 12**	Yes	Yes	Yes	Yes	Yes	Yes	Yes	Yes	Unclear	8
**ID 13**	Unclear	Yes	Unclear	Yes	Yes	Yes	Yes	Yes	Unclear	6
**ID 14**	Yes/Unclear	Yes	Yes	Yes	Yes	Yes	Yes	Yes	Unclear	7
**ID 15**	Yes	Yes	Yes	Yes	Yes	Yes	Yes	Yes	Unclear	8
**ID 16**	Unclear	Yes	Unclear	Yes	Yes	Unclear	Yes	Yes	Unclear	5
**ID 17**	No	Yes	No	Yes	Yes	Unclear	Yes	Yes	Unclear	5
**ID 18**	No	Yes	No	Yes	Yes	Yes	Yes	Yes	Unclear	6
**ID 19**	Yes/Unclear	Yes	Yes	Yes	Yes	Yes	Yes	Yes	Unclear	7
**ID 20**	Unclear	Yes	Yes	Yes	Yes	Yes	Yes	Yes	Unclear	7
**ID 21**	Unclear	Yes	Unclear	Yes	Yes	Yes	Yes	Yes	Unclear	6
**ID 22**	Unclear	Yes	No	Yes	Yes	Yes	Yes	Yes	Unclear	6
**ID 23**	Yes/Unclear	Yes	Yes	Yes	Yes	Yes	Yes	Yes	Unclear	7
**ID 24**	Unclear	Yes	Yes	Yes	Yes	Yes	Yes	Unclear	Unclear	6
**ID 25**	Unclear	Yes	No	Yes	Yes	Yes	Yes	Yes	Unclear	6
**ID 26**	Unclear	Yes	No	Yes	Yes	Yes	Yes	Yes	Unclear	6
**ID 27**	Yes	Yes	Yes	Yes	Yes	Yes	Yes	Yes	Unclear	8

ID	Q1	Q2	Q3	Q4	Q5	Q6	Q7	Q8	Q9	JBI score
**ID 28**	Yes / Unclear	Yes	Unclear	Yes	Yes	Yes	Yes	Yes	Unclear	6
**ID 29**	Yes / Unclear	Yes	Yes	Yes	Yes	Yes	Yes	Yes	Unclear	7
**ID 30**	Yes / Unclear	Yes	Yes	Yes	Yes	Yes	Yes	Yes	Unclear	7
**ID 31**	Unclear	Yes	No	Yes	Yes	Yes	Yes	Yes	Unclear	6
**ID 32**	Unclear	Yes	Yes	Yes	Yes	Unclear	Yes	Yes	Unclear	6
**ID 33**	Unclear	Yes	Yes	Yes	Yes	Yes	Yes	Yes	Unclear	7
**ID 34**	Yes	Yes	No / Unclear	Yes	Yes	Yes	Yes	Yes	Unclear	7
**ID 35**	Unclear	Yes	Unclear	Yes	Yes	Yes	Yes	Yes	Unclear	6
**ID 36**	Unclear	Yes	Yes	Yes	Yes	Yes	Yes	Yes	Unclear	7
**ID 37**	Unclear	Yes	No / Unclear	Yes	Yes	Yes	Yes	Yes	Unclear	6
**ID 38**	Unclear	Yes	Yes	Yes	Yes	Yes	Yes	Yes	Unclear	7
**ID 39**	Unclear	Yes	Yes	Yes	Yes	Yes	Yes	Yes	Unclear	7
**ID 40**	Yes	Yes	Yes	Yes	Yes	Yes	Yes	Yes	Unclear	8
**ID 41**	Yes	Yes	Yes	Yes	Yes	Yes	Yes	Yes	Unclear	8
**ID 42**	Unclear	Yes	Yes	Yes	Yes	Yes	Yes	Yes	Unclear	7
**ID 43**	Unclear	Yes	Yes	Yes	Yes	Yes	Yes	Yes	Unclear	7
**ID 44**	No	Yes	Yes	Yes	Yes	Yes	Yes	Yes	Unclear	7
**ID 45**	Unclear	Yes	Unclear	Yes	Yes	Yes	Yes	Yes	Unclear	6

ID	Q1	Q2	Q3	Q4	Q5	Q6	Q7	Q8	Q9	JBI score
**ID 46**	Unclear	Yes	Yes	Yes	Yes	Yes	Yes	Yes	Unclear	7
**ID 47**	No	Yes	No	Yes	Yes	Yes	Yes	Yes	Unclear	6
**ID 48**	Unclear	Yes	Yes	Yes	Yes	Yes	Unclear	Yes	Unclear	6
**ID 49**	Unclear	Yes	Yes	Yes	Yes	Yes	Yes	Yes	Unclear	7
**ID 50**	No / Unclear	Yes	No / Unclear	Yes	Yes	Yes	Yes	Yes	Unclear	6
**ID 51**	Unclear	Yes	Yes	Yes	Yes	Yes	Yes	Yes	Unclear	7
**ID 52**	No / Unclear	Yes	No	Yes	Yes	Yes	Yes	Yes	Unclear	6
**ID 53**	Unclear	Yes	Yes	Yes	Yes	Yes	Yes	Yes	Unclear	7
**ID 54**	Unclear	Yes	Yes	Yes	Yes	Yes	Yes	Yes	Unclear	7
**ID 55**	Unclear	Yes	Yes	Yes	Yes	Unclear	Yes	Yes	Unclear	6
**ID 56**	Yes /Unclear	Yes	Unclear	Yes	Yes	Yes	Yes	Yes	Unclear	6
**ID 57**	Unclear	Yes	Yes	Yes	Yes	Yes	Yes	Yes	Unclear	7
**ID 58**	Yes	Yes	Yes	Yes	Yes	Yes	Yes	Yes	Unclear	8
**ID 59**	Unclear	Yes	Yes	Yes	Yes	Yes	Yes	Yes	Unclear	7
**ID 60**	Unclear	Yes	Yes	Yes	Yes	Yes	Yes	Yes	Unclear	7

Supplementary Table S8. Summary of subgroup analyses exploring heterogeneity in comorbidities associated with FFA and LPP

Alopecia	Outcome/comorbidity	k	Pooled prevalence, % (95% CI)	Original I² (%)	Main candidate subgroup (levels)	p subgroup	BH-adjusted p (q)	Within-subgroup I²
FFA	Hypertension	22	23.6 (17.8–30.6)	86.0	Variable collection method: Chart review (k=12); Clinical database (k=5); Patient surveys (k=2); Prospective clinical registry (k=3)	0.032	0.130	59.7
FFA	Dyslipidemia	18	17.9 (13.4–23.5)	82.0	Mean age group: 55–64 (k=7); <55 (k=4); ≥65 (k=3); NR (k=4)	<0.001	**0.003**	41.0
FFA	Diabetes (1 and 2)	7	9.2 (3.9–19.9)	84.0	Single/multicenter: Multicenter (k=2); Single (k=5)	<0.001	**0.004**	38.1
FFA	Psoriasis	16	3.4 (2.1–5.4)	48.0	Variable collection method: Chart review (k=10); Clinical database (k=2); Patient surveys (k=2); Prospective clinical registry (k=2)	0.021	0.096	15.5
FFA	Rosacea	15	13.7 (8.2–22.0)	89.0	Study sample size group: 50–99 (k=4); <50 (k=6); ≥100 (k=5)	0.007	**0.045**	53.8
FFA	Vitiligo	17	3.3 (2.1–5.0)	44.0	Study sample size group: 50–99 (k=3); <50 (k=8); ≥100 (k=6)	0.001	**0.010**	7.6
FFA	Lichen planus	26	10.0 (7.1–13.7)	78.0	Variable collection method: Chart review (k=20); Clinical database (k=3); Patient surveys (k=3)	0.042	0.157	70.2
FFA	Lichen planus (cutaneous)	13	4.0 (1.7–9.3)	91.0	Mean age group: 55–64 (k=6); <55 (k=2); ≥65 (k=3); NR (k=2)	0.016	0.083	57.6
FFA	Lichen planus pigmentosus	11	19.6 (11.9–30.5)	81.0	Region: Africa (k=2); Asia (k=2); International (k=2); South America (k=5)	<0.001	**0.003**	51.4
FFA	Lichen planus (oral)	8	5.2 (3.0–8.8)	45.0	Gender: Men (k=3); Women (k=3); Women and men (k=2)	0.008	**0.048**	0.0
FFA	Depression	4	11.8 (3.6–32.4)	86.0	Gender: Women (k=2); Women and men (k=2)	<0.001	**<0.001**	8.4
FFA	Androgenetic alopecia	22	31.9 (23.5–41.7)	90.0	Female proportion group: 0% women (k=7); 80–98% women (k=10); ≈100% women (k=5)	0.006	**0.043**	82.6
FFA	LPP	9	13.1 (9.2–18.2)	62.0	Single/multicenter: Multicenter (k=2); Single (k=7)	0.037	0.142	45.0
FFA	Hypothyroidism	22	20.6 (16.3–25.7)	83.0	Gender: Men (k=4); Women (k=7); Women and men (k=11)	<0.001	**0.003**	58.6
FFA	Thyroid disorders	17	22.2 (15.5–30.9)	90.0	Gender: Men (k=3); Women (k=9); Women and men (k=5)	0.033	0.133	71.9
FFA	Hashimoto disease	8	9.1 (5.7–14.2)	72.0	Histology group: Partial (k=5); Yes (k=3)	<0.001	**<0.001**	4.6

Alopecia	Outcome/comorbidity	k	Pooled prevalence, % (95% CI)	Original I² (%)	Main candidate subgroup (levels)	p subgroup	BH-adjusted p (q)	Within-subgroup I²
LPP	Hypertension	7	21.2 (13.5–31.6)	83.0	Study sample size group: 50–99 (k=2); <50 (k=2); ≥100 (k=3)	0.014	0.076	60.3
LPP	Gastroesophageal reflux disease	4	8.5 (4.3–16.1)	61.0	Study sample size group: <50 (k=2); ≥100 (k=2)	0.038	0.145	29.2

Abbreviations: FFA, frontal fibrosing alopecia; LPP, lichen planopilaris; CI, confidence interval; I^2, heterogeneity statistic; pp, percentage points.

BH, Benjamini-Hochberg; FDR, false discovery rate. q values are P values adjusted using the BH procedure across all 582 computable subgroup-difference tests for FFA and LPP, including tests not displayed in this summary table. Analyses repeated across comorbidity categories were counted once. q < 0.05 indicates statistical significance after FDR correction. All subgroup analyses are exploratory and based on study-level characteristics.

Supplementary Table S9. Univariable meta-regression analyses exploring sources of between-study heterogeneity

The table shows the strongest candidate moderator for each outcome. Models were fitted using study-level proportions on the logit scale with random-effects meta-regression and Hartung-Knapp-type inference. Original I^2 refers to heterogeneity in the overall meta-analysis; residual I^2 refers to heterogeneity remaining after inclusion of the moderator. I^2 reduction was calculated as original I^2 minus residual I^2. Findings are exploratory and should not be interpreted as causal or patient-level associations.

Alopecia subtype	Outcome category / comorbidity	k	Original I^2 (%)	Best candidate moderator	n used	p value; FDR q value	Residual I^2; I^2 reduction
FFA	Hypertension	22	86.0	Log N	22	p=0.016; q=0.561	Residual I^2=83.5; reduction=2.5 pp
FFA	Dyslipidemia	18	82.0	Mean age raw	15	p=0.071; q=0.741	Residual I^2=80.5; reduction=1.5 pp
FFA	Psoriasis	16	48.0	Variable collection method	16	p=0.019; q=0.561	Residual I^2=7.7; reduction=40.3 pp
FFA	Rosacea	15	89.0	Female proportion group	15	p=0.015; q=0.561	Residual I^2=82.1; reduction=6.9 pp
FFA	Vitiligo	17	44.0	Log N	17	p=0.002; q=0.220	Residual I^2=0.0; reduction=44.0 pp
FFA	Lichen planus (cutaneous)	13	91.0	Log master sample size	13	p=0.010; q=0.561	Residual I^2=84.8; reduction=6.2 pp
FFA	Lichen planus pigmentosus	11	81.0	Region	11	p=0.053; q=0.740	Residual I^2=58.0; reduction=23.0 pp
FFA	Androgenetic alopecia	22	90.0	Mean age raw	18	p=0.027; q=0.643	Residual I^2=87.7; reduction=2.3 pp
FFA	Alopecia areata	14	66.0	Log master sample size	14	p=0.015; q=0.561	Residual I^2=46.8; reduction=19.2 pp
FFA	Hypothyroidism	22	83.0	Gender	22	p=0.002; q=0.220	Residual I^2=67.6; reduction=15.4 pp
FFA	Thyroid disorders	17	90.0	Gender	17	p=0.098; q=0.750	Residual I^2=88.4; reduction=1.6 pp

Notes: BH, Benjamini-Hochberg; FDR, false discovery rate; FFA, frontal fibrosing alopecia; I^2, percentage of variability attributable to between-study heterogeneity; LPP, lichen planopilaris; pp, percentage points.

Supplementary Table S10. Leave-one-out sensitivity analyses for pooled prevalence estimates in FFA and LPP

Alopecia subtype	Outcome / comorbidity	k	Original pooled prevalence, % (95% CI)	Original I² (%)	New pooled prevalence after omission (%)	Study omitted for largest prevalence change	New I² after omission (%)	Study omitted for largest I² reduction
FFA	Atopy (general)	6	19.8 [7.1–44.3]	96.0	28.4	Kanti et al. (2019)	76.8	Kanti et al. (2019)
FFA	Diabetes (1 and 2)	7	9.2 [3.9–19.9]	84.0	12.4	Kanti et al. (2019)	56.4	Kanti et al. (2019)
FFA	Obesity	3	16.9 [12.0–23.3]	27.0	12.7	Imhof et al. (2018)	0.0	Doche et al. (2022)
FFA	Lichen planus (oral)	8	5.2 [3.0–8.8]	45.0	7.0	Carmona-Rodriguez et al. (2023)	0.0	Imhof et al. (2018)
FFA	Breast cancer	5	6.9 [3.0–15.1]	60.0	5.7	Zhang et al. (2019)	0.0	Zhang et al. (2019)
FFA	Depression	4	11.8 [3.6–32.4]	86.0	26.4	Carrascoza et al. (2024)	69.4	Carrascoza et al. (2024)
FFA	Anxiety	3	5.0 [1.6–14.5]	66.0	8.3	Carrascoza et al. (2024)	0.0	Carrascoza et al. (2024)
FFA	Telogen effluvium	3	8.5 [1.4–38.4]	92.0	4.0	Dorgham et al. (2022)	0.0	Dorgham et al. (2022)
FFA	Systemic lupus erythematosus	8	4.4 [2.1–9.0]	67.0	3.1	Secchin et al. (2019)	0.0	Secchin et al. (2019)
FFA	Sjogren syndrome	6	1.9 [0.8–4.4]	45.0	3.1	Kanti et al. (2019)	0.0	Kanti et al. (2019)
FFA	Discoid lupus	4	4.3 [0.7–23.3]	84.0	6.7	Saceda-Corralo et al. (2023)	0.0	Secchin et al. (2019)
FFA	Hashimoto disease	8	9.1 [5.7–14.2]	72.0	10.6	Kanti et al. (2019)	39.2	Carrascoza et al. (2024)
FFA	Ulcerative colitis	3	1.8 [0.2–14.1]	73.0	5.1	Kanti et al. (2019)	0.0	Kanti et al. (2019)
FFA	Hyperparathyroidism	3	1.8 [0.4–7.9]	32.0	2.9	Carrascoza et al. (2024)	0.0	Secchin et al. (2019)
FFA	Celiac disease	4	1.3 [0.6–2.8]	34.0	1.0	Imhof et al. (2018)	0.0	Kanti et al. (2019)
LPP	Allergic rhinitis	3	6.0 [2.8–12.6]	65.0	3.1	Pathoulas et al. (2023)	0.0	Carrascoza et al. (2024)
LPP	Atopic dermatitis	3	3.1 [1.0–8.6]	76.0	5.1	Larkin et al. (2020)	41.1	Larkin et al. (2020)
LPP	Dyslipidemia	7	16.0 [11.3–22.3]	65.0	18.1	Carrascoza et al. (2024)	18.3	Carrascoza et al. (2024)
LPP	Obesity	5	12.7 [7.2–21.5]	72.0	9.3	Cantwell et al. (2021)	0.0	Cantwell et al. (2021)

Alopecia subtype	Outcome / comorbidity	k	Original pooled prevalence, % (95% CI)	Original I² (%)	New pooled prevalence after omission (%)	Study omitted for largest prevalence change	New I² after omission (%)	Study omitted for largest I² reduction
LPP	Seborrheic dermatitis	5	13.9 [5.4–31.3]	95.0	18.0	Meinhard et al. (2014)	56.0	Ratnaparkhi et al. (2016)
LPP	Vitiligo	6	1.5 [0.6–3.4]	28.0	0.9	Gharaei Nejad et al. (2023)	0.0	Gharaei Nejad et al. (2023)
LPP	Lichen planus	6	11.1 [8.0–15.3]	40.0	12.4	Mardones & Shapiro (2017)	0.0	Mardones & Shapiro (2017)
LPP	Depression	5	9.6 [2.3–32.2]	95.0	14.6	Carrascoza et al. (2024)	39.6	Larkin et al. (2020)
LPP	Anxiety	4	9.0 [1.9–33.9]	94.0	15.8	Carrascoza et al. (2024)	62.1	Larkin et al. (2020)
LPP	Basal cell carcinoma	3	5.5 [2.2–13.2]	66.0	7.9	Lyakhovitsky et al. (2024)	14.1	Cantwell et al. (2021)
LPP	Thyroid disorders	5	14.5 [7.4–26.3]	87.0	17.8	Chaudhry et al. (2017)	45.3	Larkin et al. (2020)
LPP	Hypothyroidism	3	18.7 [12.2–27.5]	70.0	22.9	Lyakhovitsky et al. (2024)	0.0	Lyakhovitsky et al. (2024)
LPP	Benign thyroid nodules	3	4.5 [0.6–27.9]	90.0	1.7	Cantwell et al. (2021)	0.0	Cantwell et al. (2021)
LPP	Vitamin D deficiency	3	18.3 [8.4–35.2]	88.0	25.6	Larkin et al. (2020)	25.2	Larkin et al. (2020)
LPP	Androgenetic alopecia	3	27.2 [10.3–55.0]	86.0	38.6	Carrascoza et al. (2024)	47.3	Oak et al. (2024)

Abbreviations and notes: FFA, frontal fibrosing alopecia; LPP, lichen planopilaris; CI, confidence interval; I^2, inconsistency statistic; pp, percentage points.

Supplementary Table S11. PRISMA 2020 Checklist.

Section and Topic	Item #	Checklist item	Location where item is reported
**TITLE**			
Title	1	Identify the report as a systematic review.	*Title page*
**ABSTRACT**			
Abstract	2	See the PRISMA 2020 for Abstracts checklist.	Abstract
**INTRODUCTION**			
Rationale	3	Describe the rationale for the review in the context of existing knowledge.	*Introduction (paragraphs 1–3)*
Objectives	4	Provide an explicit statement of the objective(s) or question(s) the review addresses.	*Introduction (final paragraph)*
**METHODS**			
Eligibility criteria	5	Specify the inclusion and exclusion criteria for the review and how studies were grouped for the syntheses.	Methods 2.2
Information sources	6	Specify all databases, registers, websites, organisations, reference lists and other sources searched or consulted to identify studies. Specify the date when each source was last searched or consulted.	Methods 2.3
Search strategy	7	Present the full search strategies for all databases, registers and websites, including any filters and limits used.	Methods 2.3; Supplementary Table S1
Selection process	8	Specify the methods used to decide whether a study met the inclusion criteria of the review, including how many reviewers screened each record and each report retrieved, whether they worked independently, and if applicable, details of automation tools used in the process.	Methods 2.4
Data collection process	9	Specify the methods used to collect data from reports, including how many reviewers collected data from each report, whether they worked independently, any processes for obtaining or confirming data from study investigators, and if applicable, details of automation tools used in the process.	Methods 2.4
Data items	10a	List and define all outcomes for which data were sought. Specify whether all results that were compatible with each outcome domain in each study were sought (e.g. for all measures, time points, analyses), and if not, the methods used to decide which results to collect.	Methods 2.6
	10b	List and define all other variables for which data were sought (e.g. participant and intervention characteristics, funding sources). Describe any assumptions made about any missing or unclear information.	Methods 2.4
Study risk of bias assessment	11	Specify the methods used to assess risk of bias in the included studies, including details of the tool(s) used, how many reviewers assessed each study and whether they worked independently, and if applicable, details of automation tools used in the process.	Methods 2.5; Supplementary Table S7
Effect measures	12	Specify for each outcome the effect measure(s) (e.g. risk ratio, mean difference) used in the synthesis or presentation of results.	Methods 2.6; Methods 2.9
Synthesis methods	13a	Describe the processes used to decide which studies were eligible for each synthesis (e.g. tabulating the study intervention characteristics and comparing against the planned groups for each synthesis (item #5)).	Methods 2.6
	13b	Describe any methods required to prepare the data for presentation or synthesis, such as handling of missing summary statistics, or data conversions.	Methods 2.6

Section and Topic	Item #	Checklist item	Location where item is reported
	13c	Describe any methods used to tabulate or visually display results of individual studies and syntheses.	Methods 2.6; Methods 2.9; Figures 2–3 and Supplementary Figure S4
	13d	Describe any methods used to synthesize results and provide a rationale for the choice(s). If meta-analysis was performed, describe the model(s), method(s) to identify the presence and extent of statistical heterogeneity, and software package(s) used.	Methods 2.6; Methods 2.11
	13e	Describe any methods used to explore possible causes of heterogeneity among study results (e.g. subgroup analysis, meta-regression).	Methods 2.8; Supplementary Tables S8–S9
	13f	Describe any sensitivity analyses conducted to assess robustness of the synthesized results.	Methods 2.8; Results 3.5; Supplementary Table S10
Reporting bias assessment	14	Describe any methods used to assess risk of bias due to missing results in a synthesis (arising from reporting biases).	Methods 2.7 — Assessment of Small-Study Effects (Egger's test for syntheses with ≥10 studies)
Certainty assessment	15	Describe any methods used to assess certainty (or confidence) in the body of evidence for an outcome.	Not formally assessed; see Methods 2.5 and Discussion (limitations paragraphs)
**RESULTS**			
Study selection	16a	Describe the results of the search and selection process, from the number of records identified in the search to the number of studies included in the review, ideally using a flow diagram.	Results 3.1; Figure 1
	16b	Cite studies that might appear to meet the inclusion criteria, but which were excluded, and explain why they were excluded.	Results 3.1; Supplementary Table S3
Study characteristics	17	Cite each included study and present its characteristics.	Results 3.1; Supplementary Table S4; Supplementary Figures S1–S3
Risk of bias in studies	18	Present assessments of risk of bias for each included study.	Results 3.2; Supplementary Table S7
Results of individual studies	19	For all outcomes, present, for each study: (a) summary statistics for each group (where appropriate) and (b) an effect estimate and its precision (e.g. confidence/credible interval), ideally using structured tables or plots.	Results 3.3; Results 3.4; Supplementary Table S5
Results of syntheses	20a	For each synthesis, briefly summarize the characteristics and risk of bias among contributing studies.	Results 3.1; Results 3.2
	20b	Present results of all statistical syntheses conducted. If meta-analysis was done, present for each the summary estimate and its precision (e.g. confidence/credible interval) and measures of statistical heterogeneity. If comparing groups, describe the direction of the effect.	Results 3.3; Results 3.4; Tables 1–2; Figures 2–3 and Supplementary Figure S4
	20c	Present results of all investigations of possible causes of heterogeneity among study results.	Results 3.5; Supplementary Tables S8–S9
	20d	Present results of all sensitivity analyses conducted to assess the robustness of the synthesized results.	Results 3.5; Supplementary Table

Section and Topic	Item #	Checklist item	Location where item is reported
			S10
Reporting biases	21	Present assessments of risk of bias due to missing results (arising from reporting biases) for each synthesis assessed.	Results 3.3; Results 3.4; Tables 1–2. Egger's test was reported for syntheses with ≥10 studies; otherwise not assessed.
Certainty of evidence	22	Present assessments of certainty (or confidence) in the body of evidence for each outcome assessed.	Not formally assessed; see Discussion (limitations paragraphs)
**DISCUSSION**			
Discussion	23a	Provide a general interpretation of the results in the context of other evidence.	Discussion (paragraphs 1–4)
	23b	Discuss any limitations of the evidence included in the review.	Discussion (limitations paragraphs)
	23c	Discuss any limitations of the review processes used.	Discussion (limitations paragraphs)
	23d	Discuss implications of the results for practice, policy, and future research.	Discussion (clinical implications and future research)
**OTHER INFORMATION**			
Registration and protocol	24a	Provide registration information for the review, including register name and registration number, or state that the review was not registered.	Methods 2.1
	24b	Indicate where the review protocol can be accessed, or state that a protocol was not prepared.	PROSPERO registry (CRD420250656067)
	24c	Describe and explain any amendments to information provided at registration or in the protocol.	No amendments
Support	25	Describe sources of financial or non-financial support for the review, and the role of the funders or sponsors in the review.	Funding / Acknowledgements section
Competing interests	26	Declare any competing interests of review authors.	Conflicts of Interest section
Availability of data, code and other materials	27	Report which of the following are publicly available and where they can be found: template data collection forms; data extracted from included studies; data used for all analyses; analytic code; any other materials used in the review.	Data Availability Statement

SUPPLEMENTARY FIGURES

Supplementary Figure S1. Temporal evolution of included studies.

Bar plot illustrating the number of included studies by year of publication.

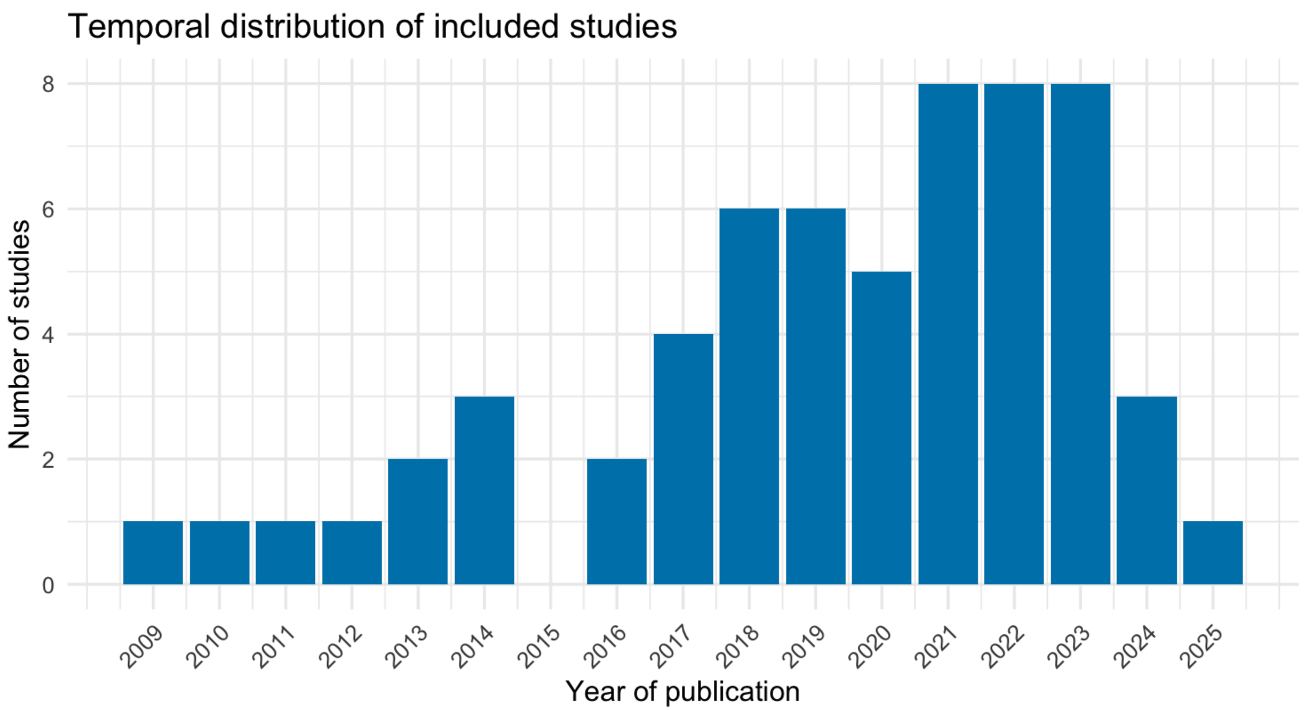

Supplementary Figure S2. Geographic distribution of included studies

Bar plot showing the number of single-country studies included in the systematic review, stratified by country of origin. Multinational studies were excluded from this analysis.

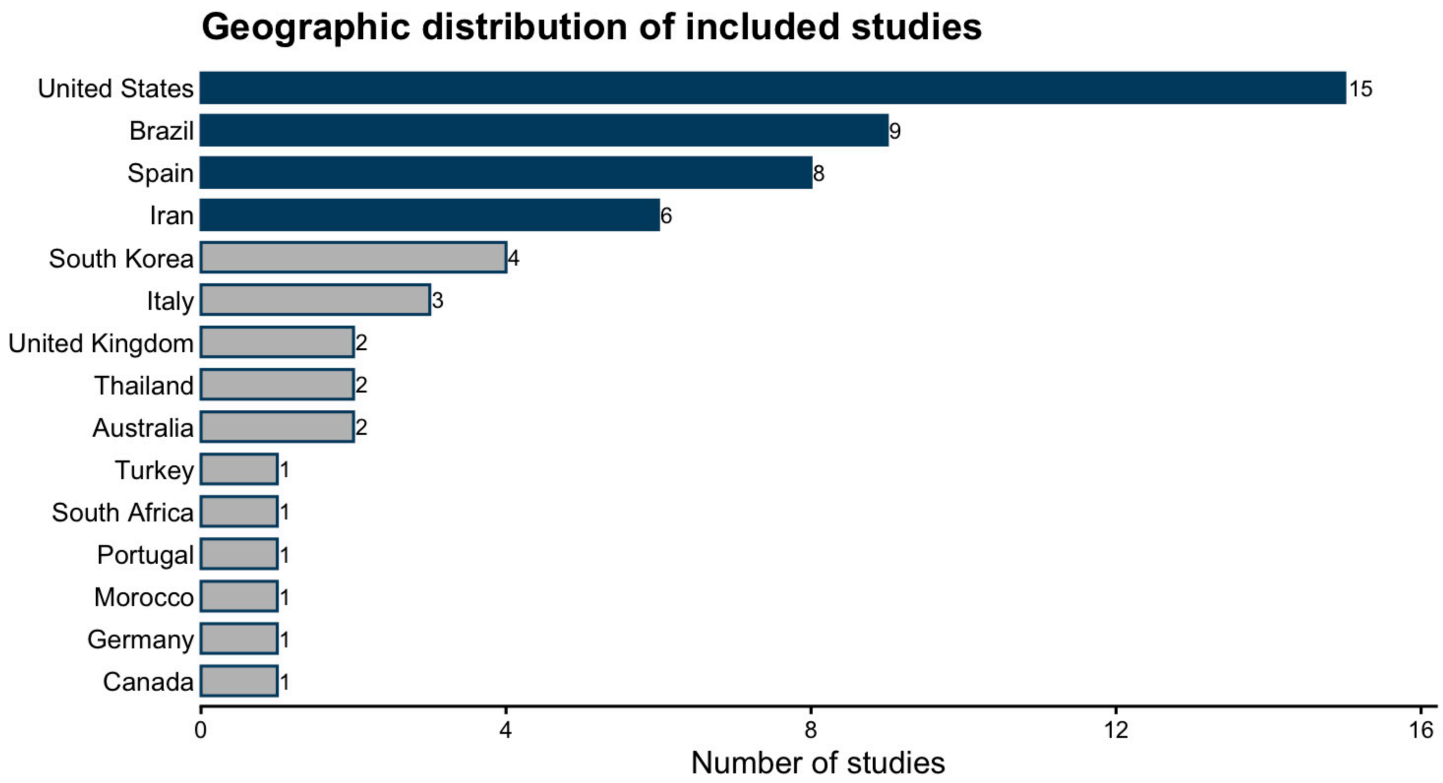

Supplementary Figure S3. Frequency of reported comorbidity domains

(A) Overall frequency of reported comorbidity domains across included studies, showing the number of studies reporting at least one comorbidity within each predefined domain. Autoimmune/immune-mediated and endocrine–thyroid comorbidities were the most frequently reported domains.

(B) Distribution of the number of comorbidity domains reported per study. Most studies reported one to three comorbidity domains, highlighting substantial heterogeneity in the breadth of comorbidity assessment across the literature (dashed line indicates the median number of domains per study).

(C) Heatmap depicting the relationship between study design and reported comorbidity domains. Cell values indicate the number of studies within each study design–domain combination, illustrating that case series dominate across most domains.

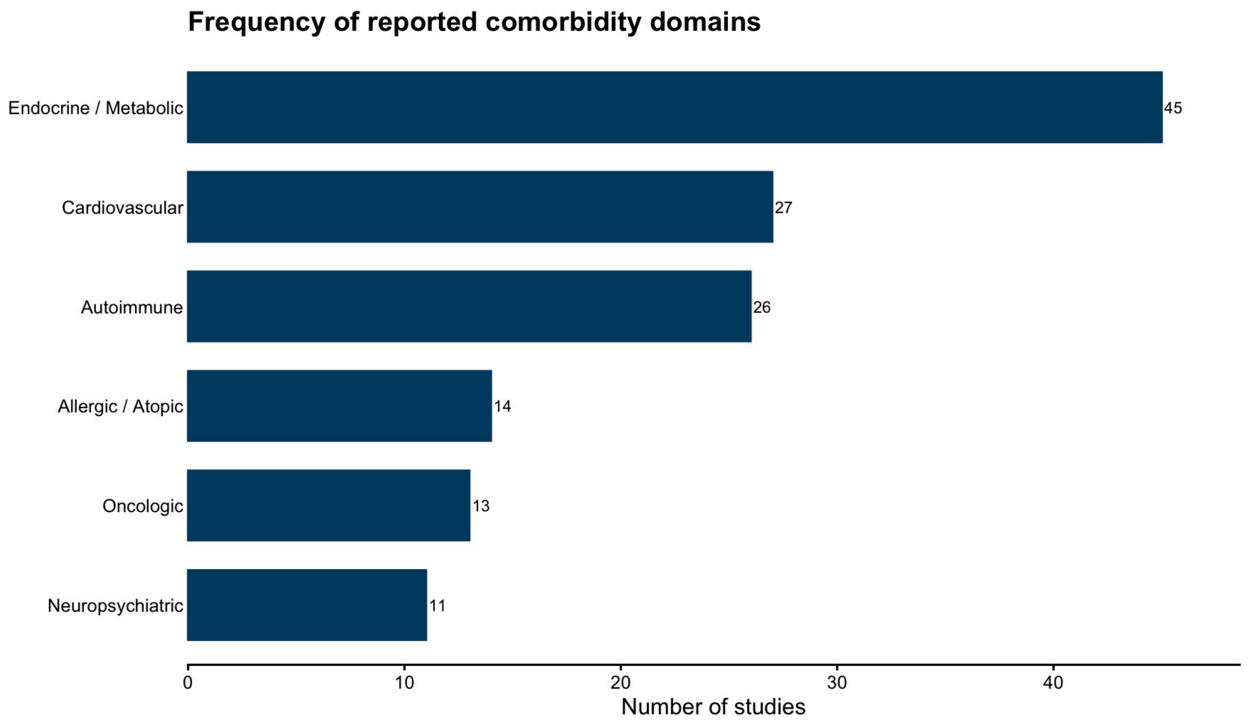
Frequency of reported comorbidity domains
Endocrine / Metabolic
45
Cardiovascular
27
Autoimmune
26
Allergic / Atopic
14
Oncologic
13
Neuropsychiatric
11
0
10
20
30
40
Number of studies

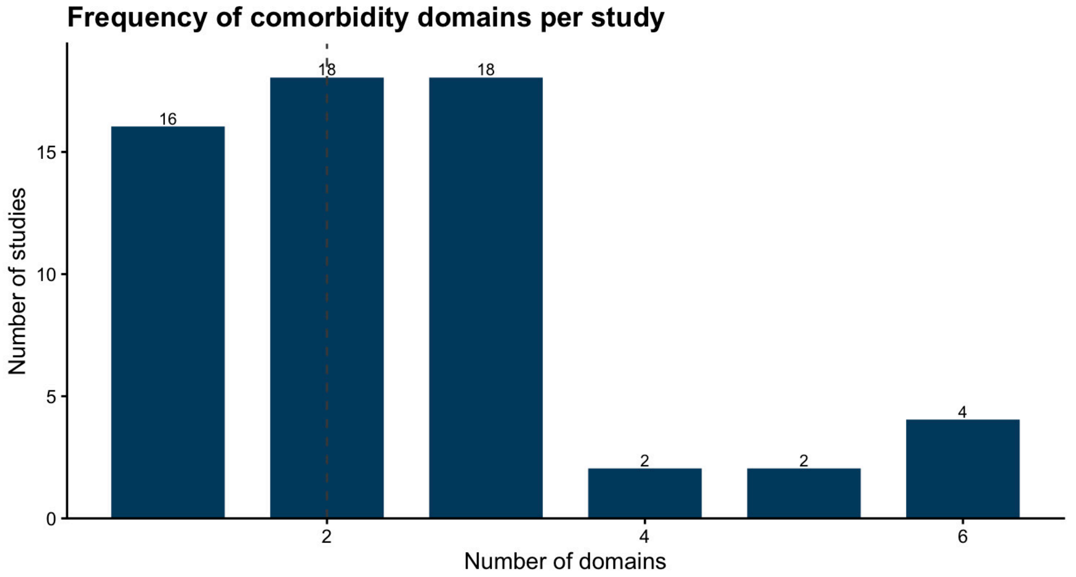
Frequency of comorbidity domains per study
Number of studies
Number of domains
0
5
10
15
2
4
6
16
18
18
2
2
4

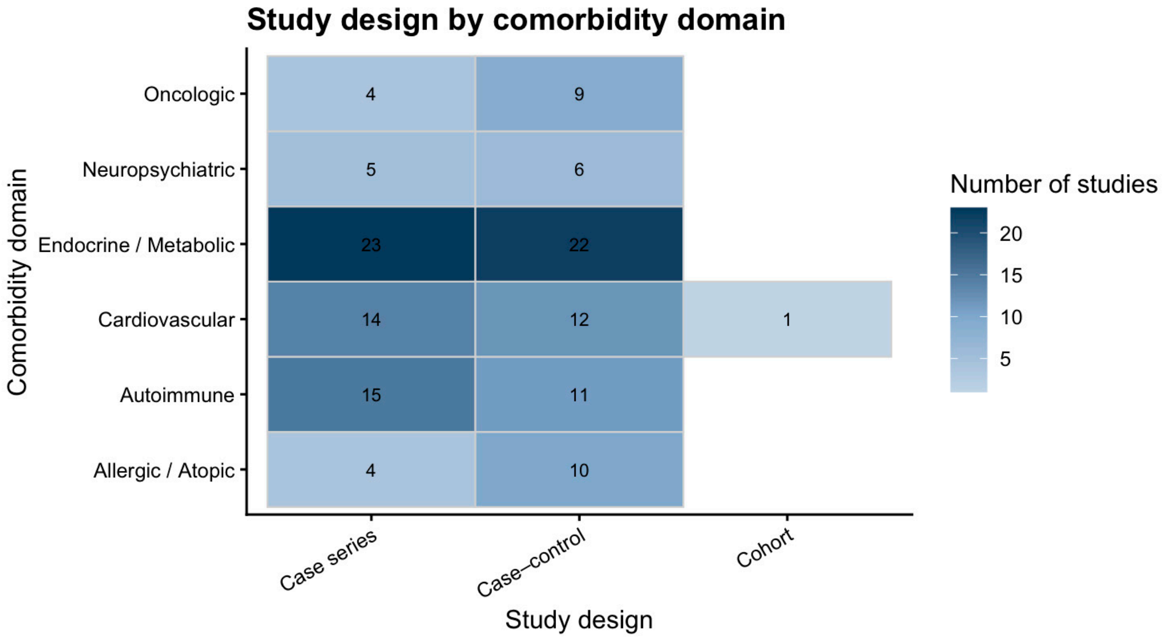
Study design by comorbidity domain
Comorbidity domain
Oncologic
Neuropsychiatric
Endocrine / Metabolic
Cardiovascular
Autoimmune
Allergic / Atopic
4
9
5
6
23
22
14
12
1
15
11
4
10
Case series
Case–control
Cohort
Study design
Number of studies
20
15
10
5

Supplementary Figure S4. Exploratory indirect comparison of pooled prevalence estimates in frontal fibrosing alopecia and lichen planopilaris

(A) Exploratory relative prevalence estimates based on pooled prevalence data. Points represent prevalence ratios and horizontal lines indicate 95% confidence intervals. The vertical dashed line denotes a prevalence ratio of 1, indicating no difference between conditions. Values >1 indicate higher pooled prevalence in FFA, whereas values <1 indicate higher pooled prevalence in LPP.

(B) Heatmap depicting immune pathway-oriented annotation of selected comorbidities according to canonical T helper axes (Th1, Th17, Th2 and Th22). Color intensity represents the logarithm of the relative prevalence estimate. This annotation is descriptive and hypothesis-generating and should not be interpreted as evidence of shared pathogenesis.

Estimates based on a limited number of studies or showing substantial between-study heterogeneity should be interpreted with caution.

FFA, frontal fibrosing alopecia; LPP, lichen planopilaris; CI, confidence interval; CV, cardiovascular.

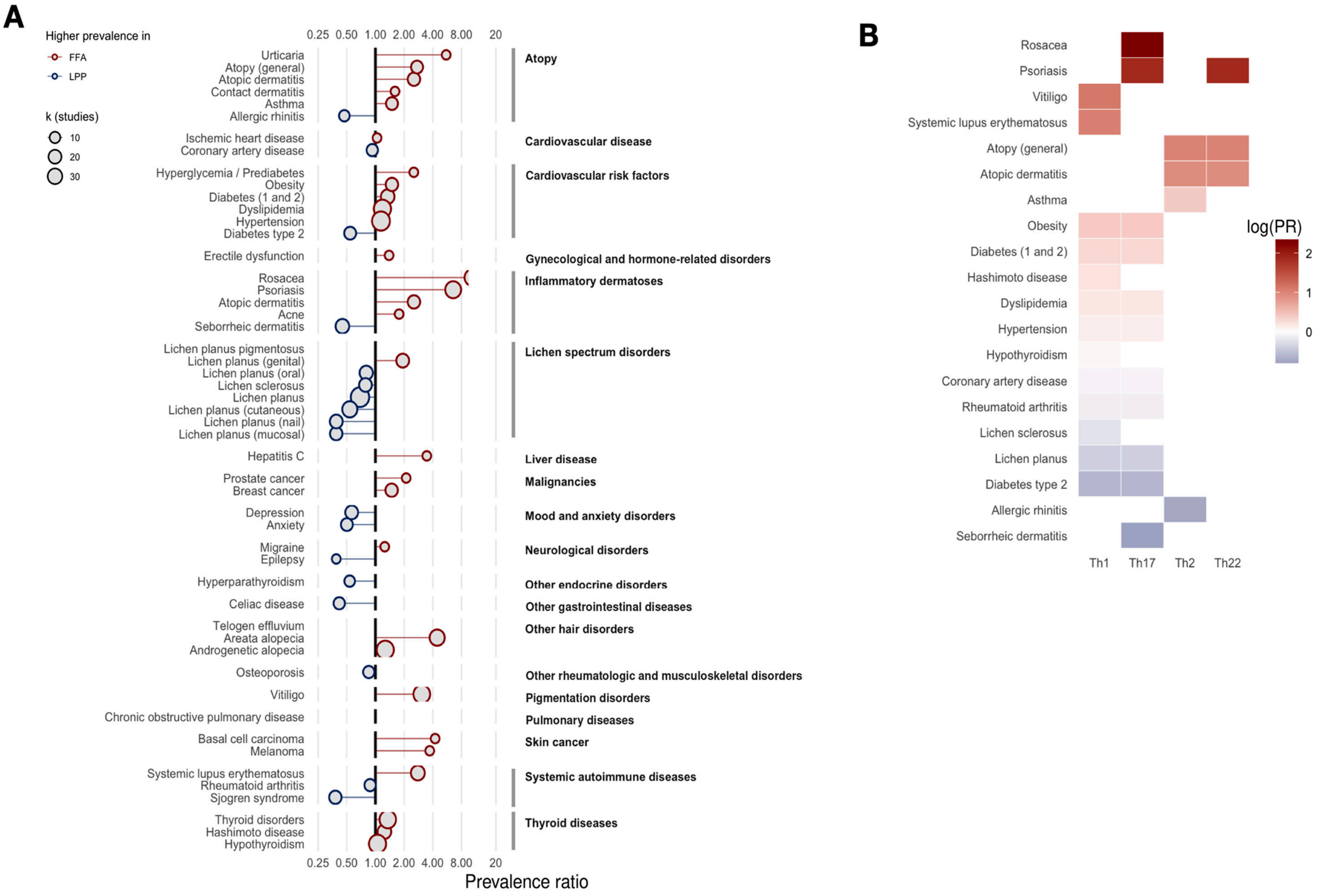
A
Higher prevalence in
FFA
LPP
k (studies)
10
20
30
0.25 0.50 1.00 2.00 4.00 8.00 20
Urticaria
Atopy (general)
Atopic dermatitis
Contact dermatitis
Asthma
Allergic rhinitis
Atopy
Ischemic heart disease
Coronary artery disease
Cardiovascular disease
Hyperglycemia / Prediabetes
Obesity
Diabetes (1 and 2)
Dyslipidemia
Hypertension
Diabetes type 2
Cardiovascular risk factors
Erectile dysfunction
Gynecological and hormone-related disorders
Rosacea
Psoriasis
Atopic dermatitis
Acne
Seborrheic dermatitis
Inflammatory dermatoses
Lichen planus pigmentosus
Lichen planus (genital)
Lichen planus (oral)
Lichen sclerosus
Lichen planus
Lichen planus (cutaneous)
Lichen planus (nail)
Lichen planus (mucosal)
Lichen spectrum disorders
Hepatitis C
Liver disease
Prostate cancer
Breast cancer
Malignancies
Depression
Anxiety
Mood and anxiety disorders
Migraine
Epilepsy
Neurological disorders
Hyperparathyroidism
Other endocrine disorders
Celiac disease
Other gastrointestinal diseases
Telogen effluvium
Areata alopecia
Androgenetic alopecia
Other hair disorders
Osteoporosis
Other rheumatologic and musculoskeletal disorders
Vitiligo
Pigmentation disorders
Chronic obstructive pulmonary disease
Pulmonary diseases
Basal cell carcinoma
Melanoma
Skin cancer
Systemic lupus erythematosus
Rheumatoid arthritis
Sjogren syndrome
Systemic autoimmune diseases
Thyroid disorders
Hashimoto disease
Hypothyroidism
Thyroid diseases
Prevalence ratio
B
Rosacea
Psoriasis
Vitiligo
Systemic lupus erythematosus
Atopy (general)
Atopic dermatitis
Asthma
Obesity
Diabetes (1 and 2)
Hashimoto disease
Dyslipidemia
Hypertension
Hypothyroidism
Coronary artery disease
Rheumatoid arthritis
Lichen sclerosus
Lichen planus
Diabetes type 2
Allergic rhinitis
Seborrheic dermatitis
Th1 Th17 Th2 Th22
log(PR)
2
1
0
